# Effectiveness, Core Elements, and Moderators of Response of Cognitive Remediation for Schizophrenia: A Systematic Review and Meta-analysis of Randomized Clinical Trials

**DOI:** 10.1001/jamapsychiatry.2021.0620

**Published:** 2021-04-20

**Authors:** Antonio Vita, Stefano Barlati, Anna Ceraso, Gabriele Nibbio, Cassandra Ariu, Giacomo Deste, Til Wykes

**Affiliations:** 1 Department of Clinical and Experimental Sciences, University of Brescia, Brescia, Italy; 2 Department of Mental Health and Addiction Services, ASST Spedali Civili of Brescia, Brescia, Italy; 3 Department of Psychology, Institute of Psychiatry, Psychology and Neuroscience, King’s College London, London, United Kingdom; 4 South London and Maudsley NHS Foundation Trust, Maudsley Hospital, London, United Kingdom

## Abstract

**Importance:**

Cognitive impairment is a core feature of schizophrenia, with negative consequences on functional outcomes. Although cognitive remediation (CR) is effective and mentioned in treatment guidance for schizophrenia, its active ingredients and ideal candidates are still debated.

**Objective:**

To provide a comprehensive update on CR effectiveness for cognition and functioning in schizophrenia and analyze the core ingredients of efficacy and role of patient characteristics.

**Data Sources:**

The reference list of the last comprehensive meta-analysis in 2011 was screened against eligibility criteria. Then, electronic databases (PubMed, Scopus, and PsycInfo) were systematically searched for articles published from January 2011 to February 2020. Reference lists of included articles and relevant reviews were hand searched, and Google Scholar was manually inspected.

**Study Selection:**

Eligible studies were randomized clinical trials comparing CR with any other control condition in patients diagnosed with schizophrenia spectrum disorders (with an unrestricted clinical status). Screening was performed by at least 2 independent reviewers.

**Data Extraction and Synthesis:**

The PRISMA guidelines were followed. Study data were independently extracted and pooled using random-effect models. Cohen *d* was used to measure outcomes. Trial methodological quality was evaluated with the Clinical Trials Assessment Measure.

**Main Outcomes and Measures:**

Primary outcomes were changes in global cognition and overall functioning from baseline to after treatment, subsequently investigated through metaregressions, subgroup, and sensitivity analyses based on prespecified hypotheses, to identify potential moderators of response associated with treatment modality and patient characteristics.

**Results:**

Of 1815 identified reports, 358 full texts were assessed and 194 reports on 130 studies were included. Based on 130 studies with 8851 participants, CR was effective on cognition (*d*, 0.29 [95% CI, 0.24-0.34]) and functioning (*d*, 0.22 [95% CI, 0.16-0.29]). An active and trained therapist (cognition: χ^2^_1_, 4.14; *P* = .04; functioning: χ^2^_1_, 4.26; *P* = .04), structured development of cognitive strategies (cognition: χ^2^_1_, 9.34; *P* = .002; functioning: χ^2^_1_, 8.12; *P* = .004), and integration with psychosocial rehabilitation (cognition: χ^2^_1_, 5.66; functioning: χ^2^_1_, 12.08) were crucial ingredients of efficacy. Patients with fewer years of education (global cognition: coefficient, −0.055 [95% CI, −0.103 to −0.006]; *P* = .03; global functioning: coefficient, −0.061 [95% CI, −0.112 to −0.011]; *P* = .02), lower premorbid IQ (global functioning: coefficient, −0.013 [−0.025 to −0.001]; *P* = .04), and higher baseline symptom severity (global cognition: coefficient, 0.006 [95% CI, 0.002 to 0.010]; *P* = .005) emerged as optimal candidates.

**Conclusions and Relevance:**

These findings show that CR is an evidence-based intervention that should be included consistently into clinical guidelines for the treatment of individuals with schizophrenia and implemented more widely in clinical practice.

## Key Points

**Question:**

What is the effectiveness of cognitive remediation on cognition and functioning in people diagnosed with schizophrenia, and what are its active ingredients?

**Findings:**

In this systematic review and meta-analysis, cognitive remediation was confirmed as effective on both cognitive and functional outcomes and potentially useful for all patients with schizophrenia, even those most severely affected. An active and trained therapist, structured development of cognitive strategies, and integration with rehabilitation were crucial ingredients of efficacy.

**Meaning:**

This analysis demonstrates that cognitive remediation is an evidence-based intervention, which should be recommended and implemented more widely in the standard treatment of schizophrenia.

## Teaser

This meta-analysis of randomized clinical trials gives a comprehensive update on cognitive remediation effectiveness for cognition and functioning in patients with schizophrenia and analyzes the core components of efficacy.

## Introduction

Schizophrenia is a debilitating mental disorder often associated with poor functional outcomes.^1,2,3^ Cognitive deficits represent a core feature,^4^ are evident from an early age,^5,6^ and are strongly associated with functional impairment.^2,3,7,8^ These problems constitute one of the main limiting factors for recovery in the context of psychiatric treatment and rehabilitation.^9,10,11,12^

Cognitive remediation (CR) for schizophrenia, as defined by the Cognitive Remediation Experts Workshop (2010), is a behavioral training–based intervention that aims to improve cognitive processes with the goal of durability and generalization.^13^ Since its conception, different interventions based on these principles have been developed and implemented with considerable differences in structure, setting, and schedule.

A solid body of evidence attests to the efficacy of CR: the most comprehensive meta-analysis^14^ included the results of 40 studies and reported modest to moderate effect sizes (ES) on cognitive and functional measures. Many new trials investigating various CR programs have now been published, and more than 20 meta-analyses have focused on specific topics, such as the efficacy in patients with a diagnosis of schizophrenia with recent onset,^15^ in inpatient settings,^16^ on negative symptoms,^17^ or of specific types of interventions (ie, computer based^18^ or integrated with psychiatric rehabilitation^19^). Despite this wealth of evidence, there is still reluctance to implement CR into services, even though some guidance already suggests providing such treatment.^20,21,22^

In some studies, significant cognitive improvement did not emerge^23,24,25^ or the improvements were not translated into better psychosocial functioning.^26^ These negative findings suggest the existence of factors not yet fully investigated that affect CR benefits.^27^ In particular, some clinically relevant issues remain to be addressed, such as the optimal CR delivery in real-world settings, the active ingredients of CR,^27^ and the moderators of response.^13,28,29,30,31^

A recent expert consensus^13^ identified 4 core elements: the presence of an active and trained therapist, repeated practice of cognitive exercises, structured development of cognitive strategies, and use of techniques to improve the transfer of cognitive gains to the real world. However, to date, to our knowledge, no systematic review has explicitly and quantitatively explored the outcomes of use of these ingredients.

Available evidence on which patients best respond or are resistant to CR does not provide conclusive results.^27,28,30,31^ Identifying the role of potential moderating variables could have a positive outcome by both allowing a more tailored approach and optimizing the use of resources in health care delivery settings.^32^

All these issues have limited recommendations for using CR in national and international guidelines for the treatment of schizophrenia.^33,34^ To our knowledge, the last comprehensive meta-analysis including all different CR modalities dates to 2011.^14^ An update, conducted with an inclusive and rigorous approach, could provide definite answers to the open issues in the field and support future recommendations on CR implementation into clinical practice.

This study investigates CR effectiveness on cognitive performance systematically and its generalizability to functional outcomes, providing an updated and inclusive overview of the different randomized clinical trials. It includes an analysis of the proposed active treatment components,^13^ as well as the influence of other treatment-associated and participant-associated factors.

## Methods

A systematic review and meta-analysis were conducted following the Preferred Reported Items for Systematic Review and Meta-analysis (PRISMA) guidelines.^35^ A detailed methodology is in eAppendix 1 in the Supplement.

### Search Strategy and Selection Criteria

The reference list of Wykes et al^14^ was screened against eligibility criteria. Then, a systematic literature search was conducted on 3 electronic databases (PubMed, Scopus, and PsycInfo) from January 2011 to February 2020, using the following terms: (“cognitive” or “cognit*”) AND (“training” or “remediation” or “rehabilitation” or “enhancement”) AND (“schizophrenia” or “psychosis”) AND (“random” or “randomized control trial” or “clinical trial”). Emerging meta-analyses or reviews and reference lists of included articles were also hand searched, and Google Scholar was manually inspected.

At least 2 independent reviewers (from a group of 3 authors: A.C., G.N., and C.A.) assessed the reports and extracted data; disagreements were resolved by a third author (among A.V., S.B., and G.D.). Only articles in English published in peer-reviewed journals were considered.

We adopted a comprehensive approach, so eligibility criteria were purposely broad. Inclusion focused on randomized clinical trials comparing CR with any control condition other than CR, among patients diagnosed with schizophrenia spectrum disorders who constituted at least 70% of study sample, independent of setting. The CR interventions, either applied as stand-alone treatments or combined with other adequately controlled psychosocial interventions, had to fulfill the standard Experts Workshop definition for CR (2010), with no restrictions in terms of duration, intensity, and mode of delivery. To account for the heterogeneity of treatment as usual (TAU) and separate interventions simply controlling for nonspecific aspects, 4 comparison groups were identified: (1) TAU (eg, drug treatment/case management, waiting lists, TAU with no description provided), (2) active TAU (including multidisciplinary rehabilitative programs), (3) active nonspecific interventions controlling for nonspecific aspects and matched with CR for duration and schedule (eg, social stimulation, leisure activities, computer activities), and (4) active evidence-based interventions^36,37^ specifically implemented for comparison purposes.

### Quality Assessment

Included studies were assessed by 2 independent reviewers (among A.C., G.N., and C.A.) using the Clinical Trials Assessment Measure.^38^ A cutoff score of 65 of 100 points^39^ was used to compare adequate vs inadequate methodology. The most meaningful quality items were also treated as dichotomous variables.

### Outcome Measures

Primary outcomes were changes in global cognitive performance and overall functioning from baseline to posttreatment; these outcomes were also subsequently investigated through metaregressions and subgroup and sensitivity analyses. Additional outcomes were changes in specific cognitive domains and symptom severity.

For cognitive performance, data on all objective and validated cognitive tasks were extracted and classified into the 7 categories derived from the National Institute of Mental Health–Measurement and Treatment Research to Improve Cognition in Schizophrenia Neurocognition Committee^40^ (eTable 1 in the Supplement). Since no general consensus exists regarding the attribution of neuropsychological tools to cognitive domains, we referred to previous articles.^5,14^ If agreement could not be reached even after discussion between 5 reviewers (A.V., S.B., A.C., G.N., and G.D.), the scales were not used. Subjective rating scales for cognition and instruments modified by study authors or not appropriately validated were not extracted. Following Wykes et al,^14^ domain-specific ES values were calculated as means of available ES values of individual measures and then combined to obtain a composite ES.^41^

For functioning, available and validated measures were extracted for each study. Self-rated, caregiver-rated, and investigator-rated instruments were all eligible, independent from the area of functioning. Both direct and indirect measures of functioning, such as functional capacity, living and social skills, and quality of life, were included to obtain a comprehensive picture.

When studies reported multiple rating instruments for symptoms, only 1 scale per study was chosen, prioritizing the Positive and Negative Syndrome Scale (PANSS),^42^ or, if not available, the Brief Psychiatric Rating Scale,^43^ following the Cochrane Collaboration^44^ recommendations and adopted in high-quality meta-analyses.^45^ When studies only reported other instruments, the most representative tool was identified based on the hypothesized frequency of use. Positive and negative symptoms were analyzed separately; an ES for global symptoms was derived only if full-scale total scores were available.

For studies with multiple treatment arms, each eligible comparison was considered separately. The issue of dependent ES was addressed in sensitivity analyses restricted to 1 ES per study.^46,47^

### Meta-analytic Procedures

For each outcome measure, Cohen *d* and SEs were calculated.^48,49^ If raw group means, *z* scores, and SDs were not available, they were extracted using WebPlotDigitizer version 4.2 (Rohatgi), or group × time interaction *F* values were used.^50^ Missing data were treated using an available-case approach; data resulting from intention-to-treat approaches were preferred. A random-effects approach was applied. Meta-analyses were performed using Review Manager version 5.3 (The Cochrane Collaboration), while metaregressions using Comprehensive Meta-Analysis version 3.0 (Biostat).

### Moderator Effects

Variables associated with methodology of included studies, characteristics of included interventions, and study participants were investigated: publication year, overall methodological quality, presence of blinding, use of intention-to-treat approaches, comparison category, inclusion of diagnoses besides schizophrenia, the 4 core elements of CR,^13^ format of delivery, computer use, treatment duration (in weeks) and intensity (in sessions per week and hours per week), participants’ age, sex (expressed as percentages of female participants), years of education, premorbid IQ, age at onset, duration of illness, baseline treatment dosage (chlorpromazine equivalents), and baseline symptom severity.

### Certainty of the Evidence

Confidence in pooled results for primary outcomes was further evaluated through sensitivity analyses (eAppendix 1 in the Supplement). Risk of publication bias was assessed by visual inspection of funnel plots and a statistical test of asymmetry (Egger test).^51^ In case of significant asymmetry, adjustment of effect estimates was investigated with the trim-and-fill method, using both a random-random and a fixed-random effects model.^52,53^ Other determinants of quality of evidence (consistency, precision, and directness) were explored according to experts’ recommendation.^54^

## Results

**Figure 1.**
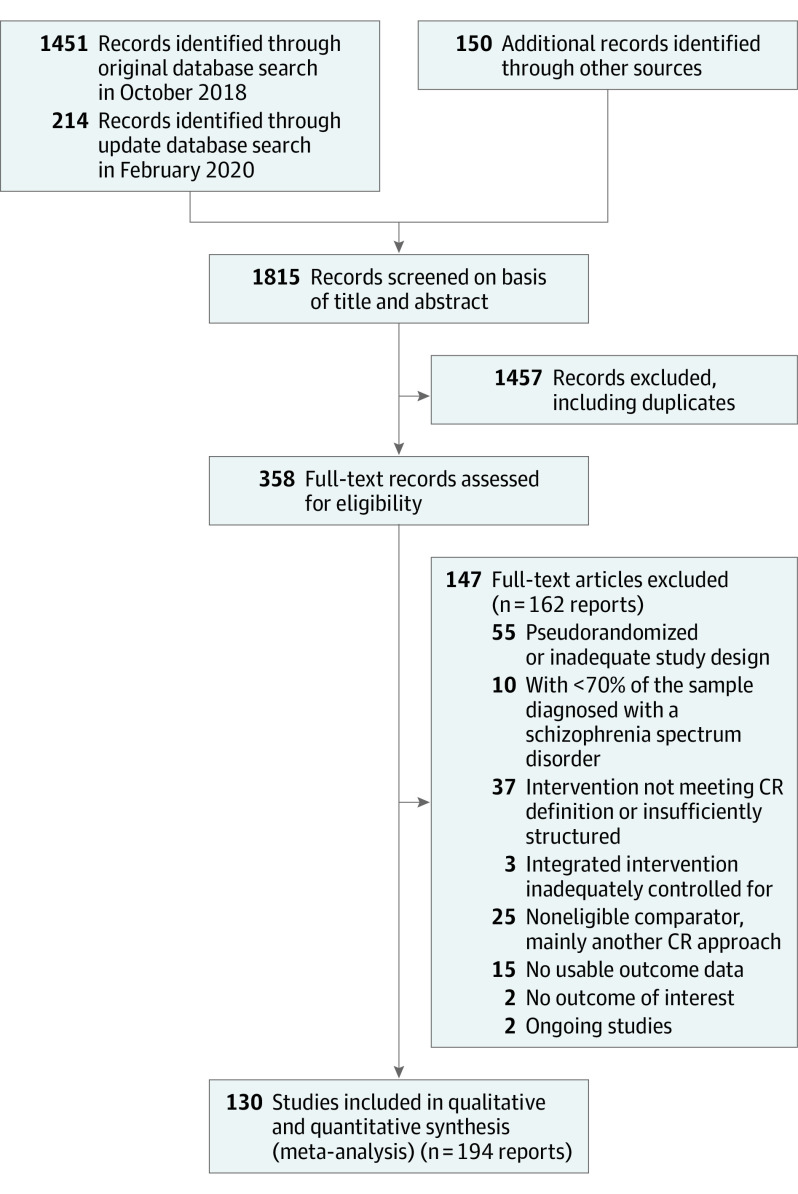
PRISMA Study Flow Diagram CR indicates cognitive remediation.

Figure 1 shows study selection procedure. One-hundred thirty studies, reporting 146 CR-control comparisons with a total of 8851 participants, were included; 2 ongoing studies were identified (eAppendix 2 in the Supplement).

### Included Studies

Fifty-seven studies were conducted in Europe, 38 in the US, 22 in Asia, 4 in Canada, 4 in Middle East countries, 3 in Australia, and 2 in Brazil. Studies drew their samples from different outpatient and inpatient services; 3 trials^55,56,57^ were conducted in forensic settings. Descriptive data are shown in Table 1.^58^

**Table 1.** Descriptive Characteristics of 130 Included Studies Reporting Data on 143 Interventions and 146 Intervention-Control Comparisons

Characteristic	Total studies included, No.	Studies with characteristic, No. (%)
Design		
Single-center trial	130	89 (68.5)
Multicenter trial	130	41 (31.5)
Setting		
Outpatients	130	79 (60.8)
Inpatients	130	35 (26.9)
Both	130	16 (12.3)
Sample size, mean (SD) [range]	128	68.6 (40.4) [10-311]
Methodological quality		
Total Clinical Trials Assessment Measure score, mean (SD) [range], points	130	63.1 (14.1) [26-92]
Trials with ≥65 points	130	67 (51.5)
Trials with <65 points	130	63 (48.5)
Blinding		
Open trial	130	52 (40.0)
Blind trial with unclear details	130	55 (42.3)
Blind trial providing details	130	23 (17.7)
Adequate dealing with missing data^a^	130	59 (45.4)
Attrition rate, mean (SD) [range], %	121	13.7 (11.6) [0-47.8]
Including only individuals with schizophrenia	130	59 (45.4)
Providing payment to included individuals		
Payment for participation/training sessions	130	20 (15.4)
Payment for assessments only	130	5 (3.9)
Comparison category		
Treatment as usual	146	50 (34.3)
Active treatment as usual	146	22 (15.1)
Nonspecific control	146	45 (30.8)
Active intervention	146	29 (19.9)
Patient and illness characteristics		
Age, mean (SD) [range], y	130	36.7 (7.0) [15.3-51.3]
Female participants, mean (SD) [range], %	123	32.0 (13.9) [0-75]
Education, mean (SD), [range], y	92	11.9 (1.3) [8.8-14.9]
Premorbid IQ, mean (SD) [range]	55	95.6 (7.9) [74.8-111.4]
Age at onset, mean (SD) [range], y	87	23.3 (2.6) [13.4-28.8]
Duration of illness, mean (SD) [range], y	88	13.8 (6.3) [0.7-29.7]
Baseline therapy dose, mean (SD) [range], chlorpromazine equivalents	64	562.2 (278.7) [182.5-1609.7]
Baseline PANSS score, mean (SD) [range]	78	68.7 (15.7) [41.9-118.4]
Baseline symptom severity^b^		
Mild	78	46 (59.0)
Moderate	78	19 (24.4)
Marked	78	11 (14.1)
Severe	78	2 (2.6)
Treatment characteristics		
Treatment duration, mean (SD) [range], wk	143	15.2 (14.3) [3-104]
Treatment intensity, mean (SD) [range]	143	
Sessions/wk	136	2.6 (1.3) [0.5-7.8]
h/wk	134	2.6 (1.5) [0.4-10]
Format of delivery		
Individual sessions	143	69 (48.3)
Group sessions	143	53 (37.1)
Both individual and group sessions	143	21 (14.7)
Method of delivery		
Computer assisted	143	60 (42.0)
Pencil and paper	143	43 (30.1)
Both methods	143	40 (28.0)
Core elements included, No.^c^		
1. Active and trained therapist	143	115 (80.4)
2. Practice of cognitive exercises for ≥20 h	143	105 (73.4)
3. Development of cognitive strategies	143	104 (72.7)
4. Facilitation of transfer to everyday functioning	143	102 (71.3)
4.* Adjunctive psychiatric rehabilitation	143	39 (27.3)
Interventions fulfilling elements 1, 2, 3, and 4*	143	29 (20.3)

Abbreviation: PANSS, Positive and Negative Syndrome Scale.

^a^ For example, use of intention-to-treat analysis.

^b^ Conversion of PANSS to Clinical Global Impression Scale score according to Leucht et al.^58^

^c^ Per Bowie et al.^13^

Half the studies were methodologically adequate (Clinical Trials Assessment Measure score ≥65 points); trial quality evolved over time, with recent studies showing better ratings (Spearman ρ = 0.288; *P* = .001) and more adequate assessment methods (Spearman ρ = 0.283; *P* = .001). Overall trial quality was not associated with setting or intervention characteristics.

Included populations were representative of patients with schizophrenia, using mental health services, at different stages of illness and clinical conditions. The mean (range) treatment duration was 15.2 (3-104) weeks. Core elements of CR^13^ were well represented: active and trained therapists (115 [80.4%]), repeated practice of cognitive exercises (105 [73.4%]; most of the remaining studies included it but lasted <20 hours), structured development of cognitive strategies (104 [72.7%]), and facilitated transfer to everyday functioning (102 [71.3%]). In 39 interventions (27.3%), transfer was provided through integration of CR with psychiatric rehabilitation (eTable 2 in the Supplement). Cognitive remediation was either compared with TAU (50 [34.3%]), active TAU (22 [15.1%]), active nonspecific interventions (45 [30.8%]), or active evidence-based interventions specifically used for the studies (29 [19.9%]) (eTable 3 in the Supplement).

### Effectiveness of CR

**Figure 2.**
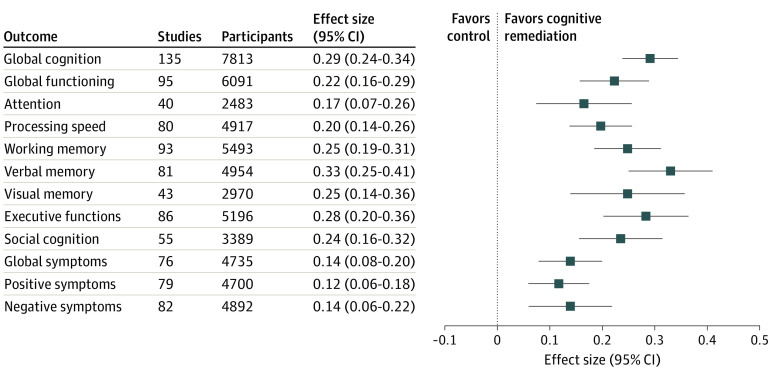
Effects of Cognitive Remediation Summary of effect sizes (Cohen *d*) of primary outcomes (global cognition and global functioning) and additional outcomes of the meta-analysis.

A small to moderate effect of CR was observed on primary outcomes (global cognition: *d*, 0.29 [95% CI, 0.24-0.34]; *P* < .001; 135 comparisons; global functioning: *d*, 0.22 [95% CI, 0.16-0.29]; *P* < .001; 95 comparisons; eFigures 1 and 2 in the Supplement). In both analyses, overall heterogeneity was low (global cognition: *I*^2^, 24%; global functioning: *I*^2^, 37%) and was reduced substantially by removing outliers (eFigures 1 and 2 in the Supplement). For most single cognitive domains, the outcome was significant and in the same range (*d* ≥0.20). The observed result was smaller for change in symptom severity, although significant (global symptoms: *d*, 0.14 [95% CI, 0.08-0.20]; *P* < .001; 76 comparisons) (Figure 2).

### Active Ingredients of CR Affecting CR Benefits: Core Elements and Treatment Characteristics That Moderate Outcomes 

Interventions including an active and trained therapist were more effective on cognition (χ^2^_1_, 4.14; *P* = .04) and functioning (χ^2^_1_, 4.26; *P* = .04) than those that did not include an active and trained therapist. The same was true for the structured development of cognitive strategies (cognition: χ^2^_1_, 9.34; *P* = .002; functioning: χ^2^_1_, 8.12; *P* = .004).

Techniques facilitating transfer of cognitive skills into real-world settings did not have a meaningful influence on outcomes. However, an additional analysis, performed post hoc, considered only integration with psychiatric rehabilitation as a transfer technique. This demonstrated a significant influence on functioning (χ^2^_1_, 9.11; *P* = .003).

Interventions including all core elements^13^ (considering psychiatric rehabilitation as the optimal transfer technique) had highly significant associations with both main outcomes (global cognition: χ^2^_1_, 5.66; *P* = .02; global functioning: χ^2^_1_, 12.08; *P* < .001; eFigures 3 and 4 in the Supplement). This finding remained when including only methodologically adequate studies (eFigures 5 and 6 in the Supplement).

Analyzing other potential treatment-associated moderators (Table 2) only found treatment duration to have a significant influence on functional improvement (coefficient, 0.006 [95% CI, 0.002-0.010]; *P* = .006) (eFigure 7 in the Supplement). There was no association with format and method of delivery.

**Table 2.** Effects of Moderators on Cognitive and Functional Outcomes

Moderator	Global cognition				Global functioning			
	No. of studies	Statistic type	Statistic value (95% CI)	*P* value	No. of studies	Statistic type	Statistic value (95% CI)	*P* value
Study characteristics								
Publication year	135	Coefficient	−0.005 (−0.014 to 0.004)	.29	95	Coefficient	−0.011 (−0.024 to 0.001)	.08
CTAM score	135	Coefficient	−0.005 (−0.009 to −0.001)	.02	95	Coefficient	−0.007 (−0.012 to 0.002)	.005
Methodological quality								
CTAM score ≥65	73	Cohen *d*	0.26 (0.19 to 0.32)	NA	61	Cohen *d*	0.18 (0.10 to 0.25)	NA
CTAM score <65	62	Cohen *d*	0.34 (0.25 to 0.43)	NA	34	Cohen *d*	0.32 (0.19 to 0.45)	NA
Test for subgroup differences	NA	χ^2^_1_	2.35	.13	NA	χ^2^_1_	3.60	.06
Blinding								
Open trials	53	Cohen *d*	0.36 (0.26 to 0.45)	NA	29	Cohen *d*	0.38 (0.23 to 0.53)	NA
Blind trials	82	Cohen *d*	0.26 (0.20 to 0.32)	NA	66	Cohen *d*	0.17 (0.10 to 0.24)	NA
Test for subgroup differences	NA	χ^2^_1_	2.88	.09	NA	χ^2^_1_	6.15	.01
Use of intention-to-treat principle								
Intention-to-treat analysis	66	Cohen *d*	0.31 (0.24 to 0.39)	NA	42	Cohen *d*	0.15 (0.07 to 0.23)	NA
Completer/per-protocol analysis	61	Cohen *d*	0.28 (0.20 to 0.36)	NA	47	Cohen *d*	0.25 (0.17 to 0.33)	NA
Test for subgroup differences	NA	χ^2^_1_	0.38	.54	NA	χ^2^_1_	2.94	.09
Attrition rate (%)	126	Coefficient	−0.002 (−0.007 to 0.003)	.49	89	Coefficient	−0.001 (−0.007 to 0.005)	.72
Sample size (No. randomized)	135	Coefficient	−0.001 (−0.002 to 0.0003)	.16	95	Coefficient	−0.001 (−0.003 to 0.000)	.04
Comparison category								
Treatment as usual	46	Cohen *d*	0.28 (0.19 to 0.36)	NA	30	Cohen *d*	0.23 (0.10 to 0.35)	NA
Active treatment as usual	21	Cohen *d*	0.43 (0.26 to 0.60)	NA	9	Cohen *d*	0.29 (0.08 to 0.50)	NA
Active nonspecific interventions	42	Cohen *d*	0.24 (0.17 to 0.32)	NA	35	Cohen *d*	0.21 (0.12 to 0.31)	NA
Active evidence-based interventions	26	Cohen *d*	0.32 (0.17 to 0.46)	NA	21	Cohen *d*	0.21 (0.05 to 0.37)	NA
Test for subgroup differences	NA	χ^2^_3_	4.16	.25	NA	χ^2^_3_	0.49	.92
Individuals with schizophrenia included								
Only individuals with schizophrenia	60	Cohen *d*	0.34 (0.25 to 0.42)	NA	38	Cohen *d*	0.28 (0.18 to 0.39)	NA
Including other diagnoses	75	Cohen *d*	0.25 (0.19 to 0.32)	NA	57	Cohen *d*	0.19 (0.10 to 0.27)	NA
Test for subgroup differences	NA	χ^2^_1_	2.33	.13	NA	χ^2^_1_	1.98	.16
**Treatment characteristics**								
Active and trained therapist (core element 1)								
Present	107	Cohen *d*	0.32 (0.26 to 0.38)	NA	78	Cohen *d*	0.25 (0.17 to 0.32)	NA
Absent	28	Cohen *d*	0.19 (0.08 to 0.30)	NA	17	Cohen *d*	0.10 (−0.03 to 0.22)	NA
Test for subgroup differences	NA	χ^2^_1_	4.14	.04	NA	χ^2^_1_	4.26	.04
Repeated practice of cognitive exercises (core element 2)								
Present	102	Cohen *d*	0.29 (0.24 to 0.34)	NA	80	Cohen *d*	0.23 (0.16 to 0.30)	NA
Absent	33	Cohen *d*	0.30 (0.15 to 0.45)	NA	15	Cohen *d*	0.19 (−0.04 to 0.42)	NA
Test for subgroup differences	NA	χ^2^_1_	0.01	.92	NA	χ^2^_1_	0.08	.77
Development of cognitive strategies (core element 3)								
Present	96	Cohen *d*	0.34 (0.27 to 0.40)	NA	71	Cohen *d*	0.27 (0.18 to 0.35)	NA
Absent	39	Cohen *d*	0.18 (0.10 to 0.26)	NA	24	Cohen *d*	0.09 (−0.01 to 0.18)	NA
Test for subgroup differences	NA	χ^2^_1_	9.34	.002		χ^2^_1_	8.12	.004
Techniques of transfer to the real world (core element 4)								
Present	94	Cohen *d*	0.30 (0.24 to 0.36)	NA	66	Cohen *d*	0.24 (0.16 to 0.31)	NA
Absent	41	Cohen *d*	0.26 (0.15 to 0.38)	NA	29	Cohen *d*	0.20 (0.07 to 0.33)	NA
Test for subgroup differences	NA	χ^2^_1_	0.32	.57	NA	χ^2^_1_	0.23	.63
Integration with rehabilitation (core element 4*^a^)								
Present	37	Cohen *d*	0.37 (0.27 to 0.47)	NA	26	Cohen *d*	0.38 (0.26 to 0.50)	NA
Absent	98	Cohen *d*	0.26 (0.20 to 0.32)	NA	69	Cohen *d*	0.16 (0.08 to 0.23)	NA
Test for subgroup differences	NA	χ^2^_1_	3.39	.07	NA	χ^2^_1_	9.11	.003
Interventions including core elements 1, 2, 3, and 4*^a^								
All core elements	28	Cohen *d*	0.40 (0.30 to 0.49)	NA	20	Cohen *d*	0.43 (0.30 to 0.57)	NA
Not all core elements	107	Cohen *d*	0.26 (0.20 to 0.32)	NA	75	Cohen *d*	0.16 (0.09 to 0.23)	NA
Test for subgroup differences	NA	χ^2^_1_	5.66	.02	NA	χ^2^_1_	12.08	<.001
Treatment duration, wk	135	Coefficient	−0.002 (−0.004 to 0.003)	.85	95	Coefficient	0.006 (0.002 to 0.010)	.006
Treatment intensity, sessions/wk	130	Coefficient	0.013 (−0.029 to 0.055)	.55	92	Coefficient	−0.014 (−0.067 to 0.040)	.62
Treatment intensity, h/wk	128	Coefficient	0.008 (−0.029 to 0.046)	.67	92	Coefficient	−0.040 (−0.093 to 0.014)	.15
Format of delivery								
Individual format	66	Cohen *d*	0.28 (0.20 to 0.35)	NA	49	Cohen *d*	0.20 (0.10 to 0.30)	NA
Group format	49	Cohen *d*	0.27 (0.19 to 0.35)	NA	33	Cohen *d*	0.20 (0.11 to 0.28)	NA
Both components	20	Cohen *d*	0.39 (0.23 to 0.54)	NA	13	Cohen *d*	0.33 (0.13 to 0.54)	NA
Test for subgroup differences	NA	χ^2^_2_	1.89	.39	NA	χ^2^_2_	1.52	.47
Computer presentation								
Computerized intervention	61	Cohen *d*	0.25 (0.18 to 0.31)	NA	41	Cohen *d*	0.18 (0.08 to 0.29)	<.001
Pencil-and-paper intervention	39	Cohen *d*	0.39 (0.27 to 0.52)	NA	26	Cohen *d*	0.32 (0.21 to 0.42)	<.001
Both methods of delivery	35	Cohen *d*	0.26 (0.16 to 0.36)	NA	25	Cohen *d*	0.20 (0.07 to 0.33)	.002
Test for subgroup differences	NA	χ^2^_2_	4.15	.13	NA	χ^2^_2_	3.48	.18
Patient and illness characteristics								
Age, y	135	Coefficient	−0.003 (−0.011 to 0.004)	.40	95	Coefficient	0.000 (−0.008 to 0.009)	.91
Female, %	126	Coefficient	0.000 (−0.005 to 0.005)	.97	88	Coefficient	0.004 (−0.002 to 0.010)	.18
Education, y	98	Coefficient	−0.055 (−0.103 to −0.006)	.03	73	Coefficient	−0.061 (−0.112 to −0.011)	.02
Premorbid IQ	60	Coefficient	0.005 (−0.005 to 0.013)	.39	39	Coefficient	−0.013 (−0.025 to −0.001)	.04
Age at onset, y	92	Coefficient	−0.019 (−0.043 to 0.0005)	.12	69	Coefficient	−0.003 (−0.039 to 0.033)	.86
Duration of illness, y	93	Coefficient	0.001 (−0.009 to 0.011)	.90	70	Coefficient	−0.001 (−0.012 to 0.011)	.92
Baseline treatment dose, chlorpromazine equivalents	58	Coefficient	0.000 (−0.0003 to 0.0003)	.93	54	Coefficient	0.000 (−0.0003 to 0.0004)	.77
Baseline PANSS score	85	Coefficient	0.006 (0.002 to 0.010)	.005	68	Coefficient	0.004 (−0.0002 to 0.009)	.06

Abbreviations: CTAM, Clinical Trials Assessment Measure; NA, not applicable; PANSS, Positive and Negative Syndrome Scale.

^a^ Core element 4* is adjunctive psychiatric rehabilitation.

### Likely Ideal Candidates for CR and Patient-Associated Moderators

Fewer years of education (global cognition: coefficient, −0.055 [95% CI, −0.103 to −0.006]; *P* = .03; global functioning: coefficient, −0.061 [95% CI, −0.112 to −0.011]; *P* = .02), lower premorbid IQ (global functioning: coefficient, −0.013 [−0.025 to −0.001]; *P* = .04), and higher baseline symptom severity (global cognition: coefficient, 0.006 [95% CI, 0.002 to 0.010]; *P* = .005) were associated with larger improvements on main outcomes. No other clinical variables emerged as significant moderators (Table 2). No correlations among significant participant-associated and illness-associated moderators emerged, except for premorbid IQ and education (Spearman ρ = 0.302; *P* = .049).

### Level of Confidence in the Evidence

Some methodological issues (overall methodological quality, use of blinding, and study sample size) influenced the treatment effect on the primary outcomes, mainly functioning (Table 2). Sensitivity analyses did not change the observed results, including those restricted to 1 ES per study, which did not produce relevant variations in confidence intervals of global effect estimates or observed heterogeneity.

No evidence of publication bias emerged for cognition. A slight asymmetry of funnel plot was found for functioning, with some studies missing on the left side of the graph. The trim-and-fill method produced no changes in the effect estimate with a random-random model; some adjustment was observed with the fixed-random model (eFigures 8 and 9 in the Supplement).

A noteworthy risk of indirectness of outcome was identified for functioning; the studies investigated different functioning areas in different proportions and used different assessment tools, often relying on indirect measures. However, pooled estimates of effects for both primary outcomes were precise and consistent.

## Discussion

### Effectiveness of CR

This meta-analysis represents the most recent and comprehensive evaluation of CR effects in people diagnosed with schizophrenia. It included a very large number of studies and more than 8000 participants and found an overall positive impact of CR on global cognition and functioning, confirming the effectiveness of CR previously reported.^14^ The global effectiveness of CR was already known and attested^13,27,28^; however, confirming this finding with an inclusive and rigorous update strengthens the notion that CR represents a valid treatment.

The observed benefits for cognition and functioning were slightly smaller than those reported in the Wykes et al meta-analysis.^14^ This was to be expected because of the inclusion of many more recent and methodologically rigorous articles, and it can be also explained by the heterogeneous samples of patients and interventions included. The observation of positive ES values in the context of such a diverse sample represents a clinically relevant strength of CR interventions. The global influence on symptoms was less substantial, and this was also in line with previous reports.^14^

These results were robust, especially for global cognition, in which no influence was observed for differences in study setting or control conditions. Some factors (sample size, blinding, and statistical handling of missing data) did seem to affect functioning. Overall, the influence of study quality on the observed results was judged not to be substantial because studies with better methodological quality tend to show smaller ES values.^44,59^

The absence of differences associated with control conditions is an unexpected finding. For cognitive outcomes, a possible explanation is that CR specifically targets cognitive performance, while other evidence-based interventions are not tailored for this outcome. For functional outcomes, the wide heterogeneity in the administered interventions, care setting, and sample characteristics could have limited the observation of a differential effect, especially because the various control conditions were not directly compared.

### Active Ingredients and Treatment Characteristics That Moderate Outcomes 

The proposed core elements^13^ had a relevant impact. Notably, the presence of an active and trained therapist had a significant influence on cognitive and functional outcomes. This has been a debated issue for CR experts and suggests that unsupervised programs would not be likely to contribute to recovery outcomes of importance.^60^ The structured development of novel cognitive strategies produced a significant benefit on both outcomes.

No significant benefit was observed for intensive repeated practice. A possible explanation is that almost all the analyzed interventions included this element, but some did not reach the proposed threshold for duration and intensity.^13^ There is currently insufficient information to define the optimal schedule required to observe a differential outcome, and this topic represents an important focus for future studies.

The implementation of structured psychiatric rehabilitation was analyzed separately as a technique for transferring cognitive skills to functioning, showing a significant influence. Rehabilitation interventions are aimed at promoting patients’ recovery; our results suggest a complementary association between CR and psychiatric rehabilitation, in that adding CR boosts the rehabilitation outcomes, and pairing CR with psychiatric rehabilitation may also increase CR generalizability to real-world functioning. The relevance of this finding should clearly be weighed against the context of care and the availability of resources to use multiple interventions.

Interventions with all 4 core elements^13^ produced a significantly larger benefit in both primary outcomes. This remained robust even when restricted to studies with adequate methods. Therefore, the characteristics that have been theorized to represent fundamental elements of CR do indeed have an association with its effectiveness.

Treatment duration was directly associated only with functional gain. Cognitive remediation format and mode of delivery had no significant difference. The available data therefore do not allow the choice of any CR technique as superior; instead, the optimization of treatment effectiveness appears to be mediated by the implementation of the essential CR components.

### Ideal Candidates for CR and Patient Characteristics That Moderate Outcomes

Our results revealed a significant role of education, premorbid IQ, and symptom severity, indicating that patients who are clinically compromised are valid candidates for CR.^61,62^ The available literature has not provided high-quality replicated conclusions, even from a systematic perspective^28,30,31^; some studies conversely suggest that better baseline cognition and/or clinical status may be associated with better outcomes,^63,64,65,66^ while others show no significant benefits.^67,68,69^ The picture emerging from this meta-analysis is, however, supported by several studies.^70,71,72,73,74,75^ A recent meta-analysis^16^ reported consistent effectiveness of CR interventions among inpatients, who usually present a more severe clinical condition. Since improvement was the measure analyzed, it is possible that a better outcome was observed in patients in a worse clinical condition because they present larger room for improvement. This hypothesis is supported by trials conducted on patients who are clinically compromised^61,76^ or comparing baseline impairment subgroups.^77^ However, patients with better clinical status might also respond; previous evidence shows that a better baseline cognitive profile might be associated with a greater chance of cognitive performance normalization after CR.^78^

Age and duration of illness did not emerge as significant moderators, again in contrast to previous evidence.^68,79,80^ Some studies have proposed CR as an early intervention strategy^81,82^; our findings suggest that CR could be offered to all participants, regardless of age and history of illness.^14^

Reasons for the differences between the results of the meta-analyses and those of single studies could include the fact that most studies report positive correlations, concealing the potential role of negative studies.^27,28,30,31^ Furthermore, there are discrepancies in outcome definitions, with some studies focusing on the dimension of cognitive improvement and others on the chance of normalization of cognitive performance.^78^ Another critical issue is the possibility of intercorrelations among different variables.^27,28,30,31^

### Strengths

A strength is the large number and representativeness of included studies which allowed more nuanced analyses. Methodological quality was investigated in sensitivity analyses, confirming the robustness of ES estimates. The main outcomes showed high consistency and precision.

### Limitations

Although significant asymmetry in the funnel plot emerged for global functioning, it is possible that this observation might be better explained by clinical and methodological heterogeneity between included studies rather than by publication bias. The restriction to studies published in the English language could represent a source of publication bias; however, the influence of this element is often described as small.^83,84^

We did not examine the CR cost-effectiveness or durability of improvements, both potentially valuable for services. However, there were genuine benefits for service users in domains they think are important.

Although our findings support longer treatment duration producing greater functional gain, we cannot specify an optimal treatment duration. This requires further research.

Finally, the interactions between moderators could not be analyzed with the model adopted. Integrating treatment-associated and patient-associated variables in models would allow us to assess the unique role of each moderator. Moreover, some aspects of the present work, such as the role of different types of controls and a direct comparison of different CR interventions, could be better investigated with a network meta-analysis. This represents an interesting perspective for future studies.

## Conclusions

Cognitive remediation produces meaningful benefits in cognition and functioning in this analysis, so implementation for people with a diagnosis of schizophrenia should be recommended in clinical practice. The theorized core elements of CR are vital for its effectiveness, in that interventions that include them all could produce greater benefits and mental health services that intend to introduce CR into routine practice should ensure that these core ingredients are included. Notably, the transfer of cognitive gains into real-world settings is better obtained by integrating CR with a structured psychiatric rehabilitation. The effectiveness of CR does not appear to be overly influenced by patient-associated characteristics, suggesting that it is a viable option for most individuals with a diagnosis of schizophrenia. Cognitive remediation implementation should also be suggested in services for patients who are clinically compromised, because these participants appear to present substantial room for improvement.

These findings represent a solid foundation for including CR consistently in national and international treatment recommendations. It is an evidence-based treatment, with the potential to be introduced as an element of standard care rather than an optional intervention targeting selected individuals. Because pharmacological treatment exerts limited effects on cognitive deficits and clinical remission does not necessarily result in functional recovery, widespread implementation of CR could be a game-changer for achieving the patient’s personal recovery goals.

## Notes

Supplement. **eAppendix 1.** Materials and methods - Detailed section eReferences. **eTable 1.** List of rating scales and corresponding cognitive domains **eAppendix 2.** References of included and ongoing studies and of inspected reviews **eTable 2.** Cognitive remediation programs adopted in included studies **eTable 3.** Summary of individual characteristics of included studies **eFigure 1.** Forest plot for the effects of cognitive remediation on global cognition **eFigure 2.** Forest plot for the effects of cognitive remediation on global functioning **eFigure 3.** Subgroup analysis for the effects of interventions including all core elements of cognitive remediation (global cognition) **eFigure 4.** Subgroup analysis for the effects of interventions including all core elements of cognitive remediation (global functioning) **eFigure 5.** Sensitivity analysis: subgroup analysis in eFigure 3. including only methodologically adequate studies (global cognition) **eFigure 6.** Sensitivity analysis: subgroup analysis in eFigure 4. including only methodologically adequate studies (global functioning) **eFigure 7.** Meta-analytic scatter plot for global functioning and duration of cognitive remediation programs **eFigure 8.** Analysis of publication bias: funnel plot for global cognition **eFigure 9.** Analysis of publication bias: funnel plot for global functioning
