# Supplementary material for: Effectiveness, Core Elements, and Moderators of Response of Cognitive Remediation for Schizophrenia: A Systematic Review and Meta-analysis of Randomized Clinical Trials
Source: JAMA Psychiatry. 2021 Apr 20;78(8):1–12. doi: 10.1001/jamapsychiatry.2021.0620 (PMC8058696; doi:10.1001/jamapsychiatry.2021.0620)
Supplement: Supplement. — eAppendix 1. Materials and methods - Detailed section eReferences. eTable 1. List of rating scales and corresponding cognitive domains eAppendix 2. References of included and ongoing studies and of inspected reviews eTable 2. Cognitive remediation programs adopted in included studies eTable 3. Summary of individual characteristics of included studies eFigure 1. Forest plot for the effects of cognitive remediation on global cognition eFigure 2. Forest plot for the effects of cognitive remediation on global functioning eFigure 3. Subgroup analysis for the effects of interventions including all core elements of cognitive remediation (global cognition) eFigure 4. Subgroup analysis for the effects of interventions including all core elements of cognitive remediation (global functioning) eFigure 5. Sensitivity analysis: subgroup analysis in eFigure 3. including only methodologically adequate studies (global cognition) eFigure 6. Sensitivity analysis: subgroup analysis in eFigure 4. including only methodologically adequate studies (global functioning) eFigure 7. Meta-analytic scatter plot for global functioning and duration of cognitive remediation programs eFigure 8. Analysis of publication bias: funnel plot for global cognition eFigure 9. Analysis of publication bias: funnel plot for global functioning [file jamapsychiatry-e210620-s001.pdf]

Supplemental Online Content

Vita A, Barlati S, Ceraso A, et al. Effectiveness, core elements, and moderators of response of cognitive remediation for schizophrenia: a systematic review and meta-analysis of randomized clinical trials. *JAMA Psychiatry*. Published online April 20, 2021. doi:10.1001/jamapsychiatry.2021.0620

eAppendix 1. Materials and methods - Detailed section

eReferences.

eTable 1. List of rating scales and corresponding cognitive domains

eAppendix 2. References of included and ongoing studies and of inspected reviews

eTable 2. Cognitive remediation programs adopted in included studies

eTable 3. Summary of individual characteristics of included studies

eFigure 1. Forest plot for the effects of cognitive remediation on global cognition

eFigure 2. Forest plot for the effects of cognitive remediation on global functioning

eFigure 3. Subgroup analysis for the effects of interventions including all core elements of cognitive remediation (global cognition)

eFigure 4. Subgroup analysis for the effects of interventions including all core elements of cognitive remediation (global functioning)

eFigure 5. Sensitivity analysis: subgroup analysis in eFigure 3. including only methodologically adequate studies (global cognition)

eFigure 6. Sensitivity analysis: subgroup analysis in eFigure 4. including only methodologically adequate studies (global functioning)

eFigure 7. Meta-analytic scatter plot for global functioning and duration of cognitive remediation programs

eFigure 8. Analysis of publication bias: funnel plot for global cognition

eFigure 9. Analysis of publication bias: funnel plot for global functioning

This supplemental material has been provided by the aSuthors to give readers additional information about their work.

© 2021 Vita et al. *JAMA Psychiatry.*

eAppendix 1. Materials and Methods – Detailed section.

Search strategy and selection criteria

As an initial step, the reference list of Wykes et al.[1] was screened against eligibility criteria. Then, a systematic literature search was conducted on three electronic databases (PubMed, Scopus, and PsycINFO; from January 2011 to October 2018, updated in February 2020) using the following strategy: ("cognitive" or "cognit*") AND ("training" or "remediation" or "rehabilitation" or "enhancement") AND ("schizophrenia" or "psychosis") AND ("random" or "randomized control trial" or "clinical trial"). Emerging meta-analyses/reviews, and reference lists of papers fulfilling inclusion criteria were also hand-searched. Finally, keywords of the search strategy were used to conduct an additional search of Google Scholar.

All reports were assessed by at least two independent reviewers, firstly based on title and abstract, then through full-text inspection; the same reviewers independently extracted data; disagreements were resolved with the assistance of a third author. Only papers available in English and published in peer-reviewed journals were considered.

Eligibility criteria were purposely broad, in line with the aim of adopting a comprehensive approach. Inclusion focused on RCTs comparing CR to any control condition other than CR, among patients diagnosed with schizophrenia spectrum disorders (any diagnostic criteria), independently of setting. Included studies were required to present outcomes at baseline and early post-treatment phase.

Feasibility trials were retrieved, if appropriately randomized. Pseudo- and quasi-randomized trials were excluded; in case of crossover trials, only data up to the point of crossover were used.

Eligible populations were required to include at least 70% of patients diagnosed with a schizophrenia spectrum disorder; alternatively, specific outcome data on diagnostic subgroups had to be available.

Included studies had to investigate a structured intervention fulfilling the standard Experts Workshop definition for CR (Florence, Italy, 2010), either applied as a stand-alone treatment or combined with other psychosocial interventions (if adequately controlled for), with no restrictions in terms of duration, intensity and mode of delivery. Studies evaluating selected modules of manualized CR programs (i.e. pre-specified set of exercises that can be administered independently, usually covering a specific content or cognitive domain, and meeting by themselves the CR definition) were eligible. Suitable interventions specifically targeting

© 2021 Vita et al. *JAMA Psychiatry.*

metacognition were also included, since several recent CR programs tend to focus on metacognitive aspects rather than on task practice only.[2]

Particular attention was devoted to the definition of comparison groups, an overlooked but crucial aspect for the understanding of measured outcomes of psychosocial interventions.[3] Both the "absolute" effect of CR and the "relative" effect of specific intervention ingredients were of interest for the present work. Rather than simply classifying comparison conditions as "passive" or "active", they were specifically categorized into four groups: 1. Treatment as usual (TAU: drug treatment/case management only, waiting lists, insufficient details); 2. Active TAU (including multidisciplinary rehabilitative programs); 3. Active non-specific interventions (controlling for non-specific aspects and matched with CR for duration and schedule, e.g. social stimulation, leisure activities, computer activities); 4. Active evidence-based interventions[4,5] specifically implemented for comparison purpose. This categorization was designed to account for the heterogeneity of the concept of TAU and to separate interventions simply controlling for non-specific aspects of attending a new treatment (e.g. time spent with a therapist, computer use).

Quality assessment

Included studies were assessed by two independent reviewers using the Clinical Trials Assessment Measure (CTAM),[6] a tool designed to examine trials of cognitive behavioral therapy[6] but already adopted to evaluate trials of CR[1] or other psychosocial interventions.[7] The CTAM total score (0-100) provides an overall display of study quality according to six domains: sample recruitment, allocation, outcome assessment, comparison groups, analysis, and treatment description. A cut-off of 65 points[8] was used to identify and compare adequate *versus* inadequate methodology. The most meaningful CTAM items (i.e. description of random sequence generation, use and description of blinding, handling of missing data, testing treatment fidelity) were also treated as dichotomous variables, to account for the potential extent of bias due to the different weight provided by each domain to the total score. Study authors were not contacted to confirm CTAM scoring, but quality ratings were compared with those of other review groups reported in available publications.[1,9,10] Studies where a significant risk of reporting bias was identified were separately noted, as

© 2021 Vita et al. *JAMA Psychiatry.*

selective reporting is not represented in the CTAM, but is known to produce overestimates of intervention effects.[11,12]

Outcome measures

Primary outcomes were changes in global cognitive performance and overall functioning from baseline to post-treatment; these outcomes were also subsequently investigated through meta-regressions, subgroup, and sensitivity analyses. Additional outcomes were changes in specific cognitive domains and in symptom severity.

For cognitive performance, data on all objective and validated cognitive tasks were extracted and classified into the seven categories derived from the NIMH-MATRICS Neurocognition Committee:[13] attention/vigilance, speed of processing, working memory, verbal learning and memory, visual learning and memory, reasoning and problem solving, social cognition. Since no general consensus exists regarding the attribution of specific neuropsychological tools to cognitive domains, we referred to previous publications.[1,14] If a clear agreement could not be reached even after collegial discussion between at least five reviewers, the scales were not used. Subjective rating scales for cognition and instruments modified by study authors or not appropriately validated in a previously published study were not extracted. The list of scales and corresponding cognitive domains is reported in Supplementary Table 1.

For each study, domain-specific effect sizes were calculated by averaging available effect sizes of the individual measures referring to that specific domain; then, a composite global cognition effect size was calculated by averaging the available domain-specific effect sizes. The aggregation of effect sizes within each study before meta-analyzing the results over studies represents an often-employed approach to the problem of dependent effect sizes.[15] Wykes et al.[1] also adopted this strategy and demonstrated that combining measures did not influence the estimate of global effects; since covariance tends to increase the composite effect size and to underestimate standard error,[16] this method can be viewed as conservative.

© 2021 Vita et al. *JAMA Psychiatry.*

For functioning outcomes, all available and validated measures were extracted for each study. Self-, caregiver- and investigator-rated instruments were all eligible, independently from the area of functioning (e.g. daily life, education, work, interpersonal relationships). Both direct and indirect measures of functioning, such as functional capacity, and living and social skills, were included to obtain a comprehensive picture. Accordingly, quality of life measures were also included.[17]

When studies used multiple rating instruments for symptoms, only one scale per study was chosen, prioritizing the Positive And Negative Syndrome Scale (PANSS),[18] or, if not available, the Brief Psychiatric Rating Scale,[19] following the procedures suggested by the Cochrane Collaboration[20] and adopted in previous high-quality meta-analyses.[21] If multiple rating instruments were applied but neither of these scales was included, the most representative tool was identified based on the hypothesized frequency of use. Positive and negative symptoms were analyzed separately; an effect size for global symptoms was derived only if full-scale total scores were available.

For studies with multiple treatment arms, each comparison of interest was considered separately, if based on appropriate allocation procedure. As including multiple effect sizes per study (dependent effect sizes) could produce biased statistical inferences, this issue was addressed in sensitivity analyses restricted to one effect size per study, both randomly and choosing the most substantial comparison.[22,23]

Effect-size calculation

For each outcome measure, Cohen's d was calculated according to the formula by Carlson & Schmidt,[24] which allows to control for baseline values and heterogeneity of variance.[25] The standard errors of the effect sizes were calculated using the formula recommended by Cooper et al.[16] If the raw group means, Z-scores and standard deviations were not available, they were extracted using WebPlotDigitizer version 4.2 (Rohatgi, San Francisco, CA, USA, 2019) or group x time interaction F values or t values were used.[26] Where standard deviations were missing, they were calculated from available data (standard error or p values for between-group differences).[20] Scores of each scale were adapted so that a higher score reflected a better performance;

© 2021 Vita et al. *JAMA Psychiatry.*

in this way a positive Cohen's d value was indicative of a positive treatment effect. Missing data were treated using an available-case approach; data resulting from intention-to-treat were preferred when reported.

Meta-analytic procedure

All meta-analyses used a random effects approach. Statistical heterogeneity was investigated through visual inspection of forest plots and assessment of χ^2-tests and I^2 statistic. Potential reasons for heterogeneity were then explored through subgroup analyses for categorical variables and restricted-maximum-likelihood-random-effect meta-regressions for continuous variables. All the meta-analyses were performed using Review Manager, version 5.3 (The Cochrane Collaboration, Copenhagen, DK, 2014), except for the meta-regressions, conducted using Comprehensive Meta-Analysis version 3.0 (Biostat, Englewood, NJ, USA, 2013). Descriptive statistics and analyses were performed using SPSS version 14.0 (SPSS Inc., Chicago, IL, USA, 2005).

Moderator effects

Potential moderators were investigated for primary outcomes. Firstly, study-related moderators were investigated: publication year, overall methodological quality (based on total CTAM score and on the 65-points cut-off), blinding of study condition/outcome assessment, use of intention-to-treat, comparison category, inclusion of diagnoses other than schizophrenia. Then, characteristics of included treatments were explored, primarily the four core elements of CR identified in the most recent expert consensus[27], along with individual or group format of delivery, computer use, treatment duration (weeks) and intensity (sessions/week and hours/week). Finally, patient- and illness-related moderators were investigated: age, gender (% female subjects), years of education, premorbid IQ, age of onset, duration of illness, baseline treatment dose (chlorpromazine equivalents - CPZeq) and baseline symptom severity. For medication, when CPZeq was not explicitly reported, the daily dose was derived from available data.[28] Baseline symptom severity was expressed as total PANSS score; eventual Brief Psychiatric Rating Scale scores were converted to PANSS.[29] Interconnections between moderators emerging as significant were explored through inferential statistics to check for potential collinearity.

© 2021 Vita et al. *JAMA Psychiatry.*

Certainty of the evidence

Confidence in pooled results for primary outcomes was further evaluated through sensitivity analyses: use of a fixed effect model, exclusion of clear outliers, inclusion of only one ES per study, exclusion of studies with inadequate methodology (according to CTAM) or insufficient details on allocation, providing only completers data, evaluating interventions designed for trial purpose, not testing intervention quality/fidelity to manual. Publication bias was assessed by visual inspection of funnel plots and statistical test of asymmetry (Egger's test).[30] In case of significant asymmetry, adjustment of effect estimates was investigated with the trim-and-fill method using both a random-random and a fixed-random effects model.[31,32] Finally, other potential determinants of quality of evidence (i.e. consistency, precision and directness) were explored, according to experts' recommendation.[33]

eReferences

1. Wykes T, Huddy V, Cellard C, McGurk SR, Czobor P. A meta-analysis of cognitive remediation for schizophrenia: methodology and effect sizes. *Am J Psychiatry*. 2011;168(5):472-485. doi:10.1176/appi.ajp.2010.10060855

2. Cella M, Reeder C, Wykes T. Cognitive remediation in schizophrenia—now it is really getting personal. *Curr Opin Behav Sci*. 2015;4:147-151. doi:10.1016/j.cobeha.2015.05.005

3. Radhakrishnan R, Kiluk BD, Tsai J. A Meta-analytic Review of Non-specific Effects in Randomized Controlled Trials of Cognitive Remediation for Schizophrenia. *Psychiatr Q*. 2016;87(1):57-62. doi:10.1007/s11126-015-9362-6

4. Firth J, Stubbs B, Rosenbaum S, et al. Aerobic Exercise Improves Cognitive Functioning in People With Schizophrenia: A Systematic Review and Meta-Analysis. *Schizophr Bull*. 2017;43(3):546-556. doi:10.1093/schbul/sbw115

5. Mueser KT, Deavers F, Penn DL, Cassisi JE. Psychosocial treatments for schizophrenia. *Annu Rev Clin Psychol*. 2013;9:465-497. doi:10.1146/annurev-clinpsy-050212-185620

6. Tarrier N, Wykes T. Is there evidence that cognitive behaviour therapy is an effective treatment for schizophrenia? A cautious or cautionary tale? *Behav Res Ther*. 2004;42(12):1377-1401. doi:10.1016/j.brat.2004.06.020

7. Lobban F, Postlethwaite A, Glentworth D, et al. A systematic review of randomised controlled trials of interventions reporting outcomes for relatives of people with psychosis. *Clin Psychol Rev*. 2013;33(3):372-382. doi:10.1016/j.cpr.2012.12.004

8. Wykes T, Steel C, Everitt B, Tarrier N. Cognitive behavior therapy for schizophrenia: effect sizes, clinical models, and methodological rigor. *Schizophr Bull*. 2008;34(3):523-537. doi:10.1093/schbul/sbm114

9. Seccomandi B, Tsapekos D, Newbery K, Wykes T, Cella M. A systematic review of moderators of cognitive remediation response for people with schizophrenia. *Schizophr Res Cogn*. 2020;19:100160. doi:10.1016/j.scog.2019.100160

10. Grant N, Lawrence M, Preti A, Wykes T, Cella M. Social cognition interventions for people with schizophrenia: a systematic review focussing on methodological quality and intervention modality. *Clin Psychol Rev*. 2017;56:55-64. doi:10.1016/j.cpr.2017.06.001

© 2021 Vita et al. *JAMA Psychiatry.*

11. Furukawa TA, Watanabe N, Omori IM, Montori VM, Guyatt GH. Association Between Unreported Outcomes and Effect Size Estimates in Cochrane Meta-analyses. *JAMA*. 2007;297(5):465-470. doi:10.1001/jama.297.5.468-b

12. Guyatt GH, Oxman AD, Montori V, et al. GRADE guidelines: 5. Rating the quality of evidence--publication bias. *J Clin Epidemiol*. 2011;64(12):1277-1282. doi:10.1016/j.jclinepi.2011.01.011

13. Nuechterlein KH, Barch DM, Gold JM, Goldberg TE, Green MF, Heaton RK. Identification of separable cognitive factors in schizophrenia. *Schizophr Res*. 2004;72(1):29-39. doi:10.1016/j.schres.2004.09.007

14. Fusar-Poli P, Deste G, Smieskova R, et al. Cognitive functioning in prodromal psychosis: a meta-analysis. *Arch Gen Psychiatry*. 2012;69(6):562-571. doi:10.1001/archgenpsychiatry.2011.1592

15. Van den Noortgate W, López-López JA, Marín-Martínez F, Sánchez-Meca J. Meta-analysis of multiple outcomes: a multilevel approach. *Behav Res Methods*. 2015;47(4):1274-1294. doi:10.3758/s13428-014-0527-2

16. Cooper H, Hedges LV, Valentine JC. *The Handbook of Research Synthesis and Meta-Analysis*. Russell Sage Foundation; 2019.

17. Nevarez-Flores AG, Sanderson K, Breslin M, Carr VJ, Morgan VA, Neil AL. Systematic review of global functioning and quality of life in people with psychotic disorders. *Epidemiol Psychiatr Sci*. 2019;28(1):31-44. doi:10.1017/S2045796018000549

18. Kay SR, Fiszbein A, Opler LA. The positive and negative syndrome scale (PANSS) for schizophrenia. *Schizophr Bull*. 1987;13(2):261-276.

19. Overall JE, Gorham DR. The Brief Psychiatric Rating Scale. *Psychol Rep*. 1962;10(3):799-812. doi:10.2466/pr0.1962.10.3.799

20. Higgins JP, Green S. *Cochrane Handbook for Systematic Reviews of Interventions*. Vol 4. John Wiley & Sons; 2011.

21. Leucht S, Leucht C, Huhn M, et al. Sixty Years of Placebo-Controlled Antipsychotic Drug Trials in Acute Schizophrenia: Systematic Review, Bayesian Meta-Analysis, and Meta-Regression of Efficacy Predictors. *Am J Psychiatry*. 2017;174(10):927-942. doi:10.1176/appi.ajp.2017.16121358

22. Van den Noortgate W, López-López JA, Marín-Martínez F, Sánchez-Meca J. Three-level meta-analysis of dependent effect sizes. *Behav Res Methods*. 2013;45(2):576-594. doi:10.3758/s13428-012-0261-6

23. Rücker G, Cates CJ, Schwarzer G. Methods for including information from multi-arm trials in pairwise meta-analysis. *Res Synth Methods*. 2017;8(4):392-403. doi:https://doi.org/10.1002/jrsm.1259

24. Carlson KD, Schmidt FL. Impact of experimental design on effect size: Findings from the research literature on training. *J Appl Psychol*. 1999;84(6):851-862. doi:10.1037/0021-9010.84.6.851

25. Morris SB. Estimating Effect Sizes From Pretest-Posttest-Control Group Designs. *Organ Res Methods*. 2008;11(2):364-386. doi:10.1177/1094428106291059

26. Thalheimer W, Cook S. How to calculate effect sizes from published research: A simplified methodology.2009.

27. Bowie CR, Bell MD, Fiszdon JM, et al. Cognitive remediation for schizophrenia: An expert working group white paper on core techniques. *Schizophr Res*. Published online November 4, 2019. doi:10.1016/j.schres.2019.10.047

28. Woods SW. Chlorpromazine equivalent doses for the newer atypical antipsychotics. *J Clin Psychiatry*. 2003;64(6):663-667. doi:10.4088/jcp.v64n0607

29. Leucht S, Kane JM, Kissling W, Hamann J, Etschel E, Engel RR. What does the PANSS mean? *Schizophr Res*. 2005;79(2-3):231-238. doi:10.1016/j.schres.2005.04.008

30. Egger M, Davey Smith G, Schneider M, Minder C. Bias in meta-analysis detected by a simple, graphical test. *BMJ*. 1997;315(7109):629-634. doi:10.1136/bmj.315.7109.629

31. Duval S, Tweedie R. Trim and fill: A simple funnel-plot-based method of testing and adjusting for publication bias in meta-analysis. *Biometrics*. 2000;56(2):455-463. doi:10.1111/j.0006-341x.2000.00455.x

© 2021 Vita et al. *JAMA Psychiatry.*

32. Peters JL, Sutton AJ, Jones DR, Abrams KR, Rushton L. Performance of the trim and fill method in the presence of publication bias and between-study heterogeneity. *Stat Med*. 2007;26(25):4544-4562. doi:https://doi.org/10.1002/sim.2889

33. Schünemann H. *The GRADE Handbook*. Cochrane Collaboration; 2013.

© 2021 Vita et al. *JAMA Psychiatry.*

eTable 1. List of rating scales and corresponding cognitive domains.

<u>**Attention/Vigilance**</u>	<u>**Verbal Learning and Memory**</u>	<u>**Social Cognition**</u>	<u>**Social Functioning**</u>
Continuous Performance Tests (CPT)<br>Sustained Attention Test (SAT)<br>Test of Sustained and Selective Attention (TASS)<br>Tests for Attentional Performance (TAP) Vigilance<br>Attention Stress Test<br>Scanning Test<br>Digit Vigilance Test<br>Go/No-go<br>Triads Test<br>Span of Apprehension tests<br>Letter Cancellation Test<br>Embedded Stimulus tests<br>Backward Masking Test (BMT)<br>COGLAB Preattentional Processing<br>WMS Information<br><br><u>**Speed of Processing**</u><br>Reaction Time tests<br>Tests for Attentional Performance (TAP) Alertness<br>Trail Making Test, Part A (TMT-A)<br>Stroop Test, Color and Word conditions<br>WAIS Digit Symbol<br>Digit Symbol Substitution Test<br>Symbol Digit Modalities Test (SDMT)<br>BACS Symbol Coding<br>BACS Token Motor<br>Finger Tapping Test (FTT)<br>Tactile Performance<br>Verbal Fluency tests (Category and Letter)<br>D-KEFS Symbol Search<br>D-KEFS Color Naming	Hopkins Verbal Learning Test (HVLT)<br>Rey Auditory Verbal Learning Test (RAVLT)<br>California Verbal Learning Test (CVLT)<br>WMS Logical Memory<br>WMS Memory Passages<br>WMS Verbal Paired Associates<br>BACS Verbal Memory<br>LPAD Word Memory<br>NCSE Memory<br>Hong Kong List Learning Test (HKLLT)<br>Other Word List recall tests<br>Free and Cued Selective Reminding Test (FCSRT)<br>Prose Recall tests<br>Denman Neuropsychological Memory Test<br>Rivermead Behavioral Memory Test (RBMT)<br><br><u>**Visual Learning and Memory**</u><br>Brief Visuospatial Memory Test (BVMT)<br>Benton Visual Retention Test (BVRT)<br>Rey Osterrieth Complex Figure<br>Rey Visual Design Learning Test (RVDLT)<br>LPAD Complex Figure<br>WMS Visual Reproduction<br>WMS Memory for Faces<br>Face Memory Test (FMT)<br>Kimura Recurring Figures Test<br>Denman Neuropsychological Memory Test<br>Rivermead Behavioral Memory Test (RBMT)	Mayer-Salovey-Caruso Emotional Intelligence Test (MSCEIT)<br>Bell Lysaker Emotion Recognition Task (BLERT)<br>Facial Emotion Identification Test (FEIT)<br>Face Emotion Discrimination Task (FEDT)<br>Facial Emotion Recognition Task (TREF)<br>Penn Emotion Recognition Test (ER-40)<br>Ekman 60 Faces Test<br>Vienna Emotion Recognition Test (VERT-K)<br>Geneva Emotion Recognition Test (GERT)<br>Pictures of Facial Affect (PFA)<br>Emotion Recognition Questionnaire (EMOREC)<br>Emotion in Biological Motion (EmoBio)<br>fMRI Emotion Recognition tasks<br>The Awareness of Social Inference Task (TASIT)<br>Reading the Mind in the Eyes Test (RMET)<br>Reading Mind in the Voices (RMV)<br>Hinting Task<br>Happè Stories<br>Movie for the Assessment of Social Cognition (MASC)<br>Social Behavior Sequencing Task (SBST)<br>Social Cognition Screening Questionnaire – Theory of Mind<br>Profile of Non-Verbal Sensitivity (PONS)<br>Schema Component Sequencing Task (SCST)<br>Situational Feature Recognition Test (SFRT)<br>Script Test<br>Mach IV Scale<br>Relationships Across Domains (RAD)<br>Faux Pas Detection Test<br>Davos Assessment of Cognitive Biases (DACOBS) – Social Cognition Problems<br>Self-Report Cognitive Biases (CBQp) Emotion Based Reasoning<br>Ambiguous Intentions Hostility Questionnaire (AIHQ)<br>Internal Personal and Situational Attribution Questionnaire (IPSAQ)<br>Attributional Style Questionnaire (ASQ)<br>Self-Report Cognitive Biases (CBQp) – Intentionalizing<br>Empathic Accuracy Test<br>Unexpected Outcome Test (UOT)<br>Questionnaire of Cognitive and Affective Empathy	UCSD Performance-Based Skills Assessment (UPSA)<br>Independent Living Scales, Problem Solving Factor (ILS-PS)<br>Medication Management Ability Assessment<br>Micro-Module Learning Test (MMLT)<br>Social Skills Performance Assessment (SSPA)<br>Independent Living Skills Survey (ILSS)<br>Life Skills Profile (LSP)<br>Maryland Assessment of Social Competence<br>Work Behavior Inventory (WBI)<br>Assessment of Interpersonal Problem Solving Skills (AIPSS)<br>UCLA Module Specific Skills Assessment<br>Global Assessment of Functioning (GAF; GAF-f)<br>Global Assessment Scale (GAS)<br>Children Global Assessment Scale (C-GAS)<br>Social Functioning Scale (SFS)<br>Personal and Social Performance (PSP)<br>Specific Levels of Functioning (SLOF)<br>Role Functioning Scale (RFS)<br>WHO Disability Assessment Scale (WHO-DAS)<br>Interview for the Assessment of Disability (AD)<br>Social and Occupational Functioning Assessment Scale (SOFAS)<br>Health of the Nation Outcome Scale (HoNOS)<br>Employability Appraisal Scale (EAS)<br>Life Assessment Scale for the Mentally Ill (LASMI)<br>Social Adjustment Scale (SAS)<br>Social Behavior Scale (SBS)<br>Major Role Adjustment Inventory (MRAI)<br>VADO Personal and Social Functioning Scale<br>Vineland Adaptive Behavior Scale (VABS)<br>Performance Potential Inventory (PPI)<br>Time use survey<br>Nurses Observation Scale for Inpatient Evaluation (NOSIE)<br>Scale of Social Skills of chronic schizophrenia inpatients (SSSI)<br>Occupational Self-Assessment (OSA)<br>Behavior Rating Inventory of Executive Functions (BRIEF-A)<br>Informant- report and Self-report<br>Heinrichs Carpenter Quality of Life Scale (QLS)<br>WHO Quality of Life (WHOQOL; WHOQOL-BREF)<br>EUROHIS Quality of Life (EUROHIS-QOL)<br>Self-Report Quality of Life in schizophrenia (SQol)

© 2021 Vita et al. *JAMA Psychiatry.*

			Personal Well-Being Index (PWI)<br>Quality of Life Interview (QoLI)<br>Quality of Life Scale (QOLS)
**Working Memory**<br>WAIS, WISC or WMS Digit Span<br>Digit Span Distractibility Test<br>BACS Digit Sequencing<br>Other Digit Span tasks<br>WAIS or WMS Letter-Number Sequencing<br>Auditory Number Sequencing (ANS)<br>Maintenance and Manipulation Task<br>WAIS Arithmetic<br>Other arithmetic tasks<br>WMS Mental Control<br>Trail Making Test, Part B (TMT-B)<br>Self Ordered Pointing Task (SOPT)<br>Visual Conditional Associative Learning (VCALT)<br>Sentence Span tests<br>N-back<br>Auditory Consonant Trigrams (ACT)<br>WMS Visual/Spatial Span<br>Dual Span<br>CANTAB Spatial Working Memory<br>Test for Attentional Performance (TAP) Working Memory	**Reasoning and Problem Solving**<br>Wisconsin Card Sorting Test (WCST) or COGLAB Card Sorting Test - Categories achieved and Perseverative Errors<br>Delis Kaplan Executive Function System (D-KEFS) (all problem solving, planning, abstract reasoning, switching and response inhibition subtests)<br>BACS Tower of London<br>Tower of Hanoi<br>WAIS or WISC Picture Arrangement/Block Design<br>WAIS Matrix Reasoning<br>NCSE Construction<br>Mazes test<br>Trail Making Test, Part B-A (TMT B-A)<br>Stroop Test, Interference condition<br>Response Inhibition Test<br>Penn Conditional Exclusion Test<br>Strategic Target Detection Test (STDT)<br>WAIS Similarities<br>NCSE Reasoning<br>Booklet Category Test<br>Six Elements Test<br>Labyrinth Test<br>Proverb Interpretation tasks<br>Behavioral Assessment of the Dysexecutive Syndrome (BADS)<br>Ecological Shopping Test	**Cognitive measures of multiple domains (included when available)**<br>Global Cognition composite scores<br>Mini Mental State Examination (MMSE)<br><br>**Cognitive measures lacking consensus (excluded)**<br>Cognitive Style tests<br>Hayling Sentence Completion Task<br>WMS Orientation<br>Purdue Pegboard<br>Auditory Frequency Discrimination Test<br>Subjective rating scales to assess cognition<br>Emotion Processing tests: Reaction time, Confidence in responses<br>Other measures of "confidence in decision"<br><br>**Cognitive measures not considered sensitive to change (excluded)**<br>WAIS Comprehension<br>WAIS Vocabulary	**Symptoms**<br>Positive and Negative Syndrome Scale (PANSS) Global score<br>Brief Psychiatric Rating Scale (BPRS) Global score<br><br>**Positive Symptoms**<br>PANSS Positive Scale or Factor Score<br>BPRS Positive Symptoms factors<br>Scale for the Assessment of Positive Symptoms (SAPS)<br>Psychotic Symptom Rating Scale (PSYRATS)<br>Inkblot test<br><br>**Negative Symptoms**<br>PANSS Negative Scale or Factor<br>BPRS Negative Symptoms factors<br>Scale for the Assessment of Negative Symptoms (SANS)

BACS: Brief Assessment of Cognition in Schizophrenia; CANTAB: Cambridge Neuropsychological Test Automated Battery; fMRI: Functional Magnetic Resonance Imaging; LPAD: Learning Potential Assessment Device; NCSE: Neurobehavioral Cognitive Status Examination; WAIS: Wechsler Adult Intelligence Scale; WISC: Wechsler Intelligence Scale for Children; WMS: Wechsler Memory Scale.

© 2021 Vita et al. *JAMA Psychiatry.*

eAppendix 2.

References of included studies

1. Aghotor J, Pfueller U, Moritz S, Weisbrod M, Roesch-Ely D. Metacognitive training for patients with schizophrenia (MCT): feasibility and preliminary evidence for its efficacy. J Behav Ther Exp Psychiatry 2010; 41: 207–11.
2. Ahmed AO, Hunter KM, Goodrum NM, et al. A randomized study of cognitive remediation for forensic and mental health patients with schizophrenia. J Psychiatr Res 2015; 68: 8–18.
3. Ahuir M, Cabezas Á, Miñano MJ, et al. Improvement in cognitive biases after group psychoeducation and metacognitive training in recent-onset psychosis: A randomized crossover clinical trial. Psychiatry Res 2018; 270: 720–3.
4. Aloi M, de Filippis R, Grosso Lavalle F, et al. Effectiveness of integrated psychological therapy on clinical, neuropsychological, emotional and functional outcome in schizophrenia: a RCT study. J Ment Health Abingdon Engl 2018; : 1–8.
5. Au DWH, Tsang HWH, So WWY, et al. Effects of integrated supported employment plus cognitive remediation training for people with schizophrenia and schizoaffective disorders. Schizophr Res 2015; 166: 297–303.
6. Bell M, Bryson G, Greig T, Corcoran C, Wexler BE. Neurocognitive enhancement therapy with work therapy: effects on neuropsychological test performance. Arch Gen Psychiatry 2001; 58: 763–8.
7. Bellucci DM, Glaberman K, Haslam N. Computer-assisted cognitive rehabilitation reduces negative symptoms in the severely mentally ill. Schizophr Res 2003; 59: 225–32.
8. Bowie CR, McGurk SR, Mausbach B, Patterson TL, Harvey PD. Combined cognitive remediation and functional skills training for schizophrenia: effects on cognition, functional competence, and real-world behavior. Am J Psychiatry 2012; 169: 710–8.
9. Briki M, Monnin J, Haffen E, et al. Metacognitive training for schizophrenia: a multicentre randomised controlled trial. Schizophr Res 2014; 157: 99–106.
10. Bryce SD, Rossell SL, Lee SJ, et al. Neurocognitive and Self-efficacy Benefits of Cognitive Remediation in Schizophrenia: A Randomized Controlled Trial. J Int Neuropsychol Soc JINS 2018; 24: 549–62.
11. Burda PC, Starkey TW, Dominguez F, Vera V. Computer-assisted cognitive rehabilitation of chronic psychiatric inpatients. Comput Hum Behav 1994; 10: 359–68.
12. Byrne LK, Peng D, McCabe M, et al. Does practice make perfect? Results from a Chinese feasibility study of cognitive remediation in schizophrenia. Neuropsychol Rehabil 2013; 23: 580–96.
13. Cassetta BD, Tomfohr-Madsen LM, Goghari VM. A randomized controlled trial of working memory and processing speed training in schizophrenia. Psychol Med 2019; 49: 2009–19.
14. Cavallaro R, Anselmetti S, Poletti S, et al. Computer-aided neurocognitive remediation as an enhancing strategy for schizophrenia rehabilitation. Psychiatry Res 2009; 169: 191–6.
15. Choi K-H, Kwon J-H. Social cognition enhancement training for schizophrenia: a preliminary randomized controlled trial. Community Ment Health J 2006; 42: 177–87.
16. Choi K-H, Kang J, Kim S-M, et al. Cognitive Remediation in Middle-Aged or Older Inpatients with Chronic Schizophrenia: A Randomized Controlled Trial in Korea. Front Psychol 2017; 8: 2364.
17. d'Amato T, Bation R, Cochet A, et al. A randomized, controlled trial of computer-assisted cognitive remediation for schizophrenia. Schizophr Res 2011; 125: 284–90.
18. D'Souza DC, Radhakrishnan R, Perry E, et al. Feasibility, Safety, and Efficacy of the Combination of *D*-Serine and Computerized Cognitive Retraining in Schizophrenia: An International Collaborative Pilot Study. Neuropsychopharmacology 2013; 38: 492–503.
19. Dickinson D, Tenhula W, Morris S, et al. A randomized, controlled trial of computer-assisted cognitive remediation for schizophrenia. Am J Psychiatry 2010; 167: 170–80.
20. Donohoe G, Dillon R, Hargreaves A, et al. Effectiveness of a low support, remotely accessible, cognitive remediation training programme for chronic psychosis: cognitive, functional and cortical outcomes from a single blind randomised controlled trial. Psychol Med 2018; 48: 751–64.
21. Drake RJ, Day CJ, Picucci R, et al. A naturalistic, randomized, controlled trial combining cognitive remediation with cognitive-behavioural therapy after first-episode non-affective psychosis. Psychol Med 2014; 44: 1889–99.
22. Eack SM, Greenwald DP, Hogarty SS, et al. Cognitive enhancement therapy for early-course schizophrenia: effects of a two-year randomized controlled trial. Psychiatr Serv Wash DC 2009; 60: 1468–76.

© 2021 Vita et al. *JAMA Psychiatry.*

23. Fan F, Zou Y, Tan Y, Hong LE, Tan S. Computerized cognitive remediation therapy effects on resting state brain activity and cognition in schizophrenia. Sci Rep 2017; 7. DOI:10.1038/s41598-017-04829-9.
24. Farreny A, Aguado J, Ochoa S, et al. REPYFLEC cognitive remediation group training in schizophrenia: Looking for an integrative approach. Schizophr Res 2012; 142: 137–44.
25. Favrod J, Rexhaj S, Bardy S, et al. Sustained antipsychotic effect of metacognitive training in psychosis: a randomized-controlled study. Eur Psychiatry J Assoc Eur Psychiatr 2014; 29: 275–81.
26. Fernandez-Gonzalo S, Turon M, Jodar M, et al. A new computerized cognitive and social cognition training specifically designed for patients with schizophrenia/schizoaffective disorder in early stages of illness: A pilot study. Psychiatry Res 2015; 228: 501–9.
27. Fisher M, Loewy R, Carter C, et al. Neuroplasticity-Based Auditory Training Via Laptop Computer Improves Cognition in Young Individuals With Recent Onset Schizophrenia. Schizophr Bull 2015; 41: 250–8.
28. Fisher M, Mellon SH, Wolkowitz O, Vinogradov S. Neuroscience-informed Auditory Training in Schizophrenia: A Final Report of the Effects on Cognition and Serum Brain-Derived Neurotrophic Factor. Schizophr Res Cogn 2016; 3: 1–7.
29. Fiszdon JM, Choi KH, Bell MD, Choi J, Silverstein SM. Cognitive remediation for individuals with psychosis: efficacy and mechanisms of treatment effects. Psychol Med 2016; 46: 3275–89.
30. Galderisi S, Piegari G, Mucci A, et al. Social skills and neurocognitive individualized training in schizophrenia: comparison with structured leisure activities. Eur Arch Psychiatry Clin Neurosci 2010; 260: 305–15.
31. García S, Fuentes I, Ruíz JC, Gallach E, Roder V. Application of the IPT in a Spanish sample: Evaluation of the 'social perception subprogramme'. Int J Psychol Psychol Ther 2003; 3: 299–310.
32. García-Fernández L, Cabot-Ivorra N, Rodríguez-García V, et al. Computerized cognitive remediation therapy, REHACOM, in first episode of schizophrenia: A randomized controlled trial. Psychiatry Res 2019; 281: 112563.
33. Garrido G, Barrios M, Penadés R, et al. Computer-assisted cognitive remediation therapy: cognition, self-esteem and quality of life in schizophrenia. Schizophr Res 2013; 150: 563–9.
34. Gawęda Ł, Krężołek M, Olbryś J, Turska A, Kokoszka A. Decreasing self-reported cognitive biases and increasing clinical insight through meta-cognitive training in patients with chronic schizophrenia. J Behav Ther Exp Psychiatry 2015; 48: 98–104.
35. Gharaeipour M, Scott BJ. Effects of cognitive remediation on neurocognitive functions and psychiatric symptoms in schizophrenia inpatients. Schizophr Res 2012; 142: 165–70.
36. Gohar SM, Hamdi E, El Ray LA, Horan WP, Green MF. Adapting and evaluating a social cognitive remediation program for schizophrenia in Arabic. Schizophr Res 2013; 148: 12–7.
37. Gomar JJ, Valls E, Radua J, et al. A Multisite, Randomized Controlled Clinical Trial of Computerized Cognitive Remediation Therapy for Schizophrenia. Schizophr Bull 2015; 41: 1387–96.
38. Gordon A, Davis PJ, Patterson S, et al. A randomized waitlist control community study of Social Cognition and Interaction Training for people with schizophrenia. Br J Clin Psychol 2018; 57: 116–30.
39. Greig TC, Zito W, Wexler BE, Fiszdon J, Bell MD. Improved cognitive function in schizophrenia after one year of cognitive training and vocational services. Schizophr Res 2007; 96: 156–61.
40. Habel U, Koch K, Kellermann T, et al. Training of affect recognition in schizophrenia: Neurobiological correlates. Soc Neurosci 2010; 5: 92–104.
41. Hadas-Lidor N, Katz N, Tyano S, Weizman A. Effectiveness of dynamic cognitive intervention in rehabilitation of clients with schizophrenia. Clin Rehabil 2001; 15: 349–59.
42. Hegde S, Rao SL, Raguram A, Gangadhar BN. Addition of home-based cognitive retraining to treatment as usual in first episode schizophrenia patients: a randomized controlled study. Indian J Psychiatry 2012; 54: 15–22.
43. Hermanutz M, Gestrich J. Computer-assisted attention training in schizophrenics. A comparative study. Eur Arch Psychiatry Clin Neurosci 1991; 240: 282–7.
44. Hodge MAR, Siciliano D, Withey P, et al. A Randomized Controlled Trial of Cognitive Remediation in Schizophrenia. Schizophr Bull 2010; 36: 419–27.
45. Hogarty GE, Flesher S, Ulrich R, et al. Cognitive enhancement therapy for schizophrenia: effects of a 2-year randomized trial on cognition and behavior. Arch Gen Psychiatry 2004; 61: 866–76.
46. Hooker CI, Bruce L, Fisher M, Verosky SC, Miyakawa A, Vinogradov S. Neural activity during emotion recognition after combined cognitive plus social cognitive training in schizophrenia. Schizophr Res 2012; 139: 53–9.
47. Horan WP, Kern RS, Shokat-Fadai K, Sergi MJ, Wynn JK, Green MF. Social cognitive skills training in schizophrenia: an initial efficacy study of stabilized outpatients. Schizophr Res 2009; 107: 47–54.

© 2021 Vita et al. *JAMA Psychiatry.*

48. Horan WP, Kern RS, Tripp C, et al. Efficacy and specificity of Social Cognitive Skills Training for outpatients with psychotic disorders. J Psychiatr Res 2011; 45: 1113–22.
49. Horan WP, Dolinsky M, Lee J, et al. Social Cognitive Skills Training for Psychosis With Community-Based Training Exercises: A Randomized Controlled Trial. Schizophr Bull 2018; 44: 1254–66.
50. Iwata K, Matsuda Y, Sato S, et al. Efficacy of cognitive rehabilitation using computer software with individuals living with schizophrenia: A randomized controlled trial in Japan. Psychiatr Rehabil J 2017; 40: 4–11.
51. Jahshan C, Vinogradov S, Wynn JK, Hellemann G, Green MF. A randomized controlled trial comparing a 'bottom-up' and 'top-down' approach to cognitive training in schizophrenia. J Psychiatr Res 2019; 109: 118–25.
52. Kanie A, Kikuchi A, Haga D, et al. The Feasibility and Efficacy of Social Cognition and Interaction Training for Outpatients With Schizophrenia in Japan: A Multicenter Randomized Clinical Trial. Front Psychiatry 2019; 10: 589.
53. Kantrowitz JT, Sharif Z, Medalia A, et al. A Multicenter, Rater-Blinded, Randomized Controlled Study of Auditory Processing-Focused Cognitive Remediation Combined With Open-Label Lurasidone in Patients With Schizophrenia and Schizoaffective Disorder. J Clin Psychiatry 2016; 77: 799–806.
54. Katsumi A, Hoshino H, Fujimoto S, et al. Effects of cognitive remediation on cognitive and social functions in individuals with schizophrenia. Neuropsychol Rehabil 2019; 29: 1475–87.
55. Keefe RSE, Vinogradov S, Medalia A, et al. Feasibility and pilot efficacy results from the multisite Cognitive Remediation in the Schizophrenia Trials Network (CRSTN) randomized controlled trial. J Clin Psychiatry 2012; 73: 1016–22.
56. Kidd SA, Kaur J, Virdee G, George TP, McKenzie K, Herman Y. Cognitive remediation for individuals with psychosis in a supported education setting: a randomized controlled trial. Schizophr Res 2014; 157: 90–8.
57. Klingberg S, Wölwer W, Engel C, et al. Negative symptoms of schizophrenia as primary target of cognitive behavioral therapy: results of the randomized clinical TONES study. Schizophr Bull 2011; 37 Suppl 2: S98-110.
58. Kukla M, Bell MD, Lysaker PH. A randomized controlled trial examining a cognitive behavioral therapy intervention enhanced with cognitive remediation to improve work and neurocognition outcomes among persons with schizophrenia spectrum disorders. Schizophr Res 2018; 197: 400–6.
59. Kumar D, Haq MZU, Dubey I, et al. Effect of meta-cognitive training in the reduction of positive symptoms in schizophrenia. Eur J Psychother Couns 2010; 12: 149–58.
60. Kurtz MM, Seltzer JC, Shagan DS, Thime WR, Wexler BE. Computer-Assisted Cognitive Remediation in Schizophrenia: What is the Active Ingredient? Schizophr Res 2007; 89: 251–60.
61. Kurtz MM, Mueser KT, Thime WR, Corbera S, Wexler BE. Social Skills Training and Computer-Assisted Cognitive Remediation in Schizophrenia. Schizophr Res 2015; 162: 35–41.
62. Lado-Codesido M, Pérez CM, Mateos R, Olivares JM, Caballero AG. Improving emotion recognition in schizophrenia with 'VOICES': An on-line prosodic self-training. PLOS ONE 2019; 14: e0210816.
63. Lee WK. Effectiveness of computerized cognitive rehabilitation training on symptomatological, neuropsychological and work function in patients with schizophrenia. Asia-Pac Psychiatry 2013; 5: 90–100.
64. Lindenmayer J-P, McGurk SR, Mueser KT, et al. A randomized controlled trial of cognitive remediation among inpatients with persistent mental illness. Psychiatr Serv Wash DC 2008; 59: 241–7.
65. Lu H, Li Y, Li F, et al. Randomized controlled trial on adjunctive cognitive remediation therapy for chronically hospitalized patients with schizophrenia. Shanghai Arch Psychiatry 2012; 24: 149–54.
66. Mahncke HW, Kim S-J, Rose A, et al. Evaluation of a plasticity-based cognitive training program in schizophrenia: Results from the eCaesar trial. Schizophr Res 2019; 208: 182–9.
67. Mak M, Samochowiec J, Tybura P, et al. The efficacy of cognitive rehabilitation with RehaCom programme in schizophrenia patients. The role of selected genetic polymorphisms in successful cognitive rehabilitation. Ann Agric Environ Med 2013; 20. http://yadda.icm.edu.pl/yadda/element/bwmeta1.element.agro-f8ce606e-213f-45cd-9ff1-80554003aef0 (accessed May 13, 2020).
68. Man DWK, Law KM, Chung RCK. Cognitive training for Hong Kong Chinese with schizophrenia in vocational rehabilitation. Hong Kong Med J Xianggang Yi Xue Za Zhi 2012; 18 Suppl 6: 18–22.
69. Maroño Souto Y, Vázquez Campo M, Díaz Llenderrozas F, Rodríguez Álvarez M, Mateos R, García Caballero A. Randomized Clinical Trial with e-MotionalTraining® 1.0 for Social Cognition Rehabilitation in Schizophrenia. Front Psychiatry 2018; 9: 40.
70. Matsuda Y, Morimoto T, Furukawa S, et al. Feasibility and effectiveness of a cognitive remediation programme with original computerised cognitive training and group intervention for schizophrenia: a multicentre randomised trial. Neuropsychol Rehabil 2018; 28: 387–97.

© 2021 Vita et al. *JAMA Psychiatry.*

71. Matsui M, Arai H, Yonezawa M, Sumiyoshi T, Suzuki M, Kurachi M. The effects of cognitive rehabilitation on social knowledge in patients with schizophrenia. Appl Neuropsychol 2009; 16: 158–64.
72. McGurk SR, Mueser KT, Pascaris A. Cognitive training and supported employment for persons with severe mental illness: one-year results from a randomized controlled trial. Schizophr Bull 2005; 31: 898–909.
73. McGurk SR, Mueser KT, Xie H, et al. Cognitive remediation for vocational rehabilitation nonresponders. Schizophr Res 2016; 175: 48–56.
74. Medalia A, Aluma M, Tryon W, Merriam AE. Effectiveness of attention training in schizophrenia. Schizophr Bull 1998; 24: 147–52.
75. Medalia A, Revheim N, Casey M. Remediation of memory disorders in schizophrenia. Psychol Med 2000; 30: 1451–9.
76. Meichenbaum D, Cameron R. Training schizophrenics to talk to themselves: A means of developing attentional controls. Behav Ther 1973; 4: 515–34.
77. Mendella PD, Burton CZ, Tasca GA, Roy P, St Louis L, Twamley EW. Compensatory cognitive training for people with first-episode schizophrenia: results from a pilot randomized controlled trial. Schizophr Res 2015; 162: 108–11.
78. Morimoto T, Matsuda Y, Matsuoka K, et al. Computer-assisted cognitive remediation therapy increases hippocampal volume in patients with schizophrenia: a randomized controlled trial. BMC Psychiatry 2018; 18. DOI:10.1186/s12888-018-1667-1.
79. Moritz S, Kerstan A, Veckenstedt R, et al. Further evidence for the efficacy of a metacognitive group training in schizophrenia. Behav Res Ther 2011; 49: 151–7.
80. Moritz S, Thoering T, Kühn S, Willenborg B, Westermann S, Nagel M. Metacognition-augmented cognitive remediation training reduces jumping to conclusions and overconfidence but not neurocognitive deficits in psychosis. Front Psychol 2015; 6. DOI:10.3389/fpsyg.2015.01048.
81. Müller DR, Schmidt SJ, Roder V. One-year randomized controlled trial and follow-up of integrated neurocognitive therapy for schizophrenia outpatients. Schizophr Bull 2015; 41: 604–16.
82. Müller DR, Khalesi Z, Benzing V, et al. Does Integrated Neurocognitive Therapy (INT) reduce severe negative symptoms in schizophrenia outpatients? Schizophr Res 2017; 188: 92–7.
83. O'Reilly K, Donohoe G, O'Sullivan D, et al. A randomized controlled trial of cognitive remediation for a national cohort of forensic patients with schizophrenia or schizoaffective disorder. BMC Psychiatry 2019; 19: 27.
84. Ochoa S, López-Carrilero R, Barrigón ML, et al. Randomized control trial to assess the efficacy of metacognitive training compared with a psycho-educational group in people with a recent-onset psychosis. Psychol Med 2017; 47: 1573–84.
85. Ojeda N, Peña J, Sánchez P, et al. Efficiency of cognitive rehabilitation with REHACOP in chronic treatment resistant Hispanic patients. NeuroRehabilitation 2012; 30: 65–74.
86. Omiya H, Yamashita K, Miyata T, et al. Pilot Study of the Effects of Cognitive Remediation Therapy Using the Frontal/Executive Program for Treating Chronic Schizophrenia. Open Psychol J 2016; 09. DOI:10.2174/1874350101609010121.
87. Østergaard Christensen T, Vesterager L, Krarup G, et al. Cognitive remediation combined with an early intervention service in first episode psychosis. Acta Psychiatr Scand 2014; 130: 300–10.
88. Peña J, Ibarretxe-Bilbao N, Sánchez P, et al. Combining social cognitive treatment, cognitive remediation, and functional skills training in schizophrenia: a randomized controlled trial. Npj Schizophr 2016; 2: 1–7.
89. Penadés R, Catalán R, Salamero M, et al. Cognitive remediation therapy for outpatients with chronic schizophrenia: a controlled and randomized study. Schizophr Res 2006; 87: 323–31.
90. Penadés R, Pujol N, Catalán R, et al. Brain Effects of Cognitive Remediation Therapy in Schizophrenia: A Structural and Functional Neuroimaging Study. Biol Psychiatry 2013; 73: 1015–23.
91. Penadés R, López-Vílchez I, Catalán R, et al. BDNF as a marker of response to cognitive remediation in patients with schizophrenia: A randomized and controlled trial. Schizophr Res 2018; 197: 458–64.
92. Pijnenborg GHM, de Vos AE, Timmerman ME, et al. Social cognitive group treatment for impaired insight in psychosis: A multicenter randomized controlled trial. Schizophr Res 2019; 206: 362–9.
93. Pontes LMM, Martins CB, Napolitano IC, et al. Cognitive Training for Schizophrenia in Developing Countries: A Pilot Trial in Brazil. Schizophr Res Treat 2013; 2013. DOI:10.1155/2013/321725.
94. Popova P, Popov TG, Wienbruch C, Carolus AM, Miller GA, Rockstroh BS. Changing facial affect recognition in schizophrenia: Effects of training on brain dynamics. NeuroImage Clin 2014; 6: 156–65.
95. Puig O, Penadés R, Baeza I, et al. Cognitive remediation therapy in adolescents with early-onset schizophrenia: a randomized controlled trial. J Am Acad Child Adolesc Psychiatry 2014; 53: 859–68.

© 2021 Vita et al. *JAMA Psychiatry.*

96. Rakitzi S, Georgila P, Efthimiou K, Mueller DR. Efficacy and feasibility of the Integrated Psychological Therapy for outpatients with schizophrenia in Greece: Final results of a RCT. Psychiatry Res 2016; 242: 137–43.
97. Ramsay IS, Nienow TM, Marggraf MP, MacDonald AW. Neuroplastic changes in patients with schizophrenia undergoing cognitive remediation: triple-blind trial. Br J Psychiatry J Ment Sci 2017; 210: 216–22.
98. Rass O, Forsyth JK, Bolbecker AR, et al. Computer-assisted cognitive remediation for schizophrenia: a randomized single-blind pilot study. Schizophr Res 2012; 139: 92–8.
99. Reeder C, Huddy V, Cella M, et al. A new generation computerised metacognitive cognitive remediation programme for schizophrenia (CIRCuiTS): a randomised controlled trial. Psychol Med 2017; 47: 2720–30.
100. Roberts DL, Combs DR, Willoughby M, et al. A randomized, controlled trial of Social Cognition and Interaction Training (SCIT) for outpatients with schizophrenia spectrum disorders. Br J Clin Psychol 2014; 53: 281–98.
101. Roncone R, Mazza M, Frangou I, et al. Rehabilitation of theory of mind deficit in schizophrenia: a pilot study of metacognitive strategies in group treatment. 2004; published online Sept. DOI:info:doi/10.1080/09602010343000291.
102. Royer A, Grosselin A, Bellot C, et al. Is there any impact of cognitive remediation on an ecological test in schizophrenia? Cognit Neuropsychiatry 2012; 17: 19–35.
103. Sachs G, Winklbaur B, Jagsch R, et al. Training of affect recognition (TAR) in schizophrenia--impact on functional outcome. Schizophr Res 2012; 138: 262–7.
104. Sánchez P, Peña J, Bengoetxea E, et al. Improvements in negative symptoms and functional outcome after a new generation cognitive remediation program: a randomized controlled trial. Schizophr Bull 2014; 40: 707–15.
105. Sartory G, Zorn C, Groetzinger G, Windgassen K. Computerized cognitive remediation improves verbal learning and processing speed in schizophrenia. Schizophr Res 2005; 75: 219–23.
106. Sevos J, Grosselin A, Gauthier M, Carmona F, Gay A, Massoubre C. Cinemotion, a Program of Cognitive Remediation to Improve the Recognition and Expression of Facial Emotions in Schizophrenia: A Pilot Study. Front Psychiatry 2018; 9. DOI:10.3389/fpsyt.2018.00312.
107. Silverstein SM, Hatashita-Wong M, Solak BA, et al. Effectiveness of a two-phase cognitive rehabilitation intervention for severely impaired schizophrenia patients. Psychol Med 2005; 35: 829–37.
108. Silverstein SM, Spaulding WD, Menditto AA, et al. Attention Shaping: a Reward-Based Learning Method to Enhance Skills Training Outcomes in Schizophrenia. Schizophr Bull 2009; 35: 222–32.
109. So SH-W, Chan AP, Chong CS-Y, et al. Metacognitive training for delusions (MCTd): effectiveness on data-gathering and belief flexibility in a Chinese sample. Front Psychol 2015; 6: 730.
110. Spaulding WD, Reed D, Sullivan M, Richardson C, Weiler M. Effects of cognitive treatment in psychiatric rehabilitation. Schizophr Bull 1999; 25: 657–76.
111. Tan B-L, King R. The effects of cognitive remediation on functional outcomes among people with schizophrenia: a randomised controlled study. Aust N Z J Psychiatry 2013; 47: 1068–80.
112. Tan S, Zou Y, Wykes T, et al. Group cognitive remediation therapy for chronic schizophrenia: A randomized controlled trial. Neurosci Lett 2016; 626: 106–11.
113. Tan S, Zhu X, Fan H, et al. Who will benefit from computerized cognitive remediation therapy? Evidence from a multisite randomized controlled study in schizophrenia. Psychol Med 2019; : 1–11.
114. Tao J, Zeng Q, Liang J, Zhou A, Yin X, Xu A. Effects of cognitive rehabilitation training on schizophrenia: 2 years of follow-up. Int J Clin Exp Med 2015; 8: 16089–94.
115. Tas C, Danaci AE, Cubukcuoglu Z, Brüne M. Impact of family involvement on social cognition training in clinically stable outpatients with schizophrenia -- a randomized pilot study. Psychiatry Res 2012; 195: 32–8.
116. Thomas ML, Bismark AW, Joshi YB, et al. Targeted cognitive training improves auditory and verbal outcomes among treatment refractory schizophrenia patients mandated to residential care. Schizophr Res 2018; 202: 378–84.
117. Twamley EW, Vella L, Burton CZ, Heaton RK, Jeste DV. Compensatory cognitive training for psychosis: effects in a randomized controlled trial. J Clin Psychiatry 2012; 73: 1212–9.
118. Ueland T, Rund BR. A controlled randomized treatment study: the effects of a cognitive remediation program on adolescents with early onset psychosis. Acta Psychiatr Scand 2004; 109: 70–4.
119. van Oosterhout B, Krabbendam L, de Boer K, et al. Metacognitive group training for schizophrenia spectrum patients with delusions: a randomized controlled trial. Psychol Med 2014; 44: 3025–35.
120. Vaskinn A, Løvgren A, Egeland MK, et al. A randomized controlled trial of training of affect recognition (TAR) in schizophrenia shows lasting effects for theory of mind. Eur Arch Psychiatry Clin Neurosci 2019; 269: 611–20.

© 2021 Vita et al. *JAMA Psychiatry.*

121. Vauth R, Corrigan PW, Clauss M, et al. Cognitive strategies versus self-management skills as adjunct to vocational rehabilitation. Schizophr Bull 2005; 31: 55–66.
122. Ventura J, Subotnik KL, Gretchen-Doorly D, et al. Cognitive remediation can improve negative symptoms and social functioning in first-episode schizophrenia: A randomized controlled trial. Schizophr Res 2019; 203: 24–31.
123. Vidarsdottir OG, Roberts DL, Twamley EW, Gudmundsdottir B, Sigurdsson E, Magnusdottir BB. Integrative cognitive remediation for early psychosis: Results from a randomized controlled trial. Psychiatry Res 2019; 273: 690–8.
124. Vita A, De Peri L, Barlati S, et al. Effectiveness of different modalities of cognitive remediation on symptomatological, neuropsychological, and functional outcome domains in schizophrenia: a prospective study in a real-world setting. Schizophr Res 2011; 133: 223–31.
125. Vita A, De Peri L, Barlati S, et al. Psychopathologic, neuropsychological and functional outcome measures during cognitive rehabilitation in schizophrenia: a prospective controlled study in a real-world setting. Eur Psychiatry J Assoc Eur Psychiatr 2011; 26: 276–83.
126. Wölwer W, Frommann N, Halfmann S, Piaszek A, Streit M, Gaebel W. Remediation of impairments in facial affect recognition in schizophrenia: efficacy and specificity of a new training program. Schizophr Res 2005; 80: 295–303.
127. Wykes T, Reeder C, Corner J, Williams C, Everitt B. The effects of neurocognitive remediation on executive processing in patients with schizophrenia. Schizophr Bull 1999; 25: 291–307.
128. Wykes T, Newton E, Landau S, Rice C, Thompson N, Frangou S. Cognitive remediation therapy (CRT) for young early onset patients with schizophrenia: an exploratory randomized controlled trial. Schizophr Res 2007; 94: 221–30.
129. Wykes T, Reeder C, Landau S, et al. Cognitive remediation therapy in schizophrenia: randomised controlled trial. Br J Psychiatry J Ment Sci 2007; 190: 421–7.
130. Zimmer M, Duncan AV, Laitano D, Ferreira EE, Belmonte-de-Abreu P. A twelve-week randomized controlled study of the cognitive-behavioral Integrated Psychological Therapy program: positive effect on the social functioning of schizophrenic patients. Rev Bras Psiquiatr Sao Paulo Braz 1999 2007; 29: 140–7.

References of ongoing studies

1. Lopez-Morinigo J-D, Ruiz-Ruano VG, Martínez ASE, et al. Study protocol of a randomised clinical trial testing whether metacognitive training can improve insight and clinical outcomes in schizophrenia. BMC Psychiatry 2020; 20: 30.
2. Nijman SA, Veling W, Greaves-Lord K, et al. Dynamic Interactive Social Cognition Training in Virtual Reality (DiSCoVR) for social cognition and social functioning in people with a psychotic disorder: study protocol for a multicenter randomized controlled trial. BMC Psychiatry 2019; 19: 272.

References of inspected reviews

1. Anaya C, Martinez Aran A, Ayuso-Mateos JL, Wykes T, Vieta E, Scott J. A systematic review of cognitive remediation for schizo-affective and affective disorders. J Affect Disord 2012; 142: 13–21.
2. Bighelli I, Salanti G, Huhn M, et al. Psychological interventions to reduce positive symptoms in schizophrenia: systematic review and network meta-analysis. World Psychiatry 2018; 17: 316–29.
3. Cella M, Preti A, Edwards C, Dow T, Wykes T. Cognitive remediation for negative symptoms of schizophrenia: A network meta-analysis. Clin Psychol Rev 2017; 52: 43–51.
4. Cella M, Price T, Corboy H, Onwumere J, Shergill S, Preti A. Cognitive remediation for inpatients with psychosis: a systematic review and meta-analysis. Psychol Med 2020; 50: 1–15.
5. Chan JYC, Hirai HW, Tsoi KKF. Can computer-assisted cognitive remediation improve employment and productivity outcomes of patients with severe mental illness? A meta-analysis of prospective controlled trials. J Psychiatr Res 2015; 68: 293–300.
6. Eichner C, Berna F. Acceptance and Efficacy of Metacognitive Training (MCT) on Positive Symptoms and Delusions in Patients With Schizophrenia: A Meta-analysis Taking Into Account Important Moderators. Schizophr Bull 2016; 42: 952–62.

© 2021 Vita et al. *JAMA Psychiatry.*

7. Grant N, Lawrence M, Preti A, Wykes T, Cella M. Social cognition interventions for people with schizophrenia: a systematic review focussing on methodological quality and intervention modality. Clin Psychol Rev 2017; 56: 55–64.
8. Grynszpan O, Perbal S, Pelissolo A, et al. Efficacy and specificity of computer-assisted cognitive remediation in schizophrenia: a meta-analytical study. Psychol Med 2011; 41: 163–73.
9. Hegde S. A review of Indian research on cognitive remediation for schizophrenia. Asian J Psychiatry 2017; 25: 54–9.
10. JIANG J, ZHANG L, ZHU Z, LI W, LI C. Metacognitive training for schizophrenia: a systematic review. Shanghai Arch Psychiatry 2015; 27: 149–57.
11. Kambeitz-Ilankovic L, Betz LT, Dominke C, et al. Multi-outcome meta-analysis (MOMA) of cognitive remediation in schizophrenia: Revisiting the relevance of human coaching and elucidating interplay between multiple outcomes. Neurosci Biobehav Rev 2019; 107: 828–45.
12. Kluwe-Schiavon B, Sanvicente-Vieira B, Kristensen CH, Grassi-Oliveira R. Executive functions rehabilitation for schizophrenia: a critical systematic review. J Psychiatr Res 2013; 47: 91–104.
13. Kurtz MM, Richardson CL. Social cognitive training for schizophrenia: a meta-analytic investigation of controlled research. Schizophr Bull 2012; 38: 1092–104.
14. Liu Y-C, Tang C-C, Hung T-T, Tsai P-C, Lin M-F. The Efficacy of Metacognitive Training for Delusions in Patients With Schizophrenia: A Meta-Analysis of Randomized Controlled Trials Informs Evidence-Based Practice. Worldviews Evid Based Nurs 2018; 15: 130–9.
15. McGurk SR, Twamley EW, Sitzer DI, McHugo GJ, Mueser KT. A meta-analysis of cognitive remediation in schizophrenia. Am J Psychiatry 2007; 164: 1791–802.
16. Prikken M, Konings MJ, Lei WU, Begemann MJH, Sommer IEC. The efficacy of computerized cognitive drill and practice training for patients with a schizophrenia-spectrum disorder: A meta-analysis. Schizophr Res 2019; 204: 368–74.
17. Revell ER, Neill JC, Harte M, Khan Z, Drake RJ. A systematic review and meta-analysis of cognitive remediation in early schizophrenia. Schizophr Res 2015; 168: 213–22.
18. Roder V, Mueller DR, Schmidt SJ. Effectiveness of integrated psychological therapy (IPT) for schizophrenia patients: a research update. Schizophr Bull 2011; 37 Suppl 2: S71-79.
19. Tan B-L, Lee S-A, Lee J. Social cognitive interventions for people with schizophrenia: A systematic review. Asian J Psychiatry 2018; 35: 115–31.
20. Turner DT, van der Gaag M, Karyotaki E, Cuijpers P. Psychological interventions for psychosis: a meta-analysis of comparative outcome studies. Am J Psychiatry 2014; 171: 523–38.
21. van Duin D, de Winter L, Oud M, Kroon H, Veling W, van Weeghel J. The effect of rehabilitation combined with cognitive remediation on functioning in persons with severe mental illness: systematic review and meta-analysis. Psychol Med 2019; 49: 1414–25.
22. Wykes T, Huddy V, Cellard C, McGurk SR, Czobor P. A meta-analysis of cognitive remediation for schizophrenia: methodology and effect sizes. Am J Psychiatry 2011; 168: 472–85.

© 2021 Vita et al. *JAMA Psychiatry.*

eTable 2. Cognitive remediation programs adopted in included studies.

Categories of CR programs	Single CR programs applied in studies
**Computer-based neurocognitive interventions (CACR)**	**BrainHQ software:** Thomas 2018<br>**Captain's Log software:** Burda 1994, Bellucci 2003, Au 2015<br>**CineMotion:** Sevos 2018<br>**Computerized Interactive Remediation of Cognition-Training for Schizophrenia (CIRCuiTS)**: Drake 2014, Reeder 2017<br>**Cogpack**: McGurk 2005, Sartory 2005, Wölwer 2005 (Cogpack arm), Lindenmayer 2008 (+group discussion), Klingberg 2011, Vita 2011 (CACR arm), Kidd 2014 (+group discussion), Jahshan 2019 (Cogpack arm)<br>**Cog-trainer software:** Lee 2013<br>**Computer Drill Training:** Byrne 2013<br>**FesKits program:** Gomar 2015<br>**Japanese Cognitive Rehabilitation Program for Schizophrenia (JCORES)**: Morimoto 2018<br>**MyBrainGymmer software:** Cassetta 2019<br>**MyBrainTraining software:** Moritz 2015 (home-based)<br>**NEUROCOM program:** Østergaard Christensen 2014<br>**NPT-MH software:** Fernandez-Gonzalo 2015<br>**PositScience software, Auditory**: Fisher 2009-2016, Fisher 2015; **Aristotle:** Horan 2011 (NR arm); **Brain Fitness:** Kukla 2018, Jahshan 2019 (PositScience arm); **multiple tasks**: Rass 2012, Mahncke 2019 (including novel exercises)<br>**PSSCogrehab software**: D'Souza 2013, Tan 2013, Fiszdon 2016, Choi 2017<br>**RehaCom software**: D'Amato 2011, Royer 2012, Mak 2013, Garcia-Fernandez 2019<br>**Unspecified/Other programs used in single studies**: Kurtz 2007, Dickinson 2010, Man 2012 (CAEL arm), Garrido 2013, Kurtz 2015, Fan 2017, Tan 2019
**Pencil-and-paper neurocognitive interventions**	**Compensatory Cognitive Training (CCT)**: Twamley 2008-2012, Mendella 2015<br>**Cognitive Remediation Therapy (CRT)**: Wykes 1999, Wykes 2007a, Wykes 2007b, Lu 2012, Puig 2014, Tan 2016, Pijnenborg 2019 (adapted from CRT)<br>**Frontal/Executive Program**: Penades 2006, Penades 2013, Omiya 2016, Penades 2018<br>**Other programs used in single studies:** Gharaeipour 2012 (own program), Hegde 2012 (own program, home-based), Man 2012 (TAEL arm), Tao 2015
**Interventions targeting single neurocognitive domains**	**Attention training**: Hermanutz 1991, Medalia 1998 (ORM software)<br>**Attention Processing Training and Shaping**: Silverstein 2005, Silverstein 2009<br>**Cognitive Attention and Memory Training**: Pontes 2013<br>**Memory training**: Medalia 2000 (1st arm)<br>**Problem Solving Training (Where in the USA is Carmen Sandiego? software)**: Medalia 2000 (2nd arm)<br>**Working Memory Training**: Donohoe 2018 (low-support), Ramsay 2017<br>**Auditory Discrimination+Working Memory training**: Popova 2014 (CE arm)
**Interventions targeting Social Cognition**	**eMotional Training**: Maroño Souto 2018, Lado-Codesido 2019 (Voices)<br>**Facial Affect recognition Training**: Popova 2014 (FAT arm)<br>**Social Cognition Enhancement Training (SCET)**: Choi 2006<br>**Social Cognitive Interaction Training (SCIT)**: Roberts 2014, Gordon 2018, Kanie 2019, Tas 2012 (family version)<br>**Social Cognitive Skills Training (SCST)**: Horan 2009, Horan 2011 (SCST arm), Gohar 2013, Horan 2018<br>**Social Perception Training**: Matsui 2009<br>**Training of Affect Recognition (TAR)**: Wölwer 2005 (TAR arm), Habel 2010, Sachs 2012, Vaskinn 2019
**Interventions based on an integrative approach**	**Cognitive Enhancement Therapy (CET)**: Hogarty 2004, Eack 2009<br>**Instrumental Enrichment Program**: Hadas-Lidor 2001, Roncone 2004<br>**Integrated Neurocognitive Therapy (INT)**: Müller 2015, Müller 2017<br>**Integrated Psychological Therapy (IPT)**: Hermanutz 1991 (IPT arm, selected subprograms), Spaulding 1999, Garcia 2003 (cognitive subprograms), Zimmer 2007 (cognitive subprograms), Vita 2011a (IPT arm, cognitive subprograms), Vita 2011b (cognitive subprograms), Rakitzi 2016 (cognitive subprograms), Aloi 2018, Ueland 2004 (own program based on IPT)<br>**Neuropsychological and Educational Approach to Remediation (NEAR)**: Hodge 2010, Katsumi 2019<br>**Neurocognitive Enhancement Therapy (NET)**: Bell 2001, Greig 2007<br>**Cognitive Rehabilitation Program in Psychosis (REHACOP):** Ojeda 2012, Peña 2016, Sanchez 2014 (integrated version)<br>**Problem Solving and Cognitive Flexibility (REPYFLEC)**: Farreny 2012<br>**Social Skills and Neurocognitive Individualized Training (SSANIT)**: Galderisi 2010<br>**Thinking Skills for Work**: Bowie 2012, McGurk 2016, Iwata 2017<br>**Other programs used in single studies**: O'Reilly 2019 (manualized)
**Metacognitive Training (MCT)**	Aghotor 2010, Kumar 2010, Moritz 2011, Briki 2014, Favrod 2014, Van Oosterhout 2014, Gaweda 2015, So 2015, Ochoa 2017, Ahuir 2018

© 2021 Vita et al. *JAMA Psychiatry.*

**Combination of multiple interventions**	**CACR+SCIT+NEAR**: Vidarsdottir 2019<br>**Cogpack+Cognitive Adaptation Training (CAT)**: Vauth 2005<br>**JCORES+group sessions focused on metacognition:** Matsuda 2016<br>**NET+NEAR:** Ventura 2019<br>**PositScience software+NEAR bridging group**: Keefe 2012, Ahmed 2015, Kantrowitz 2016<br>**PositScience software+computer-based Social cognition training**: Hooker 2012<br>**PositScience software+SCST**: Horan 2011 (Hybrid arm)
**Other interventions**	**Self-instructional Training**: Meichenbaum 1973

© 2021 Vita et al. *JAMA Psychiatry.*

eTable 3. Summary of individual characteristics of included studies.

Study	Country, Design and Setting	Characteristics of included patients	Sample and Attrition	Duration (weeks)	Treatment Program and Schedule	Comparison	Quality Rating (CTAM)	Outcome Measures (included in the analyses)
Aghotor 2010	Germany, single center<br><br>Inpatients<br><br>Chronic, with active positive symptoms	Gender: 33,3% female<br>Age: 30,6 years<br>Illness duration: 11,6 years<br>Onset: 19,1 years<br>Education: n.i.<br>IQ: n.i.<br>Diagnosis: non-affective psychosis<br>Baseline PANSS: n.i.<br>Daily drug dose (CPZeq): n.i.	N=30, 13% attrition rate	4	Metacognitive Training (MCT), 2 sessions/week of 45-60min each, N=16	Newspaper reading groups, N=14	62	Positive Symptoms
Ahmed 2015	USA, single center<br><br>Inpatients in forensic setting	Gender: 13% female<br>Age: 40,5 years<br>Illness duration: 18,6 years<br>Onset: 21,9 years<br>Education: 9,8 years<br>IQ: 94,2<br>Diagnosis: schizophrenia (69%), schizoaffective disorder<br>Baseline PANSS: n.i.<br>Daily drug dose (CPZeq): 705,4 mg/day	N=78, 19% attrition rate	20	Computer-assisted remediation (mainly Posit Science tasks) + Bridging group discussions, 3 sessions/week of 1h each, with regular psychosocial rehabilitation, N=42	Computer games + Healthy behavior group discussions, with regular psychosocial rehabilitation, N=36	79	Global Cognition<br>All cognitive domains<br>Global Functioning<br>Global Symptoms<br>Positive Symptoms<br>Negative Symptoms
Ahuir 2018	Spain, multi-center, crossover trial<br><br>Outpatients<br><br>Recent onset (<3 years)	Gender: 59,2% female<br>Age: 23,6 years<br>Illness duration, onset and education: n.i.<br>IQ: n.i.<br>Diagnosis: first episode of psychosis<br>Baseline PANSS: 50,3<br>Daily drug dose (CPZeq): n.i.	N=49, 26,5% attrition rate	8	Metacognitive Training (MCT), 1h/week, N=22	Psychoeducation, N=29	39	Global Cognition<br>Social Cognition<br>Global Functioning<br>Global Symptoms<br>Positive Symptoms<br>Negative Symptoms
Aloi 2018	Italy, single center<br><br>Inpatients	Gender: 29% female<br>Age: 51,1 years<br>Illness duration: 23,1 years<br>Onset: 27,9 years<br>Education: n.i.<br>IQ: n.i.	N=46, 11% attrition rate	36	Integrated Psychological Therapy (IPT), 2 sessions/week of 45min each, N=24	Treatment as usual, N=22	55	Global Cognition<br>Processing Speed<br>Working Memory<br>Executive Functions<br>Social Cognition<br>Global Functioning<br>Global Symptoms

© 2021 Vita et al. *JAMA Psychiatry.*

		Diagnosis: schizophrenia<br>Baseline PANSS: 91,6<br>Daily drug dose (CPZeq): 652,8 mg/day						Positive Symptoms<br>Negative Symptoms
Au 2015	Hong Kong, single center<br><br>Outpatients	Gender: 36,7% female<br>Age: 36,1 years<br>Illness duration: 11,2 years<br>Onset: 24,9 years<br>Education: 14,9 years<br>IQ: n.i.<br>Diagnosis: schizophrenia (58%), schizoaffective disorder<br>Baseline PANSS: 41,9<br>Daily drug dose (CPZeq): n.i.	N=90, 10% attrition rate	12	Computer-assisted remediation, 3 sessions/week of 2h each + Integrated Supported Employment, N=45	Time-matched TV watching + Integrated Supported Employment, N=45	73	Global Cognition<br>Attention/Vigilance<br>Processing Speed<br>Verbal Memory<br>Visual Memory<br>Executive Functions<br>Social Cognition<br>Global Functioning<br>Global Symptoms
Bell 2001	USA, multi-center<br><br>Outpatients	Gender: 26,2% female<br>Age: 41,3 years<br>Illness duration: 19,3 years<br>Onset: 22 years<br>Education: 13,3 years<br>IQ: 87,6<br>Diagnosis: schizophrenia (69%), schizoaffective disorder<br>Baseline PANSS: n.i.<br>Daily drug dose (CPZeq): 743,5 mg/day	N=65, dropouts n.i.	22	Neurocognitive Enhancement Therapy (NET), 3-4 sessions/week, + Work therapy N=31	Work therapy, N=34	60	Global Cognition<br>Processing Speed<br>Working Memory<br>Executive Functions<br>Social Cognition
Bellucci 2003	USA, single center<br><br>Outpatients	Gender: 52,9% female<br>Age: 42 years<br>Illness duration: 16,6 years<br>Onset: 25,4 years<br>Education: 12,6 years<br>IQ: n.i.<br>Diagnosis: schizophrenia (47%), schizoaffective disorder<br>Baseline PANSS: n.i.<br>Daily drug dose (CPZeq): n.i.	N=34, no dropouts	8	Computer-assisted remediation using Captain's Log, 2 sessions/week of 90 min each, N=17	Multidisciplinary treatment as usual including skills training and psychoeducation, Waiting list, N=17	65	Global Cognition<br>Processing Speed<br>Working Memory<br>Verbal Memory<br>Negative Symptoms
Bowie 2012	USA, multi-center<br><br>Outpatients	Gender: n.i.<br>Age: 40,6 years	N=114, 27% attrition rate	12	Thinking Skills for Work, 2h/week, N=38	Functional Adaptation Skills Training, N=38	75	Global Cognition<br>Global Functioning

© 2021 Vita et al. *JAMA Psychiatry.*

		Illness duration: 20,2 years<br>Onset: 20,4 years<br>Education: 13,1 years<br>IQ: n.i.<br>Diagnosis: schizophrenia, schizoaffective disorder<br>Baseline PANSS: n.i.<br>Daily drug dose (CPZeq): n.i.			Thinking Skills for Work + Functional Adaptation Skills Training, N=38			Positive Symptoms<br>Negative Symptoms
Briki 2014	France, multi-center<br><br>In- and outpatients<br><br>Chronic, with active positive symptoms	Gender: 34% female<br>Age: 41,1 years<br>Illness duration: 16,2 years<br>Onset: 24,9 years<br>Education: 8,8 years<br>IQ: n.i.<br>Diagnosis: schizophrenia spectrum disorder<br>Baseline PANSS: 86,6<br>Daily drug dose (CPZeq): 1437,5 mg/day	N=68, 26,5% attrition rate	8	Metacognitive training (MCT), 2 sessions/week of 45.60min each, N=35	Supportive therapy including psychoeducation elements, N=33	71	Global Functioning<br>Positive Symptoms
Bryce 2018	Australia, single center<br><br>In- and outpatients	Gender: 30% female<br>Age: 41 years<br>Illness duration: 14,1 years<br>Onset: 26,9 years<br>Education: 13,1 years<br>IQ: 98,1<br>Diagnosis: schizophrenia (71%), schizoaffective disorder<br>Baseline PANSS: 61,1<br>Daily drug dose (CPZeq): 703,8 mg/day	N=56, 23% attrition rate	10	Computer-assisted remediation using Cogpack, 2 sessions/week of 1h each, N=29	Computer games, N=27	72	Global Cognition<br>All cognitive domains<br>Global Functioning<br>Global Symptoms
Burda 1994	USA, multi-center<br><br>Inpatients	Gender: 2,8% female<br>Age: 46,6 years<br>Illness duration and onset: n.i.<br>Education: 12,5 years<br>IQ: n.i.<br>Diagnosis: schizophrenia (68%), schizoaffective disorder	N=80, 14% attrition rate	8	Computer-assisted remediation using Captain's Log, 3 sessions/week of 30min each, N=40	Treatment as usual, N=40	54	Global Cognition<br>Attention/Vigilance<br>Processing Speed<br>Working Memory<br>Verbal Memory<br>Visual Memory

© 2021 Vita et al. *JAMA Psychiatry.*

		Baseline PANSS: n.i.<br>Daily drug dose (CPZeq): n.i.						
Byrne 2013	China, single center<br><br>Inpatients	Gender: 100% male<br>Age: 45,6 years<br>Illness duration: 20,6 years<br>Onset: 25 years<br>Education: 11,3 years<br>IQ: n.i.<br>Diagnosis: schizophrenia<br>Baseline PANSS: n.i.<br>Daily drug dose (CPZeq): 390,2 mg/day	N=51, 39% attrition rate	6	Own computer-assisted program, schedule not described in detail, N=27	Treatment as usual, N=24	48	Global Cognition<br>Working Memory<br>Verbal Memory<br>Global Functioning<br>Global Symptoms<br>Positive Symptoms<br>Negative Symptoms
Cassetta 2019	Canada, multi-center<br><br>Outpatients	Gender: 39,4% female<br>Age: 40,2 years<br>Illness duration: 13,7 years<br>Onset: 26,6 years<br>Education: 13,3 years<br>IQ: n.i.<br>Diagnosis: schizophrenia (61%), schizoaffective disorder<br>Baseline PANSS: 79,4<br>Daily drug dose (CPZeq): n.i.	N=83, 14% attrition rate	10	Computer-assisted Working Memory training, N=28,<br>or Processing Speed training, N=28, both using MyBrainGymmer, 5 sessions/week of 30min each	Treatment as usual, N=27	82	Global Cognition<br>Processing Speed<br>Working Memory<br>Executive Functions<br>Social Cognition<br>Global Functioning<br>Global Symptoms
Cavallaro 2009	Italy, multi-center<br><br>Inpatients	Gender: n.i.<br>Age: 33,6 years<br>Illness duration: 8,2 years<br>Onset: 25,1 years<br>Education: 11,7 years<br>IQ: n.i.<br>Diagnosis: schizophrenia<br>Baseline PANSS: n.i.<br>Daily drug dose (CPZeq): n.i.	N=100, 14% attrition rate	12	Computer-assisted remediation using Cogpack, 3 sessions/week of 1h each, + Rehabilitation program, N=58	Computer activities + Rehabilitation program, N=42	51	Global Cognition<br>Attention/Vigilance<br>Processing Speed<br>Working Memory<br>Verbal Memory<br>Executive Functions<br>Global Functioning
Choi 2006	South Korea, single center<br><br>Outpatients	Gender: 44% female<br>Age: 32,5 years<br>Illness duration: 11,2 years<br>Onset: 21,3 years<br>Education: 12,4 years<br>IQ: n.i.	N=34, 47% attrition rate	26	Social Cognition Enhancement Training (SCET), 2 sessions/week, N=17	Usual psychiatric rehabilitation program including social skills training, N=17	35	Global Cognition<br>Executive Functions<br>Social Cognition

© 2021 Vita et al. *JAMA Psychiatry.*

		Diagnosis: schizophrenia (97%), schizoaffective disorder<br>Baseline PANSS: n.i.<br>Daily drug dose (CPZeq): n.i.						
Choi 2017	South Korea, single center<br><br>Inpatients	Gender: 42% female<br>Age: 49,7 years<br>Illness duration: 23 years<br>Onset: 26,8 years<br>Education: 11,2 years<br>IQ: 95,9<br>Diagnosis: schizophrenia<br>Baseline PANSS: n.i.<br>Daily drug dose (CPZeq): n.i.	N=38, 3% attrition rate	12	Computer-assisted remediation using PSS CogRehab, 2 sessions/week of 1h each + Comprehensive psychiatric rehabilitation, N=19	Comprehensive psychiatric rehabilitation including skill building therapies, N=19	66	Global Cognition<br>Processing Speed<br>Working Memory<br>Verbal Memory<br>Executive Functions<br>Global Symptoms<br>Positive Symptoms<br>Negative Symptoms
D'Amato 2011	France, multi-center<br><br>Inpatients	Gender: 24,7% female<br>Age: 32,8 years<br>Illness duration and onset: n.i.<br>Education: 12,3 years<br>IQ: 100,3<br>Diagnosis: schizophrenia<br>Baseline PANSS: 74,4<br>Daily drug dose (CPZeq): 388,3 mg/day	N=77, no dropouts	7	Computer-assisted remediation using Rehacom, 2 sessions/week of 2h each, N=39	Treatment as usual, waiting list N=38	72	Global Cognition<br>Attention/Vigilance<br>Processing Speed<br>Working Memory<br>Verbal Memory<br>Visual Memory<br>Executive Functions<br>Global Functioning<br>Global Symptoms<br>Positive Symptoms<br>Negative Symptoms
D'Souza 2013	USA, multi-center<br><br>Outpatients	Gender: 25% female<br>Age: 37,2 years<br>Illness duration: 10,7 years<br>Onset: 26,5 years<br>Education: 12,7 years<br>IQ: 91,2<br>Diagnosis: schizophrenia, schizoaffective disorder<br>Baseline PANSS: 56,9<br>Daily drug dose (CPZeq): 272,5 mg/day	N=53, 15% attrition rate	12	Computer-assisted remediation using PSS CogRehab, 5h/week, N=27<br><br>*Only the arm receiving placebo serine was included in the analysis	TV watching, N=26<br><br>*Only the arm receiving placebo serine was included in the analysis	62	Global Cognition<br>Attention/Vigilance<br>Processing Speed<br>Working Memory<br>Verbal Memory<br>Executive Functions<br>Global Functioning<br>Global Symptoms<br>Positive Symptoms<br>Negative Symptoms
Dickinson 2010	USA, multi-center<br><br>Outpatients	Gender: 35% female<br>Age: 47,6 years<br>Illness duration and onset: n.i.	N=69, 9% attrition rate	15	Computer-assisted remediation, 3sessions/week of 1h each, N=35	Computer games, N=32	82	Global Cognition<br>Processing Speed<br>Working Memory<br>Executive Functions<br>Global Functioning

© 2021 Vita et al. *JAMA Psychiatry.*

		Education: 12,5 years<br>IQ: n.i.<br>Diagnosis: schizophrenia (78%), schizoaffective disorder<br>Baseline PANSS: 66,5<br>Daily drug dose (CPZeq): n.i.						Global Symptoms<br>Negative Symptoms
Donohoe 2018	Ireland, multi-center<br><br>Outpatients	Gender: 60% female<br>Age: 43,3 years<br>Illness duration: 17,1 years<br>Onset: 26,2 years<br>Education: 14 years<br>IQ: n.i.<br>Diagnosis: psychotic disorder (63% schizophrenia; affective psychosis included)<br>Baseline PANSS: n.i.<br>Daily drug dose (CPZeq): 490,4 mg/day	N=90, 39% attrition rate	8	Computer-assisted low-support working memory training, 5 sessions/week of 30-40 min each + 45 min/week with therapist, N=48	Sham intervention, N=42	79	Global Cognition<br>Processing Speed<br>Working Memory<br>Verbal Memory<br>Executive Functions<br>Social Cognition<br>Global Functioning
Drake 2014	Great Britain, multi-center<br><br>Outpatients<br><br>First-episode	Gender: 39,3% female<br>Age: 24,1 years<br>Illness duration and onset: n.i.<br>Education: 12,7 years<br>IQ: 104,4<br>Diagnosis: schizophrenia (85%), schizoaffective disorder<br>Baseline PANSS: 70,4<br>Daily drug dose (CPZeq): n.i.	N=62, 3% attrition rate	12	Computer-assisted remediation using CIRCuiTS, 3-5 sessions/week of 1h each (40h total), followed by CBTp, N=31	Time-matched social contact, followed by CBTp, N=31	83	Global Cognition<br>Visual Memory<br>Executive Functions<br>Global Functioning<br>Global Symptoms<br>Positive Symptoms
Eack 2009	USA, single center<br><br>Outpatients<br><br>Recent onset (<5 years)	Gender: 31% female<br>Age: 28,9 years<br>Illness duration: 3,2 years<br>Onset: 22,7 years<br>Education: n.i.<br>IQ: 98,1<br>Diagnosis: schizophrenia (66%), schizoaffective disorder<br>Baseline PANSS: n.i.	N=58, dropouts n.i.	104	Cognitive Enhancement Therapy (CET), 1h/week of neurocognitive training + 1,5h/week of social-cognitive training, + Enriched supportive therapy, N=31	Enriched supportive therapy (including skills training and psychoeducation), N=27	56	Global Cognition<br>Processing Speed<br>Working Memory<br>Verbal Memory<br>Executive Functions<br>Social Cognition<br>Global Functioning<br>Global Symptoms<br>Positive Symptoms<br>Negative Symptoms

© 2021 Vita et al. *JAMA Psychiatry.*

		Daily drug dose (CPZeq): 418,2 mg/day						
Fan 2017	China, single center<br><br>Inpatients	Gender: 48% female<br>Age: 40,5 years<br>Illness duration: 17,4 years<br>Onset: 23,4 years<br>Education: 11,7 years<br>IQ: n.i.<br>Diagnosis: schizophrenia<br>Baseline PANSS: 64,1<br>Daily drug dose (CPZeq): 490,8 mg/day	N=23, no dropouts	8	Computer-assisted remediation, 40 sessions total of 40min each, N=12	Treatment as usual, N=11	61	Global Cognition<br>Processing Speed<br>Working Memory<br>Executive Functions<br>Global Symptoms<br>Positive Symptoms<br>Negative Symptoms
Farreny 2012	Spain, single center<br><br>Outpatients	Gender: 32% female<br>Age: 40,6 years<br>Illness duration: 17,5 years<br>Onset: 23,1 years<br>Education: n.i.<br>IQ: n.i.<br>Diagnosis: schizophrenia (89%), schizoaffective disorder<br>Baseline PANSS: 65<br>Daily drug dose (CPZeq): 475 mg/day	N=62, 14,5% attrition rate	17	Problem solving and Cognitive flexibility Training (REPYFLEC), 2 sessions/week of 1h each, N=34	Leisure activities, N=28	76	Global Cognition<br>Working Memory<br>Verbal Memory<br>Executive Functions<br>Global Functioning<br>Global Symptoms<br>Negative Symptoms
Favrod 2014	Switzerland, multi-center<br><br>Outpatients<br><br>Chronic, with active positive symptoms	Gender: 34,6% female<br>Age: 36,7 years<br>Illness duration, onset and education: n.i.<br>IQ: n.i.<br>Diagnosis: non-affective psychosis (83% schizophrenia)<br>Baseline PANSS: n.i.<br>Daily drug dose (CPZeq): 400,5 mg/day	N=52, 8% attrition rate	8	Metacognitive training (MCT), 1h/week, N=26	Multidisciplinary treatment as usual including psychoeducation, N=26	69	Positive Symptoms
Fernandez-Gonzalo 2015	Spain, single center<br><br>Outpatients<br><br>Recent onset (<5 years)	Gender: 10% female<br>Age: 30,5 years<br>Illness duration: 2,6 years<br>Onset: 27,9 years<br>Education: 12,1 years<br>IQ: 83,9<br>Diagnosis: schizophrenia	N=53, 21% attrition rate	22	Computer-assisted remediation using NPT-MH, 2 sessions/week of 1h each, N=28	Non-specific computer activities, N=25	40	Global Cognition<br>All cognitive domains<br>Global Functioning<br>Global Symptoms<br>Positive Symptoms<br>Negative Symptoms

© 2021 Vita et al. *JAMA Psychiatry.*

		(81%), schizoaffective disorder<br>Baseline PANSS: 56,4<br>Daily drug dose (CPZeq): n.i.						
Fisher 2015	USA, multi-center<br><br>Outpatients<br><br>Recent onset (<2 years)	Gender: 23,6% female<br>Age: 21,2 years<br>Illness duration: 1,63 years<br>Onset: 19,6 years<br>Education: 12,9 years<br>IQ: 101,7<br>Diagnosis: schizophrenia, schizoaffective disorder<br>Baseline PANSS: 58,8<br>Daily drug dose (CPZeq): 245,9 mg/day	N=121, 29% attrition rate	8	Computer-assisted remediation using Posit Science Auditory training, 5 sessions/week of 1h each, N=63	Computer games, N=58	68	Global Cognition<br>Processing Speed<br>Working Memory<br>Verbal Memory<br>Visual Memory<br>Executive Functions<br>Global Functioning<br>Global Symptoms
Fisher 2009-2016	USA, single center<br><br>Outpatients	Gender: 27,6% female<br>Age: 41,9 years<br>Illness duration and onset: n.i.<br>Education: 13,3 years<br>IQ: 102,3<br>Diagnosis: schizophrenia<br>Baseline PANSS: 73,7<br>Daily drug dose (CPZeq): 370,6 mg/day	N=87, 15% attrition rate	10	Computer-assisted remediation using Posit Science Auditory training, 5 sessions/week of 1h each, N=46	Computer games, N=41	70	Global Cognition<br>Processing Speed<br>Working Memory<br>Verbal Memory<br>Visual Memory<br>Executive Functions<br>Global Functioning<br>Global Symptoms
Fiszdon 2016	USA, single center<br><br>Outpatients	Gender: 26,7% female<br>Age: 47,8 years<br>Illness duration and onset: n.i.<br>Education: 12,4 years<br>IQ: 93,6<br>Diagnosis: psychotic disorder (81% schizophrenia, affective psychosis included)<br>Baseline PANSS: 52,7<br>Daily drug dose (CPZeq): n.i.	N=75, 17% attrition rate	9	Computer-assisted remediation using PSS CogRehab tasks, 5 sessions/week of 1h hour each, N=50	Treatment as usual, N=25	39	Global Cognition<br>Attention/Vigilance<br>Processing Speed<br>Working Memory<br>Verbal Memory<br>Visual Memory<br>Executive Functions<br>Global Functioning<br>Global Symptoms<br>Positive Symptoms<br>Negative Symptoms
Galderisi 2010	Italy, multi-center<br><br>Outpatients	Gender: 33,3% female<br>Age: 39,8 years<br>Illness duration: 15 years	N=60, 37% attrition rate	26	Social Skills and Neurocognitive Individualized Training (SSANIT), 2 sessions/week of 1h each, N=30	Structured leisure activities, N=30	63	Global Functioning

© 2021 Vita et al. *JAMA Psychiatry.*

		Onset: 24,6 years<br>Education: 10,3 years<br>IQ: n.i.<br>Diagnosis: schizophrenia, schizoaffective disorder<br>Baseline PANSS: n.i.<br>Daily drug dose (CPZeq): 506,5 mg/day						
Garcia 2003	Spain, single center<br><br>Outpatients	Gender: 30% female<br>Age: 38,8 years<br>Illness duration: 18,2 years<br>Onset: 20,6 years<br>Education: n.i.<br>IQ: 74,8<br>Diagnosis: schizophrenia<br>Baseline PANSS: 52,1<br>Daily drug dose (CPZeq): n.i.	N=23, 13% attrition rate	12	Integrated Psychological Therapy (IPT) Social perception subprogram, 2 sessions/week of 30-60min each, N=13	Treatment as usual, N=10	27	Global Cognition<br>Attention/Vigilance<br>Global Functioning<br>Global Symptoms<br>Positive Symptoms<br>Negative Symptoms
Garcia-Fernandez 2019	Spain, single center<br><br>Outpatients<br><br>Recent onset (<1 year)	Gender: 31,4% female<br>Age: 25,5 years<br>Illness duration and onset: n.i.<br>Education: 13,4 years<br>IQ: n.i.<br>Diagnosis: schizophrenia<br>Baseline PANSS: 108,8<br>Daily drug dose (CPZeq): 1067,6 mg/day	N=110, 22% attrition rate	12	Computer-assisted remediation using Rehacom, 2 sessions/week of 1h each, N=54	Computer activities, N=56	73	Global Cognition<br>All cognitive domains<br>Global Functioning
Garrido 2013	Spain, single center<br><br>Outpatients	Gender: 26,8% female<br>Age: 33,3 years<br>Illness duration: 11,4 years<br>Onset: 21,9 years<br>Education: 9,8 years<br>IQ: n.i.<br>Diagnosis: schizophrenia<br>Baseline PANSS: 55,9<br>Daily drug dose (CPZeq): 317 mg/day	N=67, 27% attrition rate	26	Computer-assisted remediation, 2 sessions/week of 1h each, N=38	Video watching, N=29	70	Global Cognition<br>Attention/Vigilance<br>Processing Speed<br>Working Memory<br>Verbal Memory<br>Executive Functions<br>Global Functioning
Gawęda 2015	Poland, single center<br><br>Outpatients	Gender: 50% female<br>Age: 51 years<br>Illness duration: 21,8 years	N=50, 12% attrition rate	4	Metacognitive Training (MCT), 2 sessions/week of 45-60min each, N=26	Treatment as usual (intensive daily rehabilitation including psychoeducation), N=24	57	Global Cognition<br>Processing Speed<br>Working Memory<br>Social Cognition<br>Positive Symptoms

© 2021 Vita et al. *JAMA Psychiatry.*

		Onset: 28,8 years<br>Education: n.i.<br>IQ: n.i.<br>Diagnosis: schizophrenia<br>Baseline PANSS: n.i.<br>Daily drug dose (CPZeq): 488 mg/day						
Gharaeipour 2012	Iran, single center<br><br>Inpatients	Gender: 29% female<br>Age: 28,7 years<br>Illness duration: 15,3 years<br>Onset: n.i.<br>Education: 10,7 years<br>IQ: n.i.<br>Diagnosis: schizophrenia<br>Baseline PANSS: n.i.<br>Daily drug dose (CPZeq): n.i.	N=42, no dropouts	9	Own program, 5 sessions/week of 1h each + 1 group discussion/week, N=21	Group supportive therapy including psychoeducation elements, N=21	65	Global Cognition<br>Processing Speed<br>Working Memory<br>Verbal Memory<br>Visual Memory<br>Executive Functions<br>Positive Symptoms<br>Negative Symptoms
Gohar 2013	Egypt, single center<br><br>Outpatients	Gender: 19% female<br>Age: 31,9 years<br>Illness duration: 10,2 years<br>Onset: 22 years<br>Education: 12,4 years<br>IQ: n.i.<br>Diagnosis: schizophrenia, schizoaffective disorder<br>Baseline PANSS: 83<br>Daily drug dose (CPZeq): n.i.	N=42, no dropouts	8	Social Cognitive Skills Training (SCST), 2 sessions (week of 1h each, N=22	UCLA Skills Training, N=20	55	Global Cognition<br>Social Cognition<br>Global Symptoms<br>Positive Symptoms<br>Negative Symptoms
Gomar 2015	Spain, multi-center<br><br>Inpatients	Gender: 31,5% female<br>Age: 46,1 years<br>Illness duration: 23,4 years<br>Onset: 22,7 years<br>Education: 9,7 years<br>IQ: 86,1<br>Diagnosis: schizophrenia, schizoaffective disorder<br>Baseline PANSS: 75,9<br>Daily drug dose (CPZeq): 663,7 mg/day	N=130, 18% attrition rate	26	Computer-assisted remediation using FesKits, 2 sessions/week of 45min each, N=43	Computerized typing program, N=44<br>Treatment as usual, N=43	92	Global Cognition<br>Processing Speed<br>Working Memory<br>Verbal Memory<br>Visual Memory<br>Executive Functions<br>Global Functioning
Gordon 2018	Australia, multi-center<br><br>Outpatients	Gender: n.i.<br>Age: 35,5 years	N=36, 8% attrition rate	10	Social Cognition and Interaction Training (SCIT), 2 sessions/week of 1h each, N=21	Treatment as usual, Waiting list, N=15	59	Global Cognition<br>Social Cognition<br>Global Functioning

© 2021 Vita et al. *JAMA Psychiatry.*

		Illness duration: 9,8 years<br>Onset: 25,7 years<br>Education: n.i.<br>IQ: n.i.<br>Diagnosis: non-affective psychosis (72% schizophrenia)<br>Baseline PANSS: n.i.<br>Daily drug dose (CPZeq): n.i.						
Greig 2007	USA, single center<br><br>Outpatients	Gender: 46,8% female<br>Age: 40,4 years<br>Illness duration: 16,3 years<br>Onset: 24,1 years<br>Education: 12,7 years<br>IQ: 87,1<br>Diagnosis: schizophrenia (66%), schizoaffective disorder<br>Baseline PANSS: 79,5<br>Daily drug dose (CPZeq): n.i.	N=77, 19,5% attrition rate	52	Neurocognitive Enhancement Therapy (NET), 11h/week, + Vocational rehabilitation, N=40	Vocational rehabilitation, N=37	60	Global Cognition<br>Working Memory<br>Verbal Memory<br>Visual Memory<br>Executive Functions<br>Social Cognition
Habel 2010	Germany, single center<br><br>In- and outpatients	Gender: 100% male<br>Age: 32,6 years<br>Illness duration and onset and education: n.i.<br>IQ: 111,4<br>Diagnosis: schizophrenia<br>Baseline PANSS: 62,8<br>Daily drug dose (CPZeq): n.i.	N=20, no dropouts	6	Training of Affect Recognition (TAR), 12 sessions total of 45 min each, N=10	Treatment as usual, N=10	27	Global Cognition<br>Social Cognition
Hadas-Lidor 2001	Israel, single center<br><br>Outpatients	Gender: 39,6% female<br>Age: 36 years<br>Illness duration, onset and education: n.i.<br>IQ: n.i.<br>Diagnosis: schizophrenia<br>Baseline PANSS: n.i.<br>Daily drug dose (CPZeq): n.i.	N=72, 19,5% attrition rate	52	Instrumental Enrichment program, 2-3 sessions/week of 1h each, N=36	Occupational therapy, treatment as usual consisting of daily rehabilitation program, N=36	54	Global Cognition<br>Working Memory<br>Verbal Memory<br>Visual Memory
Hegde 2012	India, single center<br><br>Outpatients	Gender: 17% female<br>Age: 29,3 years	N=45, 31% attrition rate	9	Home-based program, flexible schedule over time, N=22	Treatment as usual including psychoeducation, N=23	35	Global Cognition<br>Attention/Vigilance<br>Executive Functions

© 2021 Vita et al. *JAMA Psychiatry.*

	First-episode and recent onset (<2 years)	Illness duration and onset: n.i.<br>Education: 12,8 years<br>IQ: n.i.<br>Diagnosis: schizophrenia<br>Baseline PANSS: 89,7<br>Daily drug dose (CPZeq): 204 mg/day						Negative Symptoms
Hermanutz 1991	Germany, single center<br><br>Inpatients	Gender: n.i.<br>Age: 31,3 years<br>Illness duration and onset: n.i.<br>Education: 11 years<br>IQ: n.i.<br>Diagnosis: schizophrenia<br>Baseline PANSS: 90,9<br>Daily drug dose (CPZeq): n.i.	N=30, no dropouts	4	Computer-assisted Attention training, unspecified schedule, N=10, or<br>Integrated Psychological Therapy (IPT) selected subprograms, N=10	Treatment as usual, N=10	65	Global Cognition<br>Attention/Vigilance<br>Executive Functions<br>Global Functioning<br>Global Symptoms<br>Positive Symptoms<br>Negative Symptoms
Hodge 2010	Australia, multi-center<br><br>In- and outpatients	Gender: 40,6% female<br>Age: 31,3 years<br>Illness duration and onset: n.i.<br>Education: 11 years<br>IQ: 93,7<br>Diagnosis: schizophrenia<br>Baseline PANSS: 69,2<br>Daily drug dose (CPZeq): 649,9 mg/day	N=69, 48% attrition rate	10	Neuropsychological and Educational Approach to Remediation (NEAR), 2 sessions/week of 1h each, N=36	Treatment as usual, Waiting list N=33	60	Global Cognition<br>Attention/Vigilance<br>Processing Speed<br>Working Memory<br>Verbal Memory<br>Visual Memory<br>Executive Functions
Hogarty 2004	USA, single center<br><br>Outpatients	Gender: 41% female<br>Age: 37,2 years<br>Illness duration: 15,7 years<br>Onset: 21,5 years<br>Education: n.i.<br>IQ: n.i.<br>Diagnosis: schizophrenia (70%), schizoaffective disorder<br>Baseline PANSS: n.i.<br>Daily drug dose (CPZeq): n.i.	N=121, 11,5% attrition rate	104	Cognitive Enhancement Therapy (CET), almost 2,5h/week, + Enriched supportive therapy, N=67	Enriched supportive therapy (including skills training and psychoeducation), N=54	63	Global Cognition<br>Processing Speed<br>Working Memory<br>Verbal Memory<br>Executive Functions<br>Global Functioning
Hooker 2012	USA, single center<br><br>Outpatients	Gender: 18% female<br>Age: 46,1 years<br>Illness duration: 24,3 years<br>Onset: 21,8 years	N=22, no dropouts	10	Computer-assisted remediation using Posit Science Auditory training + Social cognition Training, 5 sessions/week of	Computer games, N=11	66	Global Cognition<br>Social Cognition<br>Global Functioning

© 2021 Vita et al. *JAMA Psychiatry.*

		Education: 13,3 years<br>IQ: 100,9<br>Diagnosis: schizophrenia (59%), schizoaffective disorder<br>Baseline PANSS: 72,2<br>Daily drug dose (CPZeq): 312 mg/day			65-75min each, N=11			
Horan 2009	USA, single center<br><br>Outpatients	Gender: 6% female<br>Age: 48,2 years<br>Illness duration and onset: n.i.<br>Education: 12,3 years<br>IQ: n.i.<br>Diagnosis: schizophrenia, schizoaffective disorder<br>Baseline PANSS: n.i.<br>Daily drug dose (CPZeq): n.i.	N=34, 9% attrition rate	6	Social Cognitive Skills Training (SCST), 2 sessions/week, N=17	UCLA Illness Self-Management and Relapse Prevention Training, N=17	39	Global Cognition<br>Social Cognition<br>Global Symptoms<br>Positive Symptoms<br>Negative Symptoms
Horan 2011	USA, single center<br><br>Outpatients	Gender: 11,8% female<br>Age: 48,1 years<br>Illness duration: 25,6 years<br>Onset: 22,5 years<br>Education: 12,9 years<br>IQ: n.i.<br>Diagnosis: non-affective psychosis (71% schizophrenia)<br>Baseline PANSS: 55,9<br>Daily drug dose (CPZeq): n.i.	N=85, 22% attrition rate	12	Computer-assisted neurocognitive training using Posit Science Aristotle (NR), N=24<br>Social Cognitive Skills Training (SCST), N=19<br>Hybrid training (SCST+NR), N=21<br>In all cases: 2 sessions/week of 1h each	UCLA Skills Training, N=21	69	Global Cognition<br>Social Cognition<br>Global Functioning<br>Global Symptoms<br>Positive Symptoms<br>Negative Symptoms
Horan 2018	USA, single center<br><br>Outpatients	Gender: 30,2% female<br>Age: 47,3 years<br>Illness duration: 25,6 years<br>Onset: 21,7 years<br>Education: 12,4 years<br>IQ: n.i.<br>Diagnosis: non-affective psychotic disorder<br>Baseline PANSS: 50,9<br>Daily drug dose (CPZeq): n.i.	N=139, 6,5% attrition rate	12	Social Cognitive Skills Training (SCST) either in vivo, N=41<br>or clinic, N=49<br>In both cases: flexible schedule, 2 to 3 sessions/week of 1h each.	UCLA Illness Management Skills Training, N=49	85	Global Cognition<br>Social Cognition<br>Global Functioning<br>Global Symptoms<br>Positive Symptoms<br>Negative Symptoms

© 2021 Vita et al. *JAMA Psychiatry.*

Iwata 2017	Japan, multi-center<br><br>Outpatients	Gender: 75% female<br>Age: 34,4 years<br>Illness duration: 11,9 years<br>Onset: 22,5 years<br>Education: n.i.<br>IQ: 97,9<br>Diagnosis: schizophrenia<br>Baseline PANSS: 58,9<br>Daily drug dose (CPZeq): 673,3 mg/day	N=60, no dropouts	12	Thinking Skills for Work, 2 sessions/week of 45-60 min each +1-group session/week, N=29	Treatment as usual including social skills training and psychoeducation, waiting list, N=31	67	Global Cognition<br>Processing Speed<br>Working Memory<br>Verbal Memory<br>Executive Functions<br>Global Functioning<br>Global Symptoms<br>Positive Symptoms<br>Negative Symptoms
Jahshan 2019	USA, single center<br><br>In- and outpatients	Gender: 22,2% female<br>Age: 51,3 years<br>Illness duration: 29,7 years<br>Onset: 21,6 years<br>Education: 12,8 years<br>IQ: n.i.<br>Diagnosis: schizophrenia, schizoaffective disorder<br>Baseline PANSS: 53,1<br>Daily drug dose (CPZeq): n.i.	N=99, 16% attrition rate	12	Computer-assisted remediation using either Brain Fitness, N=39 or Cogpack, N=40<br>In both cases: 3 sessions/week	Computer games, N=20	69	Global Cognition<br>All cognitive domains<br>Global Functioning
Kanie 2019	Japan, multi-center<br><br>Outpatients	Gender: 42,2% female<br>Age: 36,5 years<br>Illness duration: 14 years<br>Onset: 22,5 years<br>Education: 13,5 years<br>IQ: 101,4<br>Diagnosis: schizophrenia, schizoaffective disorder<br>Baseline PANSS: 64,3<br>Daily drug dose (CPZeq): 632,5 mg/day	N=72, 15% attrition rate	24	Social Cognition and Interaction Training (SCIT), 20-24h/week, N=36	Multidisciplinary treatment as usual, N=36	75	Global Cognition<br>Social Cognition<br>Global Functioning<br>Global Symptoms<br>Positive Symptoms<br>Negative Symptoms
Kantrowitz 2016	USA, multi-center<br><br>Outpatients	Gender: 35% female<br>Age: 37,7 years<br>Illness duration, onset and education: n.i.<br>IQ: n.i.<br>Diagnosis: schizophrenia, schizoaffective disorder<br>Baseline PANSS: 70,3	N=133, 34% attrition rate	15	Computer-assisted remediation using Posit Science Brain Fitness + bridging groups using Neuropsychological and Educational Approach to Remediation (NEAR), 2 sessions/week, N=63	Videogames, N=70	74	Global Cognition<br>All cognitive domains<br>Global Functioning<br>Global Symptoms<br>Positive Symptoms<br>Negative Symptoms

© 2021 Vita et al. *JAMA Psychiatry.*

		Daily drug dose (CPZeq): 253 mg/day; stabilized on lurasidone at study start						
Katsumi 2019	Japan, single center<br><br>Outpatients	Gender: 40,9% female<br>Age: 37,8 years<br>Illness duration: 13,9 years<br>Onset: 23,9 years<br>Education: 12 years<br>IQ: 90,7<br>Diagnosis: schizophrenia, schizoaffective disorder<br>Baseline PANSS: 66,6<br>Daily drug dose (CPZeq): 664,4 mg/day	N=44, no dropouts	5	Neuropsychological and Educational Approach to Remediation (NEAR), 4 sessions/week, of 40-60min each, N=22	Treatment as usual consisting of an integrative rehabilitation program, N=22	42	Global Cognition<br>Processing Speed<br>Working Memory<br>Verbal Memory<br>Executive Functions<br>Global Functioning<br>Global Symptoms<br>Positive Symptoms<br>Negative Symptoms
Keefe 2012	USA multi-center<br><br>Outpatients	Gender: 26,4% female<br>Age: 37 years<br>Illness duration and onset: n.i.<br>Education: 13,5 years<br>IQ: n.i.<br>Diagnosis: schizophrenia<br>Baseline PANSS: 56,2<br>Daily drug dose (CPZeq): n.i.	N=53, 11% attrition rate	8	Computer-assisted remediation using Posit Science Brain Fitness + bridging groups using NEAR, 40h/week (flexible schedule), N=27	Computer games and Healthy lifestyle groups, N=26	82	Global Cognition<br>All cognitive domains<br>Global Functioning<br>Global Symptoms
Kidd 2014	Canada, single center<br><br>Outpatients, university students	Gender: 54% female<br>Age: 34,2 years<br>Illness duration: 6,9 years<br>Onset: 27,3 years<br>Education: 12,5 years<br>IQ: n.i.<br>Diagnosis: any psychotic disorder (affective psychosis included, 56% schizophrenia)<br>Baseline PANSS: 65,1<br>Daily drug dose (CPZeq): n.i.	N=37, 16% attrition rate	20	Computer-assisted remediation using Cogpack, 2 sessions/week of 50min each + Group discussions 50min/week + Supported education program, N=19	Supported education program, N=18	65	Global Cognition<br>Attention/Vigilance<br>Processing Speed<br>Working Memory<br>Verbal Memory<br>Executive Functions<br>Global Symptoms<br>Positive Symptoms<br>Negative Symptoms
Klingberg 2011	Germany, multi-center<br><br>Outpatients	Gender: 43,9% female<br>Age: 36,9 years<br>Illness duration: 12,5 years<br>Onset: 24,4 years	N=198, 15% attrition rate	39	Computer-assisted remediation using Cogpack, 20 sessions total, of almost 50min each, N=99	Cognitive Behavioral Therapy (CBT), N=99	91	Global Functioning<br>Global Symptoms<br>Positive Symptoms<br>Negative Symptoms

© 2021 Vita et al. *JAMA Psychiatry.*

		Education: n.i.<br>IQ: 107,6<br>Diagnosis: schizophrenia<br>Baseline PANSS: 59,4<br>Daily drug dose (CPZeq): 543 mg/day						
Kukla 2018	USA, single center<br><br>Outpatients	Gender: 8% female<br>Age: 48,5 years<br>Illness duration and onset: n.i.<br>Education: 12,9 years<br>IQ: n.i.<br>Diagnosis: schizophrenia (70%), schizoaffective disorder<br>Baseline PANSS: 75,1<br>Daily drug dose (CPZeq): n.i.	N=50, 14% attrition rate	26	Computer-assisted remediation using Posit Science Fitness and Insight, 1 session/week, + Cognitive Behavioral Therapy (CBT), N=25	Cognitive Behavioral Therapy (CBT), N=25	67	Global Cognition<br>All cognitive domains<br>Global Functioning
Kumar 2010	India, single center<br><br>Inpatients<br><br>Acute phase	Gender: 100% male<br>Age: 32,8 years<br>Illness duration: 7,1 years<br>Onset: 25,7 years<br>Education: 9,3 years<br>IQ: n.i.<br>Diagnosis: schizophrenia<br>Baseline PANSS: 98,2<br>Daily drug dose (CPZeq): n.i.	N=16, no dropouts	4	Metacognitive training (MCT), 2 sessions/week of 45-60min each, N=8	Treatment as usual including psychoeducation, N=8	37	Global Symptoms<br>Positive Symptoms<br>Negative Symptoms
Kurtz 2007	USA, multi-center<br><br>Outpatients	Gender: 33,3% female<br>Age: 35 years<br>Illness duration: 10,5 years<br>Onset: 24,5 years<br>Education: 13,1 years<br>IQ: n.i.<br>Diagnosis: schizophrenia, schizoaffective disorder<br>Baseline PANSS: n.i.<br>Daily drug dose (CPZeq): n.i.	N=42, no dropouts	52	Computer-assisted remediation, 100h total, N=23	Computer skills training, N=19	63	Global Cognition<br>Processing Speed<br>Working Memory<br>Verbal Memory<br>Visual Memory<br>Executive Functions
Kurtz 2015	USA, single center<br><br>Outpatients	Gender: 27% female<br>Age: 36,6 years<br>Illness duration: 12,6 years	N=64, 12,5% attrition rate	26	Computer-assisted own program + Social Skills Training, 50h total, N=32	Computer games + Social Skills Training, N=32	70	Global Cognition<br>Attention/Vigilance<br>Working Memory<br>Verbal Memory<br>Executive Functions

© 2021 Vita et al. *JAMA Psychiatry.*

		Onset: 24,1 years<br>Education: 12,1 years<br>IQ: n.i.<br>Diagnosis: schizophrenia, schizoaffective disorder<br>Baseline PANSS: n.i.<br>Daily drug dose (CPZeq): n.i.						Global Functioning
Lado-Colesido 2019	Spain, multi-center<br><br>Outpatients	Gender: 45% female<br>Age: 40,9 years<br>Illness duration, onset and education: n.i.<br>IQ: 97,9<br>Diagnosis: schizophrenia (90%), schizoaffective disorder<br>Baseline PANSS: 54,4<br>Daily drug dose (CPZeq): 1008,3	N=53, attrition rate 5,7%	4	Voices program (from e-Motional training), 2 sessions/week of 30min each, N=27	Treatment as usual (including psychotherapy), N=26	63	Global Cognition<br>Social Cognition
Lee 2013	South Korea, single center<br><br>Inpatients	Gender: 45% female<br>Age: 43,5 years<br>Illness duration: 17,6 years<br>Onset: 25,3 years<br>Education: 12,8 years<br>IQ: 99,7<br>Diagnosis: schizophrenia<br>Baseline PANSS: 64<br>Daily drug dose (CPZeq): 316,8 mg/day	N=66, 9% attrition rate	13	Computer-assisted program using Cog-Trainer, 20 sessions total, N=33	Treatment as usual (including social skills training and psychoeducation), N=33	68	Global Cognition<br>Attention/Vigilance<br>Working Memory<br>Global Functioning<br>Global Symptoms<br>Positive Symptoms<br>Negative Symptoms
Lindenmayer 2008	USA, single center<br><br>Inpatients	Gender: 11% female<br>Age: 43,5 years<br>Illness duration: 25,1 years<br>Onset: 18,4 years<br>Education: 10,6 years<br>IQ: n.i.<br>Diagnosis: schizophrenia, schizoaffective disorder<br>Baseline PANSS: n.i.<br>Daily drug dose (CPZeq): n.i.	N=85, 15% attrition rate	12	Computer-assisted remediation using Cogpack, 2-3 computerized sessions/week of 45min each, + 1h group discussions/week + Work program, N=45	Computer games + Work program, N=40	57	Global Cognition<br>Processing Speed<br>Working Memory<br>Verbal Memory<br>Executive Functions
Lu 2012	China, single center	Gender: 38,9% female	N=126, 3%	12	Cognitive Remediation Therapy (CRT), 5	Treatment as usual, N=63	43	Global Cognition<br>Executive Functions

© 2021 Vita et al. *JAMA Psychiatry.*

	Inpatients	Age: 37,5 years<br>Illness duration: 23,5 years<br>Onset: 14 years<br>Education: 10,5 years<br>IQ: n.i.<br>Diagnosis: schizophrenia<br>Baseline PANSS: n.i.<br>Daily drug dose (CPZeq): 745,5 mg/day, the majority receiving clozapine	attrition rate		sessions/week of 45min each, N=63			Global Functioning
Mahncke 2019	USA, multi-center<br><br>Outpatients	Gender: 19% female<br>Age: 42.9 years<br>Illness duration and onset: n.i.<br>Education: 12.7 years<br>IQ: n.i.<br>Diagnosis: schizophrenia<br>Baseline PANSS: n.i.<br>Daily drug dose (CPZeq): n.i.	N=150, 35% attrition rate	26	Computer-assisted training using a selection of Posit Science tasks and novel exercises, 5 sessions/week of 1h each, N=75	Computer games, N=75	83	Global Cognition<br>Global Functioning
Mak 2013	Poland, single center<br><br>Outpatients	Gender: 54% female<br>Age: 36,5 years<br>Illness duration: 10 years<br>Onset: 26,5 years<br>Education: n.i.<br>IQ: n.i.<br>Diagnosis: schizophrenia<br>Baseline PANSS: 58<br>Daily drug dose (CPZeq): n.i.	N=81, dropouts n.i.	8	Computer-assisted remediation using Rehacom, 2 sessions/week of 40min each, N=41	Treatment as usual, N=40	38	Global Cognition<br>Processing Speed<br>Working Memory<br>Executive Functions
Man 2012	Hong Kong, single center<br><br>Inpatients	Gender: 36,6% female<br>Age: 36,9 years<br>Illness duration, onset and education: n.i.<br>IQ: 89,1<br>Diagnosis: schizophrenia<br>Baseline PANSS: n.i.<br>Daily drug dose (CPZeq): n.i.	N=90, 11% attrition rate	n.i.	Errorless learning training, either computer-assisted, N=30, or therapist-assisted, 12 sessions, N=26	Treatment as usual, N=34	57	Global Cognition<br>Working Memory<br>Verbal Memory<br>Executive Functions
Maroño Souto 2018	Spain, multi-center<br><br>Outpatients	Gender: 13% female<br>Age: 39,2 years	N=61, 2% attrition rate	12	e-Motional training, 1h/week, N=30	Treatment as usual including psychotherapy, N=31	60	Global Cognition<br>Social Cognition<br>Positive Symptoms

© 2021 Vita et al. *JAMA Psychiatry.*

		Illness duration, onset and education: n.i.<br>IQ: 98,5<br>Diagnosis: schizophrenia<br>Baseline PANSS: n.i.<br>Daily drug dose (CPZeq): 600,4 mg/day						Negative Symptoms
Matsuda 2018	Japan, multi-center<br><br>Outpatients	Gender: 43,5% female<br>Age: 37,1 years<br>Illness duration: 13,5 years<br>Onset: 23,6 years<br>Education: 13,4 years<br>IQ: 101,5<br>Diagnosis: schizophrenia<br>Baseline PANSS: n.i.<br>Daily drug dose (CPZeq): n.i.	N=62, 5% attrition rate	12	Japanese Cognitive Rehabilitation Program for Schizophrenia (JCORES), 3 sessions/week of 1h each, N=31	Treatment as usual, N=31	69	Global Cognition<br>Processing Speed<br>Working Memory<br>Verbal Memory<br>Executive Functions<br>Global Functioning<br>Global Symptoms<br>Positive Symptoms<br>Negative Symptoms
Matsui 2009	Japan, single center<br><br>Outpatients	Gender: n.i.<br>Age: 32,3 years<br>Illness duration: 8,3 years<br>Onset: 24 years<br>Education: 12,7 years<br>IQ: 99,9<br>Diagnosis: schizophrenia<br>Baseline PANSS: n.i.<br>Daily drug dose (CPZeq): 182,5 mg/day	N=20, no dropouts	12	Own program, 1session/week of 40min, N=11	Treatment as usual (maintenance medication only), N=9	47	Global Cognition<br>Working Memory<br>Executive Functions<br>Social Cognition<br>Positive Symptoms<br>Negative Symptoms
McGurk 2005	USA, multi-center<br><br>Outpatients	Gender: 45,5% female<br>Age: 35,6 years<br>Illness duration: 12,9 years<br>Onset: 22,7 years<br>Education: 11,3 years<br>IQ: n.i.<br>Diagnosis: any psychotic disorder (affective psychosis included, 73% schizophrenia)<br>Baseline PANSS: n.i.<br>Daily drug dose (CPZeq): n.i.	N=48, dropouts n.i.	12	Computer-assisted remediation using Cogpack, 2-3 sessions/week of 1h each, + Supported employment, N=25	Treatment as usual consisting of supported employment, N=23	62	Global Cognition<br>Processing Speed<br>Working Memory<br>Verbal Memory<br>Executive Functions<br>Global Symptoms<br>Positive Symptoms<br>Negative Symptoms
McGurk 2016	USA, single center	Gender: 30% female	N=54, 5,5%	12	Thinking Skills for Work + Vocational	Enhanced Vocational	79	Global Cognition<br>Processing Speed

© 2021 Vita et al. *JAMA Psychiatry.*

	Outpatients, non-responsive to vocational rehabilitation	Age: 37,7 years<br>Illness duration, onset and education: n.i.<br>IQ: n.i.<br>Diagnosis: psychotic disorder (affective included, 81% schizophrenia)<br>Baseline PANSS: 70,3<br>Daily drug dose (CPZeq): n.i.	attrition rate		Rehabilitation, 1-2 sessions/week of 45-60min each, N=28	Rehabilitation, N=26		Working Memory<br>Verbal Memory<br>Executive Functions<br>Global Symptoms<br>Positive Symptoms<br>Negative Symptoms
Medalia 1998	USA, single center<br><br>Inpatients	Gender: 22,5% female<br>Age: 32,5 years<br>Illness duration and onset: n.i.<br>Education: 10,8 years<br>IQ: n.i.<br>Diagnosis: schizophrenia<br>Baseline PANSS: 61,1<br>Daily drug dose (CPZeq): n.i.	N=54, no dropouts	6	Attention Training, 3 sessions/week of 20min each, N=27	Video watching, N=27	43	Global Cognition<br>Attention/Vigilance<br>Global Symptoms
Medalia 2000	USA, single center<br><br>Inpatients	Gender: 44,4% female<br>Age: 36,3 years<br>Illness duration: 15 years<br>Onset: 21,3 years<br>Education: 10,5 years<br>IQ: n.i.<br>Diagnosis: schizophrenia (75%), schizoaffective disorder<br>Baseline PANSS: n.i.<br>Daily drug dose (CPZeq): n.i.	N=54, dropouts n.i.	5	Memory remediation program, N=18<br>Or Processing Speed remediation program, N=18<br>In both cases: 2 sessions/week of 25min each	Treatment as usual, N=18	54	Global Cognition<br>Verbal Memory<br>Global Functioning
Meichenbaum 1973	Canada, single center<br><br>Outpatients<br><br>50% acute phase	Gender: 100% male<br>Age: 36 years<br>Illness duration, onset and education: n.i.<br>IQ: n.i.<br>Diagnosis: schizophrenia<br>Baseline PANSS: n.i.<br>Daily drug dose (CPZeq): n.i.	N=10, no dropouts	3	Self-instructional training group, own program, 8 sessions of 45 min each, N=5	Yoked practice group, N=5	65	Global Cognition<br>Working Memory<br>Executive Functions<br>Positive Symptoms
Mendella 2015	Canada, single center<br><br>Outpatients	Gender: 25,9% female<br>Age: 24,9 years	N=31, 13% attrition rate	12	Compensatory Cognitive Training (CCT), 2	Treatment as usual consisting of OnTrack (multidisciplina	52	Global Cognition<br>All cognitive domains

© 2021 Vita et al. *JAMA Psychiatry.*

	First-episode	Illness duration and onset: n.i.<br>Education: 13,2 years<br>IQ: 99,4<br>Diagnosis: first-episode schizophrenia<br>Baseline PANSS: n.i.<br>Daily drug dose (CPZeq): n.i.			sessions/week of 1h each, N=16	ry program for first-episode patients), N=15		Global Functioning<br>Positive Symptoms<br>Negative Symptoms
Morimoto 2018	Japan, single center<br><br>Outpatients	Gender: 39% female<br>Age: 36,7 years<br>Illness duration: 13 years<br>Onset: 23,8 years<br>Education: 13,7 years<br>IQ: n.i.<br>Diagnosis: schizophrenia<br>Baseline PANSS: 75,6<br>Daily drug dose (CPZeq): 556,4 mg/day	N=31, no dropout s	12	Vocational and Cognitive Abilities Training (VCAT-J), 2 sessions/week of 1h each, N=16	Treatment as usual, waiting list, N=15	64	Global Cognition<br>Processing Speed<br>Working Memory<br>Verbal Memory<br>Executive Functions<br>Global Functioning<br>Global Symptoms<br>Positive Symptoms<br>Negative Symptoms
Moritz 2011	Germany, single center<br><br>In- and outpatients<br><br>In remission at study start	Gender: 22,2% female<br>Age: 32,8 years<br>Illness duration and onset: n.i.<br>Education: 10,6 years<br>IQ: 100,8<br>Diagnosis: non-affective psychosis<br>Baseline PANSS: 48,7<br>Daily drug dose (CPZeq): n.i.	N=37, no dropout s	8	Metacognitive Training (MCT), 1 session/week of 45-60min, N=18	Treatment as usual, Waiting List, N=18	67	Global Cognition<br>Verbal Memory<br>Visual Memory<br>Positive Symptoms
Moritz 2015	Germany, multi-center<br><br>In- and outpatients	Gender: 63,3% female<br>Age: 39,3 years<br>Illness duration, onset and education: n.i.<br>IQ: n.i.<br>Diagnosis: schizophrenia, schizoaffectiv e disorder<br>Baseline PANSS: n.i.<br>Daily drug dose (CPZeq): n.i.	N=90, 23% attrition rate	6	Home-based computer-assisted remediation using MyBrainTraining, flexible schedule, N=30, or<br>Same program but metacognition-augmented, N=30	Treatment as usual, Waiting list, N=30	50	Negative Symptoms
Müller 2015	Germany, Austria and Switzerland, multi-center<br><br>Outpatients	Gender: 31% female<br>Age: 34,2 years<br>Illness duration: 10,1 years	N=156, 10% attrition rate	15	Integrated Neurocognitive Therapy (INT), 2 sessions/week of 90 min each, N=81	Treatment as usual, N=75	87	Global Cognition<br>All cognitive domains<br>Global Functioning<br>Global Symptoms

© 2021 Vita et al. *JAMA Psychiatry.*

		Onset: 24,2 years<br>Education: 11 years<br>IQ: 104<br>Diagnosis: schizophrenia, schizoaffective disorder<br>Baseline PANSS: n.i.<br>Daily drug dose (CPZeq): 438,5 mg/day						Positive Symptoms<br>Negative Symptoms
Müller 2017	Switzerland, Single center<br><br>Outpatients<br><br>Severe negative symptoms	Gender: 22,9% female<br>Age: 35,5 years<br>Illness duration: 10,8 years<br>Onset: 24,7 years<br>Education: 10,8 years<br>IQ: 101,4<br>Diagnosis: schizophrenia, schizoaffective disorder<br>Baseline PANSS: 78<br>Daily drug dose (CPZeq): 405,4 mg/day	N=61, 10% attrition rate	15	Integrated Neurocognitive Therapy (INT), 2 sessions/week of 90 min each, N=28	Treatment as usual, N=33	71	Global Cognition<br>Attention<br>Processing Speed<br>Verbal Memory<br>Executive Functions<br>Social Cognition<br>Global Functioning<br>Positive Symptoms<br>Negative Symptoms
O'Reilly 2019	Ireland, single center<br><br>Inpatients in a forensic setting (medium to high security)	Gender: 15,4% female<br>Age: 41 years<br>Illness duration, onset and education: n.i.<br>IQ: n.i.<br>Diagnosis: schizophrenia (76%), schizoaffective disorder<br>Baseline PANSS: 51,2<br>Daily drug dose (CPZeq): 488,5 mg/day	N=65, 11% attrition rate	14	Own program for forensic patients, 4 sessions/week, N=32	Treatment as usual, Waiting list, N=33	87	Global Cognition<br>Processing Speed<br>Working Memory<br>Verbal Memory<br>Visual Memory<br>Executive Functions<br>Social Cognition<br>Global Functioning<br>Global Symptoms<br>Positive Symptoms<br>Negative Symptoms
Ochoa 2017	Spain, multi-center<br><br>Outpatients<br><br>Recent onset (< 5 years)	Gender: 30% female<br>Age: 27,6 years<br>Illness duration: 2,3 years<br>Onset: 25,6 years<br>Education: n.i.<br>IQ: n.i.<br>Diagnosis: schizophrenia spectrum disorder<br>Baseline PANSS: 54,3<br>Daily drug dose (CPZeq): 494,3 mg/d, typical and atypical	N=122; 27% attrition rate	8	Metacognitive Training (MCT), 1 session/week, N=65	Psychoeducation, N=57	71	Global Cognition<br>Social Cognition<br>Global Functioning<br>Global Symptoms<br>Positive Symptoms<br>Negative Symptoms

© 2021 Vita et al. *JAMA Psychiatry.*

Ojeda 2012	Spain, single center<br><br>Inpatients<br><br>Study defined as treatment resistant	Gender: 21% female<br>Age: 34,1 years<br>Illness duration: 13 years<br>Onset: 22,4 years<br>Education: 9,2 years<br>IQ: n.i.<br>Diagnosis: schizophrenia<br>Baseline PANSS: 99,3<br>Daily drug dose (CPZeq): 886,7 mg/day	N=93, 10% attrition rate	12	REHACOP, 3 sessions/week of 90 min each, N=47	Occupational group therapy, N= 46	64	Global Cognition<br>Processing Speed<br>Working Memory<br>Verbal Memory<br>Executive Functions<br>Global Functioning<br>Global Symptoms<br>Positive Symptoms<br>Negative Symptoms
Omiya 2016	Japan, single center<br><br>In- and outpatients	Gender: 59% female<br>Age: 41 years<br>Illness duration and onset: n.i.<br>Education: 13,4 years<br>IQ: 79,1<br>Diagnosis: schizophrenia<br>Baseline PANSS: 78,6<br>Daily drug dose (CPZeq): 906,7 mg/day	N=17, no dropouts	26	Frontal/executive Program (FEP-J), 2 sessions/week of 1h each, N=8	Treatment as usual including psychotherapy, N=9	41	Global Cognition<br>Attention/Vigilance<br>Processing Speed<br>Working Memory<br>Verbal Memory<br>Executive Functions<br>Global Functioning<br>Global Symptoms
Østergaard Christensen 2014	Denmark, multi-center<br><br>Inpatients<br><br>First-episode	Gender: 46,2% female<br>Age: 24,9 years<br>Illness duration, onset and education: n.i.<br>IQ: n.i.<br>Diagnosis: first-episode of psychosis (schizophrenia 84%)<br>Baseline PANSS: 54,2<br>Daily drug dose (CPZeq): n.i.	N=117, 16% attrition rate	16	Computer-assisted remediation, 2 sessions/week of 1h each, integrated with OPUS program, N=60	Treatment as usual consisting of a tailored comprehensive psychosocial program (OPUS), N=57	84	Global Cognition<br>All cognitive domains<br>Global Functioning<br>Global Symptoms<br>Positive Symptoms<br>Negative Symptoms
Peña 2016	Spain, single center<br><br>In- and outpatients	Gender: 28% female<br>Age: 39 years<br>Illness duration: 15,8 years<br>Onset: 23,2 years<br>Education: 10,4 years<br>IQ: n.i.<br>Diagnosis: schizophrenia<br>Baseline PANSS: n.i.<br>Daily drug dose (CPZeq): 488,9 mg/day	N=104, 3% attrition rate	17	REHACOP, 3 sessions/week of 90 min each, N=52	Occupational group activities, N=52	76	Global Cognition<br>Social Cognition<br>Global Functioning<br>Global Symptoms<br>Positive Symptoms<br>Negative Symptoms
Penades 2006	Spain, single center<br><br>Outpatients	Gender: 42,5% female<br>Age: 35,1 years	N=40, 17,5% attrition rate	17	Frontal/Executive Program, 2-3 sessions/week of 1h each, N=20	Cognitive Behavioral Therapy for	67	Global Cognition<br>Processing Speed<br>Working Memory

© 2021 Vita et al. *JAMA Psychiatry.*

	Predominantly negative symptoms	Illness duration: 13,8 years<br>Onset: 21,3 years<br>Education: 11,6 years<br>IQ: n.i.<br>Diagnosis: schizophrenia<br>Baseline PANSS: 67<br>Daily drug dose (CPZeq): 361,3 mg/day				psychosis (CBTp), N=20		Verbal Memory<br>Visual Memory<br>Executive Functions<br>Global Functioning<br>Global Symptoms<br>Positive Symptoms<br>Negative Symptoms
Penades 2013	Spain, single center<br><br>Outpatients<br><br>Predominantly negative symptoms	Gender: 22,9% female<br>Age: 37 years<br>Illness duration: 12,9 years<br>Onset: 24 years<br>Education: 11,8 years<br>IQ: n.i.<br>Diagnosis: schizophrenia<br>Baseline PANSS: 64,7<br>Daily drug dose (CPZeq): 269,4 mg/day	N=35, dropouts n.i.	17	Frontal/Executive Program, 2-3 sessions/week of 1h each, N=17	Social Skills Training, N=18	72	Global Cognition<br>Processing Speed<br>Working Memory<br>Verbal Memory<br>Visual Memory<br>Executive Functions
Penades 2018	Spain, single center<br><br>Outpatients	Gender: 31,4% female<br>Age: 40,1 years<br>Illness duration: 15,9 years<br>Onset: 24,2 years<br>Education: n.i.<br>IQ: n.i.<br>Diagnosis: schizophrenia<br>Baseline PANSS: 100,4<br>Daily drug dose (CPZeq): 371,8 mg/day	N=70, 7,1% attrition rate	17	Frontal/Executive Program, 2-3 sessions/week of 1h each, N=35	Social Skills Training, N=35	76	Global Cognition<br>Processing Speed<br>Working Memory<br>Verbal Memory<br>Visual Memory<br>Executive Functions<br>Global Functioning<br>Global Symptoms
Pijnenborg 2019	The Nederlands, multi-center<br><br>In- and outpatients	Gender: 21,5% female<br>Age: 39,7 years<br>Illness duration: 12,7 years<br>Onset: 27 years<br>Education: n.i.<br>IQ: n.i.<br>Diagnosis: schizophrenia<br>Baseline PANSS: 62,6<br>Daily drug dose (CPZeq): n.i.	N=121, 13% attrition rate	6	Cognitive Remediation Therapy (CRT), 2 sessions/week of 1h each, N=62	REFLEX program, N=59	72	Global Cognition<br>Social Cognition<br>Global Functioning<br>Global Symptoms<br>Positive Symptoms<br>Negative Symptoms
Pontes 2013	Brazil, single center<br><br>Outpatients	Gender: 18% female<br>Age: 38,1 years<br>Illness duration: 15,2 years	N=17, 6% attrition rate	20	Cognitive Attention and Memory Training, 1 session/week of 40-60min, N=9	Newspaper reading groups, N=8	72	Global Cognition<br>Processing Speed<br>Working Memory<br>Verbal Memory<br>Visual Memory

© 2021 Vita et al. *JAMA Psychiatry.*

		Onset: 23 years<br>Education: 10,3 years<br>IQ: 93,7<br>Diagnosis: schizophrenia<br>Baseline PANSS: 48<br>Daily drug dose (CPZeq): n.i.						Executive Functions<br>Global Symptoms<br>Positive Symptoms<br>Negative Symptoms
Popova 2014	Germany, single center<br><br>Inpatients	Gender: 33,8% female<br>Age: 37,3 years<br>Illness duration and onset: n.i.<br>Education: 11,2 years<br>IQ: 106,3<br>Diagnosis: schizophrenia<br>Baseline PANSS: 70,3<br>Daily drug dose (CPZeq): 601,4 mg/day	N=80, 24% attrition rate	4	Computer-assisted Facial affect and working memory training, N=29 or Auditory discrimination and working memory training, N=27<br>In both cases 5 sessions/week of 1h each	Treatment as usual, N=24	58	Global Cognition<br>All cognitive domains<br>Global Functioning<br>Global Symptoms<br>Positive Symptoms<br>Negative Symptoms
Puig 2014	Spain, single center<br><br>Outpatients<br><br>Adolescents	Gender: 48% female<br>Age: 16,7 years<br>Illness duration: 1,4 years<br>Onset: 15,3 years<br>Education: n.i.<br>IQ: n.i.<br>Diagnosis: schizophrenia (88%), schizoaffective disorder<br>Baseline PANSS: 60,1<br>Daily drug dose (CPZeq): 451,8 mg/day	N=51, 43% attrition rate	20	Cognitive Remediation Therapy (CRT), 2 sessions/week, N=25	Treatment as usual (including psychoeducation), N=26	62	Global Cognition<br>Processing Speed<br>Working Memory<br>Verbal Memory<br>Visual Memory<br>Global Functioning<br>Global Symptoms
Rakitzi 2016	Greece, single center<br><br>Outpatients	Gender: 33% female<br>Age: 32,6 years<br>Illness duration: 5,7 years<br>Onset: 26,9 years<br>Education: n.i.<br>IQ: 89,8<br>Diagnosis: schizophrenia<br>Baseline PANSS: 118,4<br>Daily drug dose (CPZeq): 527,1 mg/day	N=48, 25% attrition rate	10	Integrate Psychological Therapy (IPT), cognitive subprograms, 2 sessions/week of 1h each, N=24	Treatment as usual, N=24	65	Global Cognition<br>Attention/Vigilance<br>Working Memory<br>Verbal Memory<br>Global Functioning<br>Global Symptoms<br>Positive Symptoms<br>Negative Symptoms
Ramsay 2017	USA, single center<br><br>Outpatients	Gender: n.i.<br>Age: 44,2 years<br>Illness duration: 19,9 years<br>Onset: 24,3 years	N=27, 4% attrition rate	16	Computer-assisted remediation, 3 sessions/week of 1h each, N=15	Computer skills training, N=12	57	Global Cognition<br>Working Memory<br>Global Functioning

© 2021 Vita et al. *JAMA Psychiatry.*

		Education: 13 years<br>IQ: 102,8<br>Diagnosis: schizophrenia, schizoaffective disorder<br>Baseline PANSS: 59,3<br>Daily drug dose (CPZeq): 449,1 mg/day						
Rass 2012	USA, single center<br><br>Outpatients	Gender: 33,3% female<br>Age: 41,5 years<br>Illness duration: 18,1 years<br>Onset: 23,4 years<br>Education: n.i.<br>IQ: 95,1<br>Diagnosis: schizophrenia (50%), schizoaffective disorder<br>Baseline PANSS: 56,9<br>Daily drug dose (CPZeq): n.i.	N=48, 6% attrition rate	10	Computer-assisted remediation using Posit Science tasks, 2 sessions/week of 2h each, N=21	TV watching, N=17, or Treatment as usual, N=10	65	Global Cognition<br>Processing Speed<br>Working Memory<br>Verbal Memory<br>Visual Memory
Reeder 2017	Great Britain, single center<br><br>In- and outpatients	Gender: 35,5% female<br>Age: 38,3 years<br>Illness duration and onset: n.i.<br>Education: 13,2 years<br>IQ: 93,5<br>Diagnosis: schizophrenia, schizoaffective disorder<br>Baseline PANSS: n.i.<br>Daily drug dose (CPZeq): 333,3 mg/day	N=93, 6,5% attrition rate	12	Computer-assisted remediation using CIRCuiTS, 3 sessions/week of 1h each, N=46	Multidisciplinary treatment as usual, N=47	80	Global Cognition<br>Working Memory<br>Visual Memory<br>Global Functioning<br>Positive Symptoms<br>Negative Symptoms
Roberts 2014	USA, single center<br><br>Outpatients	Gender: 33% female<br>Age: 39,7 years<br>Illness duration: 16,8 years<br>Onset: 23 years<br>Education: n.i.<br>IQ: 99,2<br>Diagnosis: schizophrenia spectrum disorder (42% schizophrenia)<br>Baseline PANSS: 65,3<br>Daily drug dose (CPZeq): 632,6 mg/day	N=66, 9% attrition rate	26	Social Cognition and Interaction Training (SCIT), 1h/week, N=33	Treatment as usual, N=33	64	Global Cognition<br>Social Cognition<br>Global Functioning<br>Global Symptoms<br>Positive Symptoms<br>Negative Symptoms
Roncone 2004	Italy, single center	Gender: 35% female	N=20, no	22	Instrumental Enrichment program,	Treatment as usual, N=10	51	Global Cognition<br>Processing Speed

© 2021 Vita et al. *JAMA Psychiatry.*

	Inpatients	Age: 33,7 years<br>Illness duration: 14 years<br>Onset: 19,7 years<br>Education: 11,5 years<br>IQ: 84,2<br>Diagnosis: schizophrenia<br>Baseline PANSS: n.i.<br>Daily drug dose (CPZeq): n.i.	dropouts		modified version, 1h/week, N=10			Executive Functions<br>Social Cognition<br>Negative Symptoms
Royer 2012	France, single center<br><br>Outpatients	Gender: n.i.<br>Age: 32,8 years<br>Illness duration: 11,1 years<br>Onset: 21,7 years<br>Education: 11,4 years<br>IQ: 99,5<br>Diagnosis: schizophrenia<br>Baseline PANSS: n.i.<br>Daily drug dose (CPZeq): n.i.	N=58, 21% attrition rate	26	Computer-assisted remediation using Rehacom + paper-and-pencil exercises, 6h/week, N=31	Treatment as usual, N=27	47	Global Cognition<br>Attention/Vigilance<br>Processing Speed<br>Working Memory<br>Verbal Memory<br>Executive Functions
Sachs 2012	Austria, single center<br><br>In- and outpatients<br><br>Recent onset (<5 years)	Gender: 47% female<br>Age: 29,3 years<br>Illness duration: 5 years<br>Onset: 24,3 years<br>Education: 14,2 years<br>IQ: n.i.<br>Diagnosis: schizophrenia<br>Baseline PANSS: n.i.<br>Daily drug dose (CPZeq): n.i.	N=40, 5% attrition rate	6	Training of Affect Recognition (TAR), 2 sessions/week of 45min each, N=20	Treatment as usual, N=20	39	Global Cognition<br>Attention/Vigilance<br>Processing Speed<br>Working Memory<br>Executive Functions<br>Social Cognition<br>Global Functioning<br>Negative Symptoms
Sanchez 2014	Spain, single center<br><br>Inpatients	Gender: 24% female<br>Age: 35,5 years<br>Illness duration: 13,4 years<br>Onset: 22,1 years<br>Education: 9,8 years<br>IQ: n.i.<br>Diagnosis: schizophrenia<br>Baseline PANSS: 90,9<br>Daily drug dose (CPZeq): 718,3 mg/day	N=92, 9% attrition rate	12	REHACOP, 3 sessions/week of 90 min each, N=38	Group activities, N=54	67	Global Cognition<br>Processing Speed<br>Working Memory<br>Verbal Memory<br>Executive Functions<br>Global Functioning<br>Global Symptoms<br>Positive Symptoms<br>Negative Symptoms
Sartory 2005	Germany, single center	Gender: 33,3% female	N=42, no	3	Computer-assisted remediation using	Treatment as usual, Waiting list N=21	60	Global Cognition<br>Processing Speed

© 2021 Vita et al. *JAMA Psychiatry.*

	Outpatients	Age: 31,9 years<br>Illness duration: 6,2 years<br>Onset: 25,8 years<br>Education: 10,3 years<br>IQ: n.i.<br>Diagnosis: schizophrenia<br>Baseline PANSS: n.i.<br>Daily drug dose (CPZeq): 567,9 mg/day	dropouts		Cogpack, 5 sessions/week of 45min each, N=21			Working Memory<br>Verbal Memory
Sevos 2018	France, single center<br><br>In- and outpatients	Gender: 26% female<br>Age: 41,2 years<br>Illness duration: 15,1 years<br>Onset: 26,1 years<br>Education: 10,8 years<br>IQ: 96,7<br>Diagnosis: schizophrenia<br>Baseline PANSS: n.i.<br>Daily drug dose (CPZeq): n.i.	N=31, no dropouts	10	CineMotion, 1 sessions/week of 90 min, N=16	Treatment as usual including psychotherapy and psychoeducation, N=15	44	Global Cognition<br>Social Cognition
Silverstein 2005	USA, single center<br><br>Inpatients<br><br>Study defined as treatment resistant	Gender: 12,9% female<br>Age: 39,3 years<br>Illness duration and onset: n.i.<br>Education: 10,5 years<br>IQ: n.i.<br>Diagnosis: schizophrenia<br>Baseline PANSS: n.i.<br>Daily drug dose (CPZeq): n.i.	N=31, dropouts n.i.	6	Attentional Processing Training and Shaping + UCLA Skills Training, unspecified schedule, N=18	Manipulated treatment as usual + UCLA Skills Training, N=13	54	Global Cognition<br>Attention/Vigilance<br>Working Memory<br>Verbal Memory<br>Global Functioning<br>Positive Symptoms<br>Negative Symptoms
Silverstein 2009	USA, multi-center<br><br>In- and outpatients<br><br>Non responsive to vocational rehabilitation	Gender: 39% female<br>Age: 39 years<br>Illness duration and onset: n.i.<br>Education: 11,4 years<br>IQ: n.i.<br>Diagnosis: schizophrenia<br>Baseline PANSS: n.i.<br>Daily drug dose (CPZeq): 1035,3 mg/day	N=82, 22% attrition rate	34	Attentional Processing Shaping, 2 sessions/week of 1h each, + UCLA Basic Conversation Skills Training, N=47	UCLA Basic Conversation Skills Training, N=35	56	Global Functioning
So 2015	Hong Kong, single center<br><br>Outpatients	Gender: 45,5% female<br>Age: 33,9 years	N=44, 36% attrition rate	4	Metacognitive Training for delusions (MCTd), 1h/week, N=23	Treatment as usual, Waiting list N=21	65	Positive Symptoms

© 2021 Vita et al. *JAMA Psychiatry.*

	Chronic, with active positive symptoms	Illness duration and onset: n.i.<br>Education: 12,2 years<br>IQ: n.i.<br>Diagnosis: any psychotic disorder (61% schizophrenia)<br>Baseline PANSS: 75,1<br>Daily drug dose (CPZeq): 274,4 mg/day						
Spaulding 1999	USA, single center<br><br>Inpatients<br><br>Severe functional impairment	Gender: 38,5% female<br>Age: 35,7 years<br>Illness duration: 11,9 years<br>Onset: 23,8 years<br>Education: 11,9 years<br>IQ: n.i.<br>Diagnosis: any psychotic disorder (affective psychosis included, 75% schizophrenia)<br>Baseline PANSS: n.i.<br>Daily drug dose (CPZeq): 1609,7 mg/day	N=91, 1% attrition rate	26	Integrated Psychological Therapy (IPT), cognitive subprograms, 2-3 sessions/week of 1h each, N=49	Manualized supportive therapy; treatment as usual including Social skills training, N=42	79	Global Cognition<br>Attention/Vigilance<br>Processing Speed<br>Working Memory<br>Verbal Memory<br>Visual Memory<br>Executive Functions<br>Global Functioning<br>Global Symptoms<br>Positive Symptoms<br>Negative Symptoms
Tan 2013	China, single center<br><br>Outpatients	Gender: 43% female<br>Age: 34,7 years<br>Illness duration: 10,6 years<br>Onset: 24,1 years<br>Education: 11,1 years<br>IQ: n.i.<br>Diagnosis: schizophrenia (96%), schizoaffective disorder<br>Baseline PANSS: n.i.<br>Daily drug dose (CPZeq): n.i.	N=70, 34% attrition rate	12	Computer-assisted remediation, 3 sessions/week (5h/week), N=36	Physical exercise, N=34	79	Global Cognition<br>Processing Speed<br>Working Memory<br>Verbal Memory
Tan 2016	China, single center<br><br>Inpatients	Gender: 35% female<br>Age: 46,4 years<br>Illness duration: 22,7 years<br>Onset: 23,7 years<br>Education: 9,9 years<br>IQ: 79,9<br>Diagnosis: schizophrenia	N=104, 13,5% attrition rate	10	Cognitive Remediation Treatment (CRT), 40 sessions total, 1h each, N=52	Music and Dance therapy, N=52	67	Global Cognition<br>Processing Speed<br>Working Memory<br>Verbal Memory<br>Visual Memory<br>Executive Functions<br>Global Functioning<br>Global Symptoms<br>Positive Symptoms

© 2021 Vita et al. *JAMA Psychiatry.*

		Baseline PANSS: 66,4<br>Daily drug dose (CPZeq): 277,7 mg/day						Negative Symptoms
Tan 2019	China, multi-center<br><br>Inpatients	Gender: 38,6% female<br>Age: 45,1 years<br>Illness duration: 20,4 years<br>Onset: 24,5 years<br>Education: 11,8 years<br>IQ: n.i.<br>Diagnosis: schizophrenia<br>Baseline PANSS: 60,5<br>Daily drug dose (CPZeq): 391,3 mg/day	N=311, 14% attrition rate	12	Computer-assisted remediation, 4-5 sessions/week of 45min each, N=196	Leisure activities, N=115	83	Global Cognition<br>All cognitive domains<br>Global Functioning<br>Global Symptoms<br>Positive Symptoms<br>Negative Symptoms
Tao 2015	China, single center<br><br>Inpatients	Gender: 45% female<br>Age: 29,3 years<br>Illness duration and onset: n.i.<br>Education: n.i.<br>IQ: n.i.<br>Diagnosis: schizophrenia<br>Baseline PANSS: 93,2<br>Daily drug dose (CPZeq): n.i.	N=86, no dropouts	12	Own program, 2 sessions/week of 30min each, N=44	Treatment as usual (maintenance medication only), N=42	26	Global Cognition<br>Processing Speed<br>Working Memory<br>Executive Functions<br>Global Symptoms
Tas 2012	Turkey, single center<br><br>Outpatients	Gender: 49% female<br>Age: 35,2 years<br>Illness duration: 12,2 years<br>Onset: 23 years<br>Education: 11,1 years<br>IQ: n.i.<br>Diagnosis: schizophrenia<br>Baseline PANSS: 58,9<br>Daily drug dose (CPZeq): 489,6 mg/day	N=49, 8% attrition rate	14	Social Cognition and Interaction Training, Family version (F-SCIT), 1 session/week of 80min, N=22	Social Stimulation, N=27	52	Global Cognition<br>Social Cognition<br>Global Functioning<br>Global Symptoms<br>Positive Symptoms<br>Negative Symptoms
Thomas 2018	USA, single center<br><br>Inpatients in forensic setting<br><br>Post-acute phase	Gender: 52% female<br>Age: 35,1 years<br>Illness duration: 15,7 years<br>Onset: 19,4 years<br>Education: 11,8 years<br>IQ: n.i.<br>Diagnosis: schizophrenia, schizoaffective disorder	N=46, 24% attrition rate	13	Targeted computer-assisted remediation using BrainHQ, 3-5 sessions/week of 1h each, N=24	Treatment as usual including psychotherapy, N=22	48	Global Cognition<br>Attention/Vigilance<br>Processing Speed<br>Working Memory<br>Verbal Memory<br>Visual Memory<br>Executive Functions<br>Positive Symptoms<br>Negative Symptoms

© 2021 Vita et al. *JAMA Psychiatry.*

		Baseline PANSS: n.i.<br>Daily drug dose (CPZeq): 1163,5 mg/day						
Twamley 2008-2012	USA, multi-center<br><br>Outpatients	Gender: 35% female<br>Age: 46,3 years<br>Illness duration: 23,3 years<br>Onset: 23,1 years<br>Education: 12,9 years<br>IQ: 106,9<br>Diagnosis: any psychotic disorder (affective psychosis included, 54% schizophrenia)<br>Baseline PANSS: n.i.<br>Daily drug dose (CPZeq): 383,8 mg/day	N=69, 26% attrition rate	12	Compensatory Cognitive Training (CCT), 2h sessions, unclear frequency, N=38	Treatment as usual (maintenance medication only), N=31	55	Global Cognition<br>Processing Speed<br>Working Memory<br>Verbal Memory<br>Global Functioning<br>Negative Symptoms
Ueland 2004	Norway, single center<br><br>Inpatients<br><br>Adolescents	Gender: 46,2% female<br>Age: 15,3 years<br>Illness duration, onset and education: n.i.<br>IQ: 88,6<br>Diagnosis: any psychotic disorder (affective included, 62% schizophrenia)<br>Baseline PANSS: 73,2<br>Daily drug dose (CPZeq): n.i.	N=26, dropouts n.i.	12	Own program, with 2 out of 4 subprograms derived from IPT, 30h total, + Psychoeducation, N=14	Psychoeducation, N=12	50	Global Cognition<br>Attention/Vigilance<br>Processing Speed<br>Working Memory<br>Visual Memory<br>Executive Functions<br>Global Functioning<br>Global Symptoms<br>Positive Symptoms<br>Negative Symptoms
Van Oosterhout 2014	The Nederlands, multi-center<br><br>In- and outpatients<br><br>Chronic, with active positive symptoms	Gender: 28,6% female<br>Age: 37,5 years<br>Illness duration, onset and education: n.i.<br>IQ: n.i.<br>Diagnosis: any psychotic disorder (64% schizophrenia)<br>Baseline PANSS: n.i.<br>Daily drug dose (CPZeq): n.i.	N=154, 28% attrition rate	8	Metacognitive Training (MCT), 1h/week, N=75	Treatment as usual, N=79	84	Global Cognition<br>Social Cognition<br>Positive Symptoms
Vaskinn 2019	Norway, single center<br><br>Outpatients	Gender: 33,3% female<br>Age: 30,4 years<br>Illness duration: 7,9 years	N=48, 17% attrition rate	8	Training of Affect Recognition (TAR), 2 sessions/week, N=24	Treatment as usual including psychoeducation and CBT, N=24	62	Global Cognition<br>Social Cognition<br>Global Functioning<br>Global Symptoms<br>Positive Symptoms

© 2021 Vita et al. *JAMA Psychiatry.*

		Onset: 22,5 years<br>Education: 12,2 years<br>IQ: 101,9<br>Diagnosis: schizophrenia (81%), schizoaffective disorder<br>Baseline PANSS: 43,7<br>Daily drug dose (CPZeq): n.i.						Negative Symptoms
Vauth 2005	Germany, single center<br><br>Inpatients	Gender: 31,9% female<br>Age: 28,8 years<br>Illness duration: 6,1 years<br>Onset: 22,7 years<br>Education: 12,6 years<br>IQ: n.i.<br>Diagnosis: schizophrenia<br>Baseline PANSS: n.i.<br>Daily drug dose (CPZeq): n.i.	N=138, 27,5% attrition rate	8	Integrative remediation (CAT + Cogpack + non-computerized training), 2 sessions/week of 90 min each, + Vocational rehabilitation, N=47	Self-management training for negative symptoms + Vocational rehabilitation, N=45, or Vocational rehabilitation only, N=46	66	Global Cognition<br>Processing Speed<br>Verbal Memory<br>Executive Functions<br>Negative Symptoms
Ventura 2019	USA, single center<br><br>Outpatients<br><br>Recent onset (<2 years)	Gender: 22% female<br>Age: 21,5 years<br>Illness duration: 0,7 years<br>Onset: 20,8 years<br>Education: 12,4 years<br>IQ: n.i.<br>Diagnosis: non-affective psychosis (56% schizophrenia)<br>Baseline PANSS: n.i.<br>Daily drug dose (CPZeq): n.i.	N=80, attrition rate n.i.	52	Neurocognitive Enhancement Therapy (NET) + Neuropsychological and Educational Approach to Remediation (NEAR), flexible schedule over time (from 3h/week to 2h/week every 2 weeks), N=39	Healthy Behavior training, N=41	54	Global Functioning<br>Negative Symptoms
Vidarsdottir 2019	Iceland, single center<br><br>Outpatients<br><br>Recent onset	Gender: 12,3% female<br>Age: 24,2 years<br>Illness duration: 1,9 years<br>Onset: 22,3 years<br>Education: 11,3 years<br>IQ: n.i.<br>Diagnosis: any psychotic disorder (69% schizophrenia)<br>Baseline PANSS: n.i.	N=49, 6% attrition rate	12	Integrative program including elements from SCIT and NEAR and computerized tasks, 2 sessions/week of 2h each, N=25	Treatment as usual, N=24	74	Global Cognition<br>Processing Speed<br>Working Memory<br>Verbal Memory<br>Executive Functions<br>Social Cognition<br>Global Functioning<br>Positive Symptoms<br>Negative Symptoms

© 2021 Vita et al. *JAMA Psychiatry.*

		Daily drug dose (CPZeq): n.i.						
Vita 2011 a	Italy, multi-center<br><br>Inpatients	Gender: 30,9% female<br>Age: 39 years<br>Illness duration: 15,9 years<br>Onset: 23,1 years<br>Education: 10,5 years<br>IQ: 86,7<br>Diagnosis: schizophrenia<br>Baseline PANSS: 88,1<br>Daily drug dose (CPZeq): 660,1 mg/day	N=84, 2% attrition rate	24	Integrated Psychological Therapy (IPT), cognitive subprograms, N=26<br>Computer-assisted remediation using Cogpack, N=30<br>In both cases: 2 sessions/week of 45min each	Non-cognitive oriented rehabilitation adjunctive on treatment as usual, N=28	72	Global Cognition<br>Processing Speed<br>Working Memory<br>Verbal Memory<br>Executive Functions<br>Global Functioning<br>Global Symptoms<br>Positive Symptoms<br>Negative Symptoms
Vita 2011 b	Italy, single center<br><br>In- and outpatients	Gender: 16% female<br>Age: 37,2 years<br>Illness duration: 13,7 years<br>Onset: 23,5 years<br>Education: 10,4 years<br>IQ: n.i.<br>Diagnosis: schizophrenia<br>Baseline PANSS: 94,9<br>Daily drug dose (CPZeq): 847,6 mg/day	N=32, 3% attrition rate	24	Integrated Psychological Therapy (IPT), cognitive subprograms, 2 sessions/week of 45 min each, N=16	Psychosocial rehabilitation adjunctive on treatment as usual, N=16	68	Global Cognition<br>Processing Speed<br>Working Memory<br>Verbal Memory<br>Executive Functions<br>Global Functioning<br>Global Symptoms<br>Positive Symptoms<br>Negative Symptoms
Wölwer 2005	Germany, single center<br><br>In- and outpatients	Gender: 22,1% female<br>Age: 34,3 years<br>Illness duration, onset and education: n.i.<br>IQ: 106,4<br>Diagnosis: schizophrenia<br>Baseline PANSS: 64,2<br>Daily drug dose (CPZeq): n.i.	N=77, 31% attrition rate	6	Training of Affect Recognition (TAR), N=28, or Computer-assisted remediation using Cogpack, N=24<br>In both cases: 2 sessions/week of 45min each	Treatment as usual, N=25	64	Global Cognition<br>Working Memory<br>Verbal Memory<br>Social Cognition<br>Global Symptoms<br>Positive Symptoms<br>Negative Symptoms
Wykes 1999	Great Britain, multi-center<br><br>Outpatients	Gender: 24,2% female<br>Age: 38,4 years<br>Illness duration and onset: n.i.<br>Education: 12,3 years<br>IQ: n.i.<br>Diagnosis: schizophrenia<br>Baseline PANSS: 59,3<br>Daily drug dose (CPZeq): n.i.	N=33, 12% attrition rate	12	Cognitive Remediation Therapy (CRT), at least 3 sessions/week of 1h each, N=17	Occupational therapy, N=16	68	Global Cognition<br>Processing Speed<br>Working Memory<br>Executive Functions<br>Global Functioning<br>Global Symptoms
Wykes 2007 a	Great Britain, single center	Gender: 35% female	N=40, 22,5%	14	Cognitive Remediation Therapy (CRT), 3	Treatment as usual, N=19	60	Global Cognition<br>Working Memory

© 2021 Vita et al. *JAMA Psychiatry.*

	Inpatients<br><br>Adolescents and recent onset (<5 years)	Age: 18,2 years<br>Illness duration: 1,2 years<br>Onset: 17 years<br>Education: n.i.<br>IQ: n.i.<br>Diagnosis: schizophrenia<br>Baseline PANSS: 67,9<br>Daily drug dose (CPZeq): n.i.	attrition rate		sessions/week of 1h each, N=21			Executive Functions<br>Global Functioning<br>Global Symptoms
Wykes 2007 b	Great Britain, multi-center<br><br>Outpatients<br><br>Severe functional impairment	Gender: 27% female<br>Age: 36 years<br>Illness duration, onset and education: n.i.<br>IQ: n.i.<br>Diagnosis: schizophrenia<br>Baseline PANSS: 59,9<br>Daily drug dose (CPZeq): 334,4 mg/day	N=85, 8% attrition rate	12	Cognitive Remediation Therapy (CRT), 3 sessions/week, N=43	Treatment as usual, N=42	87	Global Cognition<br>Working Memory<br>Executive Functions<br>Global Functioning<br>Global Symptoms<br>Positive Symptoms<br>Negative Symptoms
Zimmer 2007	Brazil, single center<br><br>Outpatients	Gender: 28% female<br>Age: 38,2 years<br>Illness duration: 16,5 years<br>Onset: 21,7 years<br>Education: n.i.<br>IQ: n.i.<br>Diagnosis: schizophrenia (95%), schizoaffective disorder<br>Baseline PANSS: n.i.<br>Daily drug dose (CPZeq): n.i.	N=66, 21% attrition rate	12	Integrated Psychological Therapy (IPT), 12week version, 1 session/week of 40min, N=23	Treatment as usual, N=43	66	Global Cognition<br>Global Functioning

CTAM: Clinical Trials Assessment Measure
IQ: Intelligence quotient
PANSS: Positive And Negative Syndrome Scale
CPZeq: Chlorpromazine equivalents

© 2021 Vita et al. *JAMA Psychiatry.*

eFigure 1. Forest plot for the effects of cognitive remediation (CR) on global cognition

Study or Subgroup	Std. Mean Difference	SE	CR Total	Control Total	Weight	Std. Mean Difference IV, Random, 95% CI
Ahmed 2015	0.444	0.23	42	36	1.0%	0.44 [-0.01, 0.89]
Ahuir 2018	0.368	0.381	14	14	0.4%	0.37 [-0.38, 1.11]
Aloi 2018	0.483	0.317	21	20	0.6%	0.48 [-0.14, 1.10]
Au 2015	-0.003	0.211	45	45	1.1%	-0.00 [-0.42, 0.41]
Bell 2001	0.516	0.253	31	34	0.8%	0.52 [0.02, 1.01]
Bellucci 2003	0.033	0.343	17	17	0.5%	0.03 [-0.64, 0.71]
Bowie 2012	0.72	0.245	36	35	0.9%	0.72 [0.24, 1.20]
Bowie 2012-2	0.68	0.244	36	35	0.9%	0.68 [0.20, 1.16]
Bryce 2018	0.305	0.269	29	27	0.8%	0.30 [-0.22, 0.83]
Burda 1994	0.56	0.249	40	29	0.9%	0.56 [0.07, 1.05]
Byrne 2013	0.52	0.367	14	17	0.5%	0.52 [-0.20, 1.24]
Cassetta 2019	0.507	0.274	28	27	0.8%	0.51 [-0.03, 1.04]
Cassetta 2019-2	0.574	0.276	28	27	0.7%	0.57 [0.03, 1.11]
Cavallaro 2009	0.215	0.219	50	36	1.0%	0.21 [-0.21, 0.64]
Choi 2006	0.621	0.487	10	8	0.3%	0.62 [-0.33, 1.58]
Choi 2017	0.315	0.331	18	19	0.6%	0.32 [-0.33, 0.96]
D'Amato 2011	0.185	0.228	39	38	1.0%	0.18 [-0.26, 0.63]
D'Souza 2013	0.076	0.298	22	23	0.7%	0.08 [-0.51, 0.66]
Dickinson 2010	0.38	0.26	34	27	0.8%	0.38 [-0.13, 0.89]
Donohoe 2018	0.128	0.271	25	30	0.8%	0.13 [-0.40, 0.66]
Drake 2014	-0.082	0.258	30	30	0.8%	-0.08 [-0.59, 0.42]
Eack 2009	0.187	0.264	31	27	0.8%	0.19 [-0.33, 0.70]
Fan 2017	0.005	0.417	12	11	0.4%	0.01 [-0.81, 0.82]
Farreny 2012	0.292	0.277	29	24	0.7%	0.29 [-0.25, 0.83]
Fernandez-Gonzalo 2015	0.2	0.317	21	19	0.6%	0.20 [-0.42, 0.82]
Fisher 2009-2016	0.357	0.217	46	41	1.1%	0.36 [-0.07, 0.78]
Fisher 2015	0.429	0.184	63	58	1.3%	0.43 [0.07, 0.79]
Fiszdon 2016	0.252	0.246	50	25	0.9%	0.25 [-0.23, 0.73]
Garcia 2003	-0.124	0.45	11	9	0.3%	-0.12 [-1.01, 0.76]
Garcia-Fernandez 2019	-0.032	0.219	36	50	1.0%	-0.03 [-0.46, 0.40]
Garrido 2013	0.594	0.303	31	18	0.6%	0.59 [0.00, 1.19]
Gaweda 2015	0.103	0.302	23	21	0.6%	0.10 [-0.49, 0.69]
Gharaeipour 2012	0.694	0.318	21	21	0.6%	0.69 [0.07, 1.32]
Gohar 2013	0.076	0.309	22	20	0.6%	0.08 [-0.53, 0.68]
Gomar 2015	0.155	0.231	39	36	1.0%	0.15 [-0.30, 0.61]
Gomar 2015-2	-0.027	0.243	32	36	0.9%	-0.03 [-0.50, 0.45]
Gordon 2018	0.225	0.351	18	15	0.5%	0.23 [-0.46, 0.91]
Greig 2007	0.186	0.237	38	34	0.9%	0.19 [-0.28, 0.65]
Habel 2010	0.742	0.463	10	10	0.3%	0.74 [-0.17, 1.65]
Hadas-Lidor 2001	0.678	0.27	29	29	0.8%	0.68 [0.15, 1.21]
Hegde 2012	0.914	0.383	13	18	0.4%	0.91 [0.16, 1.66]
Hermanutz 1991	0.149	0.448	10	10	0.3%	0.15 [-0.73, 1.03]
Hermanutz 1991-2	-0.007	0.447	10	10	0.3%	-0.01 [-0.88, 0.87]
Hodge 2010	0.226	0.319	22	18	0.6%	0.23 [-0.40, 0.85]
Hogarty 2004	0.255	0.196	61	46	1.2%	0.26 [-0.13, 0.64]
Hooker 2012	0.274	0.429	11	11	0.4%	0.27 [-0.57, 1.11]
Horan 2009	-0.183	0.36	15	16	0.5%	-0.18 [-0.89, 0.52]
Horan 2011	-0.015	0.324	19	19	0.6%	-0.01 [-0.65, 0.62]
Horan 2011-2	0.774	0.352	16	19	0.5%	0.77 [0.08, 1.46]
Horan 2011-3	0.181	0.353	14	19	0.5%	0.18 [-0.51, 0.87]
Horan 2018	-0.146	0.214	41	47	1.1%	-0.15 [-0.57, 0.27]
Horan 2018-2	0.141	0.207	47	47	1.1%	0.14 [-0.26, 0.55]
Iwata 2017	0.441	0.271	28	28	0.8%	0.44 [-0.09, 0.97]
Jahshan 2019	-0.046	0.295	29	19	0.7%	-0.05 [-0.62, 0.53]
Jahshan 2019-2	0.014	0.285	35	19	0.7%	0.01 [-0.54, 0.57]
Kanie 2019	-0.006	0.25	32	32	0.9%	-0.01 [-0.50, 0.48]
Kantrowitz 2016	0.129	0.183	56	64	1.3%	0.13 [-0.23, 0.49]
Katsumi 2019	2.009	0.373	22	22	0.5%	2.01 [1.28, 2.74]
Keefe 2012	0.172	0.293	25	22	0.7%	0.17 [-0.40, 0.75]
Kidd 2014	0.196	0.365	18	13	0.5%	0.20 [-0.52, 0.91]
Kukla 2018	0.531	0.304	23	22	0.6%	0.53 [-0.06, 1.13]
Kurtz 2007	-0.03	0.31	23	19	0.6%	-0.03 [-0.64, 0.58]
Kurtz 2015	0.032	0.25	32	32	0.9%	0.03 [-0.46, 0.52]
Lado-Colesido 2019	0.889	0.297	26	24	0.7%	0.89 [0.31, 1.47]
Lee 2013	0.681	0.266	30	30	0.8%	0.68 [0.16, 1.20]
Lindenmayer 2008	0.158	0.238	41	31	0.9%	0.16 [-0.31, 0.62]
Lu 2012	1.067	0.194	60	62	1.2%	1.07 [0.69, 1.45]
Mahncke 2019	-0.117	0.169	68	73	1.4%	-0.12 [-0.45, 0.21]
Mak 2013	-0.091	0.222	41	40	1.0%	-0.09 [-0.53, 0.34]
Man 2012	0.065	0.265	27	30	0.8%	0.07 [-0.45, 0.58]
Man 2012-2	0.105	0.277	23	30	0.7%	0.10 [-0.44, 0.65]
Marono Souto 2018	0.536	0.263	30	30	0.8%	0.54 [0.02, 1.05]
Matsuda 2018	0.267	0.255	31	31	0.8%	0.27 [-0.23, 0.77]
Matsui 2009	0.079	0.45	11	9	0.3%	0.08 [-0.80, 0.96]
McGurk 2005	0.585	0.308	23	21	0.6%	0.58 [-0.02, 1.19]
McGurk 2016	0.268	0.283	28	23	0.7%	0.27 [-0.29, 0.82]
Medalia 1998	0.223	0.273	27	27	0.8%	0.22 [-0.31, 0.76]
Medalia 2000	-0.006	0.333	18	18	0.5%	-0.01 [-0.66, 0.65]
Medalia 2000-2	-0.141	0.334	18	18	0.5%	-0.14 [-0.80, 0.51]
Meichenbaum 1973	1.637	0.753	5	5	0.1%	1.64 [0.16, 3.11]
Mendella 2015	0.641	0.402	16	11	0.4%	0.64 [-0.15, 1.43]
Morimoto 2018	0.368	0.363	16	15	0.5%	0.37 [-0.34, 1.08]
Moritz 2011	0.733	0.345	18	18	0.5%	0.73 [0.06, 1.41]
Mueller 2015	0.266	0.161	81	75	1.5%	0.27 [-0.05, 0.58]
Mueller 2017	0.369	0.259	28	33	0.8%	0.37 [-0.14, 0.88]
O'Reilly 2019	0.207	0.249	32	33	0.9%	0.21 [-0.28, 0.70]
Ochoa 2017	0.084	0.213	48	41	1.1%	0.08 [-0.33, 0.50]
Ojeda 2012	0.64	0.224	44	40	1.0%	0.64 [0.20, 1.08]
Omiya 2016	0.096	0.486	8	9	0.3%	0.10 [-0.86, 1.05]
Ostergaard 2014	0.284	0.186	60	57	1.3%	0.28 [-0.08, 0.65]
Pena 2016	0.347	0.201	52	49	1.2%	0.35 [-0.05, 0.74]
Penades 2006	1.026	0.337	20	20	0.5%	1.03 [0.37, 1.69]
Penades 2013	0.076	0.338	17	18	0.5%	0.08 [-0.59, 0.74]
Penades 2018	1.077	0.256	35	35	0.8%	1.08 [0.58, 1.58]
Pijnenborg 2019	-0.165	0.196	50	55	1.2%	-0.17 [-0.55, 0.22]
Pontes 2013	-0.059	0.486	9	8	0.3%	-0.06 [-1.01, 0.89]
Popova 2014	0.18	0.325	19	19	0.6%	0.18 [-0.46, 0.82]
Popova 2014-2	0.022	0.324	19	19	0.6%	0.02 [-0.61, 0.66]
Puig 2014	0.408	0.286	25	25	0.7%	0.41 [-0.15, 0.97]
Rakitzi 2016	0.376	0.337	19	17	0.5%	0.38 [-0.28, 1.04]
Ramsay 2017	0.147	0.394	14	12	0.4%	0.15 [-0.63, 0.92]
Rass 2012	-0.023	0.343	17	17	0.5%	-0.02 [-0.70, 0.65]
Rass 2012-2	-0.084	0.399	17	10	0.4%	-0.08 [-0.87, 0.70]
Reeder 2017	0.249	0.208	46	47	1.1%	0.25 [-0.16, 0.66]
Roberts 2014	0.124	0.258	30	30	0.8%	0.12 [-0.38, 0.63]
Roncone 2004	0.581	0.458	10	10	0.3%	0.58 [-0.32, 1.48]
Royer 2012	0.487	0.307	28	18	0.6%	0.49 [-0.11, 1.09]
Sachs 2012	-0.164	0.325	20	18	0.6%	-0.16 [-0.80, 0.47]
Sanchez 2014	0.643	0.226	36	48	1.0%	0.64 [0.20, 1.09]
Sartory 2005	0.328	0.311	21	21	0.6%	0.33 [-0.28, 0.94]
Sevos 2018	0.217	0.361	16	15	0.5%	0.22 [-0.49, 0.92]
Silverstein 2005	0.281	0.366	18	13	0.5%	0.28 [-0.44, 1.00]
Spaulding 1999	0.08	0.211	48	42	1.1%	0.08 [-0.33, 0.49]
Tan 2013	0.925	0.252	36	34	0.9%	0.93 [0.43, 1.42]
Tan 2016	0.33	0.197	52	52	1.2%	0.33 [-0.06, 0.72]
Tan 2019	0.192	0.118	196	115	2.0%	0.19 [-0.04, 0.42]
Tao 2015	0.832	0.225	44	42	1.0%	0.83 [0.39, 1.27]
Tas 2012	0.585	0.308	19	26	0.6%	0.58 [-0.02, 1.19]
Thomas 2018	0.197	0.296	24	22	0.7%	0.20 [-0.38, 0.78]
Twamley 2008-2012	0.295	0.243	38	31	0.9%	0.29 [-0.18, 0.77]
Ueland 2004	0.326	0.396	14	12	0.4%	0.33 [-0.45, 1.10]
Van Oosterhout 2014	-0.283	0.191	51	60	1.2%	-0.28 [-0.66, 0.09]
Vaskinn 2019	-0.175	0.317	20	20	0.6%	-0.17 [-0.80, 0.45]
Vauth 2005	0.411	0.238	37	35	0.9%	0.41 [-0.06, 0.88]
Vauth 2005-2	0.24	0.251	37	28	0.9%	0.24 [-0.25, 0.73]
Vidarsdottir 2019	0.317	0.288	25	24	0.7%	0.32 [-0.25, 0.88]
Vita 2011a	0.337	0.274	26	28	0.8%	0.34 [-0.20, 0.87]
Vita 2011a-2	0.288	0.264	30	28	0.8%	0.29 [-0.23, 0.81]
Vita 2011b	0.361	0.363	16	15	0.5%	0.36 [-0.35, 1.07]
Wolwer 2005	0.624	0.282	28	25	0.7%	0.62 [0.07, 1.18]
Wolwer 2005-2	0.372	0.288	24	25	0.7%	0.37 [-0.19, 0.94]
Wykes 1999	0.177	0.349	17	16	0.5%	0.18 [-0.51, 0.86]
Wykes 2007a	0.13	0.317	21	19	0.6%	0.13 [-0.49, 0.75]
Wykes 2007b	0.211	0.227	39	39	1.0%	0.21 [-0.23, 0.66]
Zimmer 2007	0.401	0.282	20	36	0.7%	0.40 [-0.15, 0.95]
**Total (95% CI)**			**4031**	**3782**	**100.0%**	**0.29 [0.24, 0.34]**

Heterogeneity: $Tau^2 = 0.02$; $Chi^2 = 175.71$, df = 134 (P = 0.009); $I^2 = 24\%$

Test for overall effect: Z = 10.78 (P < 0.00001)

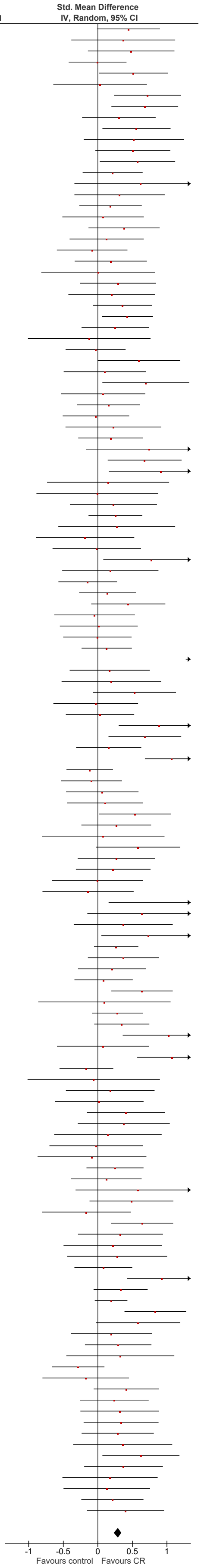

© 2021 Vita et al. *JAMA Psychiatry.*

eFigure 2. Forest plot for the effects of cognitive remediation (CR) on global functioning

Study or Subgroup	Std. Mean Difference	SE	CR Total	Control Total	Weight	Std. Mean Difference IV, Random, 95% CI
Ahmed 2015	0.286	0.228	42	36	1.3%	0.29 [-0.16, 0.73]
Ahuir 2018	0.006	0.378	14	14	0.6%	0.01 [-0.73, 0.75]
Aloi 2018	0.444	0.316	21	20	0.8%	0.44 [-0.18, 1.06]
Au 2015	-0.07	0.211	45	45	1.4%	-0.07 [-0.48, 0.34]
Bowie 2012	-0.15	0.238	36	35	1.2%	-0.15 [-0.62, 0.32]
Bowie 2012-2	0.12	0.238	36	35	1.2%	0.12 [-0.35, 0.59]
Briki 2014	0.049	0.283	25	25	1.0%	0.05 [-0.51, 0.60]
Bryce 2018	-0.072	0.268	29	27	1.0%	-0.07 [-0.60, 0.45]
Byrne 2013	0.71	0.373	14	17	0.6%	0.71 [-0.02, 1.44]
Cassetta 2019	0.415	0.273	28	27	1.0%	0.41 [-0.12, 0.95]
Cassetta 2019-2	0.49	0.274	28	27	1.0%	0.49 [-0.05, 1.03]
Cavallaro 2009	0.205	0.219	50	36	1.3%	0.20 [-0.22, 0.63]
D'Amato 2011	0.073	0.228	39	38	1.3%	0.07 [-0.37, 0.52]
D'Souza 2013	0.137	0.299	22	23	0.9%	0.14 [-0.45, 0.72]
Dickinson 2010	-0.183	0.258	34	27	1.1%	-0.18 [-0.69, 0.32]
Donohoe 2018	0.488	0.275	25	30	1.0%	0.49 [-0.05, 1.03]
Drake 2014	-0.323	0.26	30	30	1.1%	-0.32 [-0.83, 0.19]
Eack 2009	1.508	0.299	31	27	0.9%	1.51 [0.92, 2.09]
Farreny 2012	0.53	0.281	29	24	1.0%	0.53 [-0.02, 1.08]
Fernandez-Gonzalo 2015	0.074	0.317	21	19	0.8%	0.07 [-0.55, 0.70]
Fisher 2009-2016	-0.166	0.215	0	0	1.4%	-0.17 [-0.59, 0.26]
Fisher 2015	-0.066	0.182	63	58	1.6%	-0.07 [-0.42, 0.29]
Fiszdon 2016	0.026	0.245	50	25	1.2%	0.03 [-0.45, 0.51]
Galderisi 2010	0.754	0.297	26	23	0.9%	0.75 [0.17, 1.34]
Garcia 2003	0.251	0.451	11	9	0.5%	0.25 [-0.63, 1.13]
Garcia-Fernandez 2019	0.041	0.219	36	50	1.3%	0.04 [-0.39, 0.47]
Garrido 2013	1.077	0.316	31	18	0.8%	1.08 [0.46, 1.70]
Gomar 2015	-0.038	0.231	39	36	1.2%	-0.04 [-0.49, 0.41]
Gomar 2015-2	0.223	0.244	32	36	1.2%	0.22 [-0.26, 0.70]
Gordon 2018	0.244	0.351	18	15	0.7%	0.24 [-0.44, 0.93]
Hermanutz 1991	-0.088	0.447	10	10	0.5%	-0.09 [-0.96, 0.79]
Hermanutz 1991-2	-0.099	0.448	10	10	0.5%	-0.10 [-0.98, 0.78]
Hogarty 2004	0.3	0.196	61	46	1.5%	0.30 [-0.08, 0.68]
Hooker 2012	-0.295	0.429	11	11	0.5%	-0.29 [-1.14, 0.55]
Horan 2011	0.382	0.328	19	19	0.8%	0.38 [-0.26, 1.02]
Horan 2011-2	0.372	0.342	16	19	0.7%	0.37 [-0.30, 1.04]
Horan 2011-3	0.235	0.353	14	19	0.7%	0.23 [-0.46, 0.93]
Horan 2018	0.143	0.214	41	47	1.4%	0.14 [-0.28, 0.56]
Horan 2018-2	-0.026	0.206	47	47	1.4%	-0.03 [-0.43, 0.38]
Iwata 2017	0.548	0.272	28	28	1.0%	0.55 [0.01, 1.08]
Jahshan 2019	0.202	0.296	29	19	0.9%	0.20 [-0.38, 0.78]
Jahshan 2019-2	0.195	0.286	35	19	1.0%	0.20 [-0.37, 0.76]
Kanie 2019	0.11	0.25	32	32	1.1%	0.11 [-0.38, 0.60]
Kantrowitz 2016	0.2	0.183	56	64	1.6%	0.20 [-0.16, 0.56]
Katsumi 2019	0.336	0.304	22	22	0.9%	0.34 [-0.26, 0.93]
Keefe 2012	0.087	0.292	25	22	0.9%	0.09 [-0.49, 0.66]
Klingberg 2011	-0.204	0.143	99	99	2.0%	-0.20 [-0.48, 0.08]
Kukla 2018	0.24	0.299	23	22	0.9%	0.24 [-0.35, 0.83]
Kurtz 2015	0.134	0.25	32	32	1.1%	0.13 [-0.36, 0.62]
Lee 2013	0.736	0.236	30	30	1.2%	0.74 [0.27, 1.20]
Lu 2012	0.732	0.187	60	62	1.6%	0.73 [0.37, 1.10]
Mahncke 2019	0.02	0.169	68	73	1.7%	0.02 [-0.31, 0.35]
Matsuda 2018	0.095	0.254	31	31	1.1%	0.10 [-0.40, 0.59]
Medalia 2000	-0.577	0.341	18	18	0.7%	-0.58 [-1.25, 0.09]
Medalia 2000-2	1.486	0.379	18	18	0.6%	1.49 [0.74, 2.23]
Mendella 2015	0.043	0.392	16	11	0.6%	0.04 [-0.73, 0.81]
Morimoto 2018	0.15	0.36	16	15	0.7%	0.15 [-0.56, 0.86]
Mueller 2015	0.27	0.161	81	75	1.8%	0.27 [-0.05, 0.59]
Mueller 2017	-0.094	0.257	28	33	1.1%	-0.09 [-0.60, 0.41]
O'Reilly 2019	-0.042	0.248	32	33	1.1%	-0.04 [-0.53, 0.44]
Ochoa 2017	0.013	0.213	48	41	1.4%	0.01 [-0.40, 0.43]
Ojeda 2012	0.063	0.219	44	40	1.3%	0.06 [-0.37, 0.49]
Omiya 2016	0.813	0.508	8	9	0.4%	0.81 [-0.18, 1.81]
Ostergaard 2014	-0.007	0.185	60	57	1.6%	-0.01 [-0.37, 0.36]
Pena 2016	0.443	0.202	52	49	1.4%	0.44 [0.05, 0.84]
Penades 2006	0.737	0.327	20	20	0.8%	0.74 [0.10, 1.38]
Penades 2018	0.454	0.242	35	35	1.2%	0.45 [-0.02, 0.93]
Pijnenborg 2019	-0.19	0.196	50	55	1.5%	-0.19 [-0.57, 0.19]
Popova 2014	0.152	0.325	19	19	0.8%	0.15 [-0.48, 0.79]
Popova 2014-2	0.276	0.326	19	19	0.8%	0.28 [-0.36, 0.91]
Puig 2014	0.422	0.286	25	25	1.0%	0.42 [-0.14, 0.98]
Rakitzi 2016	0.196	0.335	19	17	0.8%	0.20 [-0.46, 0.85]
Ramsay 2017	-0.319	0.396	14	12	0.6%	-0.32 [-1.10, 0.46]
Reeder 2017	0.633	0.213	46	47	1.4%	0.63 [0.22, 1.05]
Roberts 2014	0.077	0.258	30	30	1.1%	0.08 [-0.43, 0.58]
Sachs 2012	0.306	0.327	20	18	0.8%	0.31 [-0.33, 0.95]
Sanchez 2014	0.526	0.224	36	48	1.3%	0.53 [0.09, 0.97]
Silverstein 2005	0.342	0.367	18	13	0.7%	0.34 [-0.38, 1.06]
Silverstein 2009	0.471	0.23	44	35	1.3%	0.47 [0.02, 0.92]
Spaulding 1999	0.454	0.214	48	42	1.4%	0.45 [0.03, 0.87]
Tan 2016	0.366	0.198	52	52	1.5%	0.37 [-0.02, 0.75]
Tan 2019	0.019	0.117	196	115	2.2%	0.02 [-0.21, 0.25]
Tas 2012	0.677	0.311	19	26	0.8%	0.68 [0.07, 1.29]
Twamley 2008-2012	0.121	0.242	38	31	1.2%	0.12 [-0.35, 0.60]
Ueland 2004	0.36	0.397	14	12	0.6%	0.36 [-0.42, 1.14]
Vaskinn 2019	-0.054	0.316	20	20	0.8%	-0.05 [-0.67, 0.57]
Ventura 2019	0.441	0.226	39	41	1.3%	0.44 [-0.00, 0.88]
Vidarsdottir 2019	-0.029	0.286	25	24	1.0%	-0.03 [-0.59, 0.53]
Vita 2011a	0.418	0.275	26	28	1.0%	0.42 [-0.12, 0.96]
Vita 2011a-2	0.818	0.274	30	28	1.0%	0.82 [0.28, 1.36]
Vita 2011b	0.511	0.366	16	15	0.7%	0.51 [-0.21, 1.23]
Wykes 1999	0.284	0.35	17	16	0.7%	0.28 [-0.40, 0.97]
Wykes 2007a	0.011	0.317	21	19	0.8%	0.01 [-0.61, 0.63]
Wykes 2007b	-0.08	0.227	39	39	1.3%	-0.08 [-0.52, 0.36]
Zimmer 2007	0.766	0.288	20	36	0.9%	0.77 [0.20, 1.33]
**Total (95% CI)**			**3140**	**2951**	**100.0%**	**0.22 [0.16, 0.29]**

Heterogeneity: Tau² = 0.04; Chi² = 150.29, df = 94 (P = 0.0002); I² = 37%

Test for overall effect: Z = 6.63 (P < 0.00001)

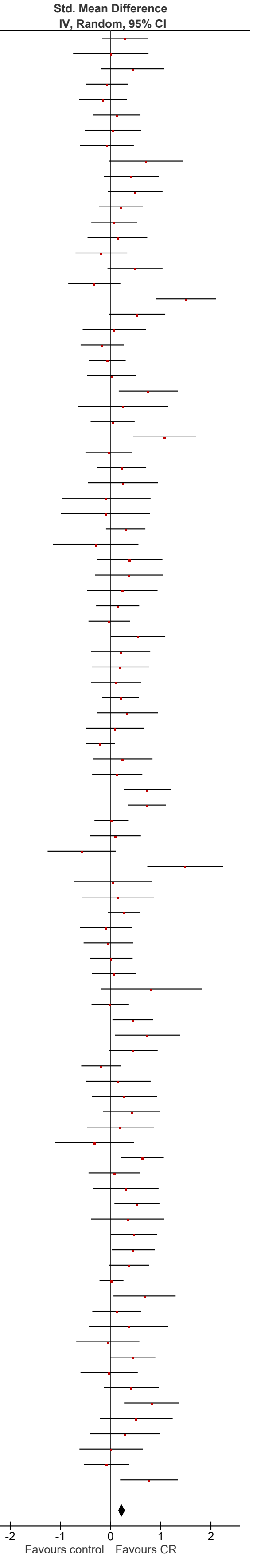

© 2021 Vita et al. *JAMA Psychiatry.*

eFigure 3. Subgroup analysis for the effects of interventions including all core elements of cognitive remediation (global cognition)

Study or Subgroup	Std. Mean Difference	SE	CR Total	Control Total	Weight	Std. Mean Difference IV, Random, 95% CI
**2.37.1 Not enough core elements**						
Ahmed 2015	0.444	0.23	42	36	1.0%	0.44 [-0.01, 0.89]
Ahuir 2018	0.368	0.381	14	14	0.4%	0.37 [-0.38, 1.11]
Au 2015	-0.003	0.211	45	45	1.1%	-0.00 [-0.42, 0.41]
Bell 2001	0.516	0.253	31	34	0.8%	0.52 [0.02, 1.01]
Bellucci 2003	0.033	0.343	17	17	0.5%	0.03 [-0.64, 0.71]
Bowie 2012	0.72	0.245	36	35	0.9%	0.72 [0.24, 1.20]
Bryce 2018	0.305	0.269	29	27	0.8%	0.30 [-0.22, 0.83]
Burda 1994	0.56	0.249	40	29	0.9%	0.56 [0.07, 1.05]
Byrne 2013	0.52	0.367	14	17	0.5%	0.52 [-0.20, 1.24]
Cassetta 2019	0.507	0.274	28	27	0.8%	0.51 [-0.03, 1.04]
Cassetta 2019-2	0.574	0.276	28	27	0.7%	0.57 [0.03, 1.11]
D'Amato 2011	0.185	0.228	39	38	1.0%	0.18 [-0.26, 0.63]
D'Souza 2013	0.076	0.298	22	23	0.7%	0.08 [-0.51, 0.66]
Dickinson 2010	0.38	0.26	34	27	0.8%	0.38 [-0.13, 0.89]
Donohoe 2018	0.128	0.271	25	30	0.8%	0.13 [-0.40, 0.66]
Drake 2014	-0.082	0.258	30	30	0.8%	-0.08 [-0.59, 0.42]
Fan 2017	0.005	0.417	12	11	0.4%	0.01 [-0.81, 0.82]
Farreny 2012	0.292	0.277	29	24	0.7%	0.29 [-0.25, 0.83]
Fernandez-Gonzalo 2015	0.2	0.317	21	19	0.6%	0.20 [-0.42, 0.82]
Fisher 2009-2016	0.357	0.217	46	41	1.1%	0.36 [-0.07, 0.78]
Fisher 2015	0.429	0.184	63	58	1.3%	0.43 [0.07, 0.79]
Fiszdon 2016	0.252	0.246	50	25	0.9%	0.25 [-0.23, 0.73]
Garcia 2003	-0.124	0.45	11	9	0.3%	-0.12 [-1.01, 0.76]
Garcia-Fernandez 2019	-0.032	0.219	36	50	1.0%	-0.03 [-0.46, 0.40]
Garrido 2013	0.594	0.303	31	18	0.6%	0.59 [0.00, 1.19]
Gaweda 2015	0.103	0.302	23	21	0.6%	0.10 [-0.49, 0.69]
Gharaeipour 2012	0.694	0.318	21	21	0.6%	0.69 [0.07, 1.32]
Gohar 2013	0.076	0.309	22	20	0.6%	0.08 [-0.53, 0.68]
Gomar 2015	0.155	0.231	39	36	1.0%	0.15 [-0.30, 0.61]
Gomar 2015-2	-0.027	0.243	32	36	0.9%	-0.03 [-0.50, 0.45]
Gordon 2018	0.225	0.351	18	15	0.5%	0.23 [-0.46, 0.91]
Greig 2007	0.186	0.237	38	34	0.9%	0.19 [-0.28, 0.65]
Habel 2010	0.742	0.463	10	10	0.3%	0.74 [-0.17, 1.65]
Hegde 2012	0.914	0.383	13	18	0.4%	0.91 [0.16, 1.66]
Hermanutz 1991	0.149	0.448	10	10	0.3%	0.15 [-0.73, 1.03]
Hermanutz 1991-2	-0.007	0.447	10	10	0.3%	-0.01 [-0.88, 0.87]
Hodge 2010	0.226	0.319	22	18	0.6%	0.23 [-0.40, 0.85]
Hooker 2012	0.274	0.429	11	11	0.4%	0.27 [-0.57, 1.11]
Horan 2009	-0.183	0.36	15	16	0.5%	-0.18 [-0.89, 0.52]
Horan 2011	-0.015	0.324	19	19	0.6%	-0.01 [-0.65, 0.62]
Horan 2011-2	0.774	0.352	16	19	0.5%	0.77 [0.08, 1.46]
Horan 2011-3	0.181	0.353	14	19	0.5%	0.18 [-0.51, 0.87]
Horan 2018	-0.146	0.214	41	47	1.1%	-0.15 [-0.57, 0.27]
Horan 2018-2	0.141	0.207	47	47	1.1%	0.14 [-0.26, 0.55]
Jahshan 2019	-0.046	0.295	29	19	0.7%	-0.05 [-0.62, 0.53]
Jahshan 2019-2	0.014	0.285	35	19	0.7%	0.01 [-0.54, 0.57]
Kanie 2019	-0.006	0.25	32	32	0.9%	-0.01 [-0.50, 0.48]
Kantrowitz 2016	0.129	0.183	56	64	1.3%	0.13 [-0.23, 0.49]
Katsumi 2019	2.009	0.373	22	22	0.5%	2.01 [1.28, 2.74]
Keefe 2012	0.172	0.293	25	22	0.7%	0.17 [-0.40, 0.75]
Kurtz 2007	-0.03	0.31	23	19	0.6%	-0.03 [-0.64, 0.58]
Kurtz 2015	0.032	0.25	32	32	0.9%	0.03 [-0.46, 0.52]
Lado-Colesido 2019	0.889	0.297	26	24	0.7%	0.89 [0.31, 1.47]
Lu 2012	1.067	0.194	60	62	1.2%	1.07 [0.69, 1.45]
Mahncke 2019	-0.117	0.169	68	73	1.4%	-0.12 [-0.45, 0.21]
Mak 2013	-0.091	0.222	41	40	1.0%	-0.09 [-0.53, 0.34]
Man 2012	0.065	0.265	27	30	0.8%	0.07 [-0.45, 0.58]
Man 2012-2	0.105	0.277	23	30	0.7%	0.10 [-0.44, 0.65]
Marono Souto 2018	0.536	0.263	30	30	0.8%	0.54 [0.02, 1.05]
Matsuda 2018	0.267	0.255	31	31	0.8%	0.27 [-0.23, 0.77]
Matsui 2009	0.079	0.45	11	9	0.3%	0.08 [-0.80, 0.96]
Medalia 1998	0.223	0.273	27	27	0.8%	0.22 [-0.31, 0.76]
Medalia 2000	-0.006	0.333	18	18	0.5%	-0.01 [-0.66, 0.65]
Medalia 2000-2	-0.141	0.334	18	18	0.5%	-0.14 [-0.80, 0.51]
Meichenbaum 1973	1.637	0.753	5	5	0.1%	1.64 [0.16, 3.11]
Mendella 2015	0.641	0.402	16	11	0.4%	0.64 [-0.15, 1.43]
Morimoto 2018	0.368	0.363	16	15	0.5%	0.37 [-0.34, 1.08]
Moritz 2011	0.733	0.345	18	18	0.5%	0.73 [0.06, 1.41]
Mueller 2015	0.266	0.161	81	75	1.5%	0.27 [-0.05, 0.58]
Mueller 2017	0.369	0.259	28	33	0.8%	0.37 [-0.14, 0.88]
O'Reilly 2019	0.207	0.249	32	33	0.9%	0.21 [-0.28, 0.70]
Ochoa 2017	0.084	0.213	48	41	1.1%	0.08 [-0.33, 0.50]
Omiya 2016	0.096	0.486	8	9	0.3%	0.10 [-0.86, 1.05]
Penades 2006	1.026	0.337	20	20	0.5%	1.03 [0.37, 1.69]
Penades 2013	0.076	0.338	17	18	0.5%	0.08 [-0.59, 0.74]
Penades 2018	1.077	0.256	35	35	0.8%	1.08 [0.58, 1.58]
Pijnenborg 2019	-0.165	0.196	50	55	1.2%	-0.17 [-0.55, 0.22]
Pontes 2013	-0.059	0.486	9	8	0.3%	-0.06 [-1.01, 0.89]
Popova 2014	0.18	0.325	19	19	0.6%	0.18 [-0.46, 0.82]
Popova 2014-2	0.022	0.324	19	19	0.6%	0.02 [-0.61, 0.66]
Rakitzi 2016	0.376	0.337	19	17	0.5%	0.38 [-0.28, 1.04]
Ramsay 2017	0.147	0.394	14	12	0.4%	0.15 [-0.63, 0.92]
Rass 2012	-0.023	0.343	17	17	0.5%	-0.02 [-0.70, 0.65]
Rass 2012-2	-0.084	0.399	17	10	0.4%	-0.08 [-0.87, 0.70]
Reeder 2017	0.249	0.208	46	47	1.1%	0.25 [-0.16, 0.66]
Roberts 2014	0.124	0.258	30	30	0.8%	0.12 [-0.38, 0.63]
Roncone 2004	0.581	0.458	10	10	0.3%	0.58 [-0.32, 1.48]
Royer 2012	0.487	0.307	28	18	0.6%	0.49 [-0.11, 1.09]
Sachs 2012	-0.164	0.325	20	18	0.6%	-0.16 [-0.80, 0.47]
Sartory 2005	0.328	0.311	21	21	0.6%	0.33 [-0.28, 0.94]
Sevos 2018	0.217	0.361	16	15	0.5%	0.22 [-0.49, 0.92]
Silverstein 2005	0.281	0.366	18	13	0.5%	0.28 [-0.44, 1.00]
Tan 2016	0.33	0.197	52	52	1.2%	0.33 [-0.06, 0.72]
Tan 2019	0.192	0.118	196	115	2.0%	0.19 [-0.04, 0.42]
Tao 2015	0.832	0.225	44	42	1.0%	0.83 [0.39, 1.27]
Tas 2012	0.585	0.308	19	26	0.6%	0.58 [-0.02, 1.19]
Thomas 2018	0.197	0.296	24	22	0.7%	0.20 [-0.38, 0.78]
Twamley 2008-2012	0.295	0.243	38	31	0.9%	0.29 [-0.18, 0.77]
Van Oosterhout 2014	-0.283	0.191	51	60	1.2%	-0.28 [-0.66, 0.09]
Vaskinn 2019	-0.175	0.317	20	20	0.6%	-0.17 [-0.80, 0.45]
Vidarsdottir 2019	0.317	0.288	25	24	0.7%	0.32 [-0.25, 0.88]
Wolwer 2005	0.624	0.282	28	25	0.7%	0.62 [0.07, 1.18]
Wolwer 2005-2	0.372	0.288	24	25	0.7%	0.37 [-0.19, 0.94]
Wykes 1999	0.177	0.349	17	16	0.5%	0.18 [-0.51, 0.86]
Wykes 2007a	0.13	0.317	21	19	0.6%	0.13 [-0.49, 0.75]
Wykes 2007b	0.211	0.227	39	39	1.0%	0.21 [-0.23, 0.66]
Zimmer 2007	0.401	0.282	20	36	0.7%	0.40 [-0.15, 0.95]
**Subtotal (95% CI)**			**3123**	**2953**	**77.6%**	**0.26 [0.20, 0.32]**
Heterogeneity: Tau² = 0.03; Chi² = 150.61, df = 106 (P = 0.003); I² = 30%						
Test for overall effect: Z = 8.10 (P < 0.00001)						
**2.37.3 All core elements (integrated rehabilitation)**						
Aloi 2018	0.483	0.317	21	20	0.6%	0.48 [-0.14, 1.10]
Bowie 2012-2	0.68	0.244	36	35	0.9%	0.68 [0.20, 1.16]
Cavallaro 2009	0.215	0.219	50	36	1.0%	0.21 [-0.21, 0.64]
Choi 2006	0.621	0.487	10	8	0.3%	0.62 [-0.33, 1.58]
Choi 2017	0.315	0.331	18	19	0.6%	0.32 [-0.33, 0.96]
Eack 2009	0.187	0.264	31	27	0.8%	0.19 [-0.33, 0.70]
Hadas-Lidor 2001	0.678	0.27	29	29	0.8%	0.68 [0.15, 1.21]
Hogarty 2004	0.255	0.196	61	46	1.2%	0.26 [-0.13, 0.64]
Iwata 2017	0.441	0.271	28	28	0.8%	0.44 [-0.09, 0.97]
Kidd 2014	0.196	0.365	18	13	0.5%	0.20 [-0.52, 0.91]
Kukla 2018	0.531	0.304	23	22	0.6%	0.53 [-0.06, 1.13]
Lee 2013	0.681	0.266	30	30	0.8%	0.68 [0.16, 1.20]
Lindenmayer 2008	0.158	0.238	41	31	0.9%	0.16 [-0.31, 0.62]
McGurk 2005	0.585	0.308	23	21	0.6%	0.58 [-0.02, 1.19]
McGurk 2016	0.268	0.283	28	23	0.7%	0.27 [-0.29, 0.82]
Ojeda 2012	0.64	0.224	44	40	1.0%	0.64 [0.20, 1.08]
Ostergaard 2014	0.284	0.186	60	57	1.3%	0.28 [-0.08, 0.65]
Pena 2016	0.347	0.201	52	49	1.2%	0.35 [-0.05, 0.74]
Puig 2014	0.408	0.286	25	25	0.7%	0.41 [-0.15, 0.97]
Sanchez 2014	0.643	0.226	36	48	1.0%	0.64 [0.20, 1.09]
Spaulding 1999	0.08	0.211	48	42	1.1%	0.08 [-0.33, 0.49]
Tan 2013	0.925	0.252	36	34	0.9%	0.93 [0.43, 1.42]
Ueland 2004	0.326	0.396	14	12	0.4%	0.33 [-0.45, 1.10]
Vauth 2005	0.411	0.238	37	35	0.9%	0.41 [-0.06, 0.88]
Vauth 2005-2	0.24	0.251	37	28	0.9%	0.24 [-0.25, 0.73]
Vita 2011a	0.337	0.274	26	28	0.8%	0.34 [-0.20, 0.87]
Vita 2011a-2	0.288	0.264	30	28	0.8%	0.29 [-0.23, 0.81]
Vita 2011b	0.361	0.363	16	15	0.5%	0.36 [-0.35, 1.07]
**Subtotal (95% CI)**			**908**	**829**	**22.4%**	**0.40 [0.30, 0.49]**
Heterogeneity: Tau² = 0.00; Chi² = 17.97, df = 27 (P = 0.90); I² = 0%						
Test for overall effect: Z = 8.19 (P < 0.00001)						
**Total (95% CI)**			**4031**	**3782**	**100.0%**	**0.29 [0.24, 0.34]**
Heterogeneity: Tau² = 0.02; Chi² = 175.71, df = 134 (P = 0.009); I² = 24%						
Test for overall effect: Z = 10.78 (P < 0.00001)						
Test for subgroup differences: Chi² = 5.66, df = 1 (P = 0.02), I² = 82.3%						

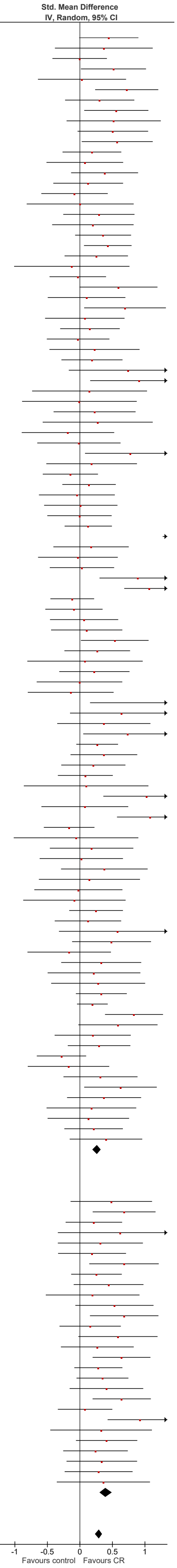

© 2021 Vita et al. *JAMA Psychiatry.*

eFigure 4. Subgroup analysis for the effects of interventions including all core elements of cognitive remediation (global functioning)

Study or Subgroup	Std. Mean Difference	SE	CR Total	Control Total	Weight	Std. Mean Difference IV, Random, 95% CI
**2.38.1 Not enough core elements**						
Ahmed 2015	0.286	0.228	42	36	1.3%	0.29 [-0.16, 0.73]
Ahuir 2018	0.006	0.378	14	14	0.6%	0.01 [-0.73, 0.75]
Au 2015	-0.07	0.211	45	45	1.4%	-0.07 [-0.48, 0.34]
Bowie 2012	-0.15	0.238	36	35	1.2%	-0.15 [-0.62, 0.32]
Briki 2014	0.049	0.283	25	25	1.0%	0.05 [-0.51, 0.60]
Bryce 2018	-0.072	0.268	29	27	1.0%	-0.07 [-0.60, 0.45]
Byrne 2013	0.71	0.373	14	17	0.6%	0.71 [-0.02, 1.44]
Cassetta 2019	0.415	0.273	28	27	1.0%	0.41 [-0.12, 0.95]
Cassetta 2019-2	0.49	0.274	28	27	1.0%	0.49 [-0.05, 1.03]
D'Amato 2011	0.073	0.228	39	38	1.3%	0.07 [-0.37, 0.52]
D'Souza 2013	0.137	0.299	22	23	0.9%	0.14 [-0.45, 0.72]
Dickinson 2010	-0.183	0.258	34	27	1.1%	-0.18 [-0.69, 0.32]
Donohoe 2018	0.488	0.275	25	30	1.0%	0.49 [-0.05, 1.03]
Drake 2014	-0.323	0.26	30	30	1.1%	-0.32 [-0.83, 0.19]
Farreny 2012	0.53	0.281	29	24	1.0%	0.53 [-0.02, 1.08]
Fernandez-Gonzalo 2015	0.074	0.317	21	19	0.8%	0.07 [-0.55, 0.70]
Fisher 2009-2016	-0.166	0.215	0	0	1.4%	-0.17 [-0.59, 0.26]
Fisher 2015	-0.066	0.182	63	58	1.6%	-0.07 [-0.42, 0.29]
Fiszdon 2016	0.026	0.245	50	25	1.2%	0.03 [-0.45, 0.51]
Garcia 2003	0.251	0.451	11	9	0.5%	0.25 [-0.63, 1.13]
Garcia-Fernandez 2019	0.041	0.219	36	50	1.3%	0.04 [-0.39, 0.47]
Garrido 2013	1.077	0.316	31	18	0.8%	1.08 [0.46, 1.70]
Gomar 2015	-0.038	0.231	39	36	1.2%	-0.04 [-0.49, 0.41]
Gomar 2015-2	0.223	0.244	32	36	1.2%	0.22 [-0.26, 0.70]
Gordon 2018	0.244	0.351	18	15	0.7%	0.24 [-0.44, 0.93]
Hermanutz 1991	-0.088	0.447	10	10	0.5%	-0.09 [-0.96, 0.79]
Hermanutz 1991-2	-0.099	0.448	10	10	0.5%	-0.10 [-0.98, 0.78]
Hooker 2012	-0.295	0.429	11	11	0.5%	-0.29 [-1.14, 0.55]
Horan 2011	0.382	0.328	19	19	0.8%	0.38 [-0.26, 1.02]
Horan 2011-2	0.372	0.342	16	19	0.7%	0.37 [-0.30, 1.04]
Horan 2011-3	0.235	0.353	14	19	0.7%	0.23 [-0.46, 0.93]
Horan 2018	0.143	0.214	41	47	1.4%	0.14 [-0.28, 0.56]
Horan 2018-2	-0.026	0.206	47	47	1.4%	-0.03 [-0.43, 0.38]
Jahshan 2019	0.202	0.296	29	19	0.9%	0.20 [-0.38, 0.78]
Jahshan 2019-2	0.195	0.286	35	19	1.0%	0.20 [-0.37, 0.76]
Kanie 2019	0.11	0.25	32	32	1.1%	0.11 [-0.38, 0.60]
Kantrowitz 2016	0.2	0.183	56	64	1.6%	0.20 [-0.16, 0.56]
Katsumi 2019	0.336	0.304	22	22	0.9%	0.34 [-0.26, 0.93]
Keefe 2012	0.087	0.292	25	22	0.9%	0.09 [-0.49, 0.66]
Klingberg 2011	-0.204	0.143	99	99	2.0%	-0.20 [-0.48, 0.08]
Kurtz 2015	0.134	0.25	32	32	1.1%	0.13 [-0.36, 0.62]
Lu 2012	0.732	0.187	60	62	1.6%	0.73 [0.37, 1.10]
Mahncke 2019	0.02	0.169	68	73	1.7%	0.02 [-0.31, 0.35]
Matsuda 2018	0.095	0.254	31	31	1.1%	0.10 [-0.40, 0.59]
Medalia 2000	-0.577	0.341	18	18	0.7%	-0.58 [-1.25, 0.09]
Medalia 2000-2	1.486	0.379	18	18	0.6%	1.49 [0.74, 2.23]
Mendella 2015	0.043	0.392	16	11	0.6%	0.04 [-0.73, 0.81]
Morimoto 2018	0.15	0.36	16	15	0.7%	0.15 [-0.56, 0.86]
Mueller 2015	0.27	0.161	81	75	1.8%	0.27 [-0.05, 0.59]
Mueller 2017	-0.094	0.257	28	33	1.1%	-0.09 [-0.60, 0.41]
O'Reilly 2019	-0.042	0.248	32	33	1.1%	-0.04 [-0.53, 0.44]
Ochoa 2017	0.013	0.213	48	41	1.4%	0.01 [-0.40, 0.43]
Omiya 2016	0.813	0.508	8	9	0.4%	0.81 [-0.18, 1.81]
Penades 2006	0.737	0.327	20	20	0.8%	0.74 [0.10, 1.38]
Penades 2018	0.454	0.242	35	35	1.2%	0.45 [-0.02, 0.93]
Pijnenborg 2019	-0.19	0.196	50	55	1.5%	-0.19 [-0.57, 0.19]
Popova 2014	0.152	0.325	19	19	0.8%	0.15 [-0.48, 0.79]
Popova 2014-2	0.276	0.326	19	19	0.8%	0.28 [-0.36, 0.91]
Rakitzi 2016	0.196	0.335	19	17	0.8%	0.20 [-0.46, 0.85]
Ramsay 2017	-0.319	0.396	14	12	0.6%	-0.32 [-1.10, 0.46]
Reeder 2017	0.633	0.213	46	47	1.4%	0.63 [0.22, 1.05]
Roberts 2014	0.077	0.258	30	30	1.1%	0.08 [-0.43, 0.58]
Sachs 2012	0.306	0.327	20	18	0.8%	0.31 [-0.33, 0.95]
Silverstein 2005	0.342	0.367	18	13	0.7%	0.34 [-0.38, 1.06]
Silverstein 2009	0.471	0.23	44	35	1.3%	0.47 [0.02, 0.92]
Tan 2016	0.366	0.198	52	52	1.5%	0.37 [-0.02, 0.75]
Tan 2019	0.019	0.117	196	115	2.2%	0.02 [-0.21, 0.25]
Tas 2012	0.677	0.311	19	26	0.8%	0.68 [0.07, 1.29]
Twamley 2008-2012	0.121	0.242	38	31	1.2%	0.12 [-0.35, 0.60]
Vaskinn 2019	-0.054	0.316	20	20	0.8%	-0.05 [-0.67, 0.57]
Vidarsdottir 2019	-0.029	0.286	25	24	1.0%	-0.03 [-0.59, 0.53]
Wykes 1999	0.284	0.35	17	16	0.7%	0.28 [-0.40, 0.97]
Wykes 2007a	0.011	0.317	21	19	0.8%	0.01 [-0.61, 0.63]
Wykes 2007b	-0.08	0.227	39	39	1.3%	-0.08 [-0.52, 0.36]
Zimmer 2007	0.766	0.288	20	36	0.9%	0.77 [0.20, 1.33]
**Subtotal (95% CI)**			**2444**	**2299**	**77.7%**	**0.16 [0.09, 0.23]**
Heterogeneity: Tau² = 0.02; Chi² = 101.51, df = 74 (P = 0.02); I² = 27%						
Test for overall effect: Z = 4.51 (P < 0.00001)						
**2.38.3 All core elements (integrated rehabilitation)**						
Aloi 2018	0.444	0.316	21	20	0.8%	0.44 [-0.18, 1.06]
Bowie 2012-2	0.12	0.238	36	35	1.2%	0.12 [-0.35, 0.59]
Cavallaro 2009	0.205	0.219	50	36	1.3%	0.20 [-0.22, 0.63]
Eack 2009	1.508	0.299	31	27	0.9%	1.51 [0.92, 2.09]
Galderisi 2010	0.754	0.297	26	23	0.9%	0.75 [0.17, 1.34]
Hogarty 2004	0.3	0.196	61	46	1.5%	0.30 [-0.08, 0.68]
Iwata 2017	0.548	0.272	28	28	1.0%	0.55 [0.01, 1.08]
Kukla 2018	0.24	0.299	23	22	0.9%	0.24 [-0.35, 0.83]
Lee 2013	0.736	0.236	30	30	1.2%	0.74 [0.27, 1.20]
Ojeda 2012	0.063	0.219	44	40	1.3%	0.06 [-0.37, 0.49]
Ostergaard 2014	-0.007	0.185	60	57	1.6%	-0.01 [-0.37, 0.36]
Pena 2016	0.443	0.202	52	49	1.4%	0.44 [0.05, 0.84]
Puig 2014	0.422	0.286	25	25	1.0%	0.42 [-0.14, 0.98]
Sanchez 2014	0.526	0.224	36	48	1.3%	0.53 [0.09, 0.97]
Spaulding 1999	0.454	0.214	48	42	1.4%	0.45 [0.03, 0.87]
Ueland 2004	0.36	0.397	14	12	0.6%	0.36 [-0.42, 1.14]
Ventura 2019	0.441	0.226	39	41	1.3%	0.44 [-0.00, 0.88]
Vita 2011a	0.418	0.275	26	28	1.0%	0.42 [-0.12, 0.96]
Vita 2011a-2	0.818	0.274	30	28	1.0%	0.82 [0.28, 1.36]
Vita 2011b	0.511	0.366	16	15	0.7%	0.51 [-0.21, 1.23]
**Subtotal (95% CI)**			**696**	**652**	**22.3%**	**0.43 [0.30, 0.57]**
Heterogeneity: Tau² = 0.04; Chi² = 30.26, df = 19 (P = 0.05); I² = 37%						
Test for overall effect: Z = 6.13 (P < 0.00001)						
**Total (95% CI)**			**3140**	**2951**	**100.0%**	**0.22 [0.16, 0.29]**
Heterogeneity: Tau² = 0.04; Chi² = 150.29, df = 94 (P = 0.0002); I² = 37%						
Test for overall effect: Z = 6.63 (P < 0.00001)						
Test for subgroup differences: Chi² = 12.08, df = 1 (P = 0.0005), I² = 91.7%						

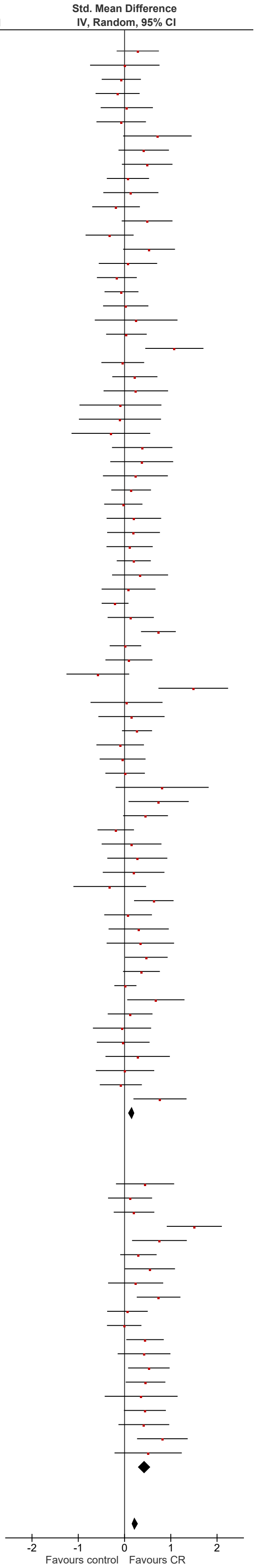

© 2021 Vita et al. *JAMA Psychiatry.*

eFigure 5. Sensitivity analysis: subgroup analysis in eFigure 3 including only methodological adequate studies (global cognition)

Study or Subgroup	Std. Mean Difference	SE	CR Total	Control Total	Weight	Std. Mean Difference IV, Random, 95% CI
**2.41.1 Not enough core elements**						
Ahmed 2015	0.444	0.23	42	36	1.6%	0.44 [-0.01, 0.89]
Au 2015	-0.003	0.211	45	45	1.8%	-0.00 [-0.42, 0.41]
Bellucci 2003	0.033	0.343	17	17	0.8%	0.03 [-0.64, 0.71]
Bowie 2012	0.72	0.245	36	35	1.4%	0.72 [0.24, 1.20]
Bryce 2018	0.305	0.269	29	27	1.2%	0.30 [-0.22, 0.83]
Cassetta 2019	0.507	0.274	28	27	1.2%	0.51 [-0.03, 1.04]
Cassetta 2019-2	0.574	0.276	28	27	1.2%	0.57 [0.03, 1.11]
D'Amato 2011	0.185	0.228	39	38	1.6%	0.18 [-0.26, 0.63]
Dickinson 2010	0.38	0.26	34	27	1.3%	0.38 [-0.13, 0.89]
Donohoe 2018	0.128	0.271	25	30	1.2%	0.13 [-0.40, 0.66]
Drake 2014	-0.082	0.258	30	30	1.3%	-0.08 [-0.59, 0.42]
Farreny 2012	0.292	0.277	29	24	1.2%	0.29 [-0.25, 0.83]
Fisher 2009-2016	0.357	0.217	46	41	1.8%	0.36 [-0.07, 0.78]
Fisher 2015	0.429	0.184	63	58	2.3%	0.43 [0.07, 0.79]
Garcia-Fernandez 2019	-0.032	0.219	36	50	1.7%	-0.03 [-0.46, 0.40]
Garrido 2013	0.594	0.303	31	18	1.0%	0.59 [0.00, 1.19]
Gaweda 2015	0.103	0.302	23	21	1.0%	0.10 [-0.49, 0.69]
Gomar 2015	0.155	0.231	39	36	1.6%	0.15 [-0.30, 0.61]
Gomar 2015-2	-0.027	0.243	32	36	1.5%	-0.03 [-0.50, 0.45]
Hermanutz 1991	0.149	0.448	10	10	0.5%	0.15 [-0.73, 1.03]
Hermanutz 1991-2	-0.007	0.447	10	10	0.5%	-0.01 [-0.88, 0.87]
Hooker 2012	0.274	0.429	11	11	0.5%	0.27 [-0.57, 1.11]
Horan 2011	-0.015	0.324	19	19	0.9%	-0.01 [-0.65, 0.62]
Horan 2011-2	0.774	0.352	16	19	0.8%	0.77 [0.08, 1.46]
Horan 2011-3	0.181	0.353	14	19	0.8%	0.18 [-0.51, 0.87]
Horan 2018	-0.146	0.214	41	47	1.8%	-0.15 [-0.57, 0.27]
Horan 2018-2	0.141	0.207	47	47	1.9%	0.14 [-0.26, 0.55]
Jahshan 2019	-0.046	0.295	29	19	1.1%	-0.05 [-0.62, 0.53]
Jahshan 2019-2	0.014	0.285	35	19	1.1%	0.01 [-0.54, 0.57]
Kanie 2019	-0.006	0.25	32	32	1.4%	-0.01 [-0.50, 0.48]
Kantrowitz 2016	0.129	0.183	56	64	2.3%	0.13 [-0.23, 0.49]
Keefe 2012	0.172	0.293	25	22	1.1%	0.17 [-0.40, 0.75]
Kurtz 2015	0.032	0.25	32	32	1.4%	0.03 [-0.46, 0.52]
Mahncke 2019	-0.117	0.169	68	73	2.5%	-0.12 [-0.45, 0.21]
Matsuda 2018	0.267	0.255	31	31	1.4%	0.27 [-0.23, 0.77]
Meichenbaum 1973	1.637	0.753	5	5	0.2%	1.64 [0.16, 3.11]
Moritz 2011	0.733	0.345	18	18	0.8%	0.73 [0.06, 1.41]
Mueller 2015	0.266	0.161	81	75	2.7%	0.27 [-0.05, 0.58]
Mueller 2017	0.369	0.259	28	33	1.3%	0.37 [-0.14, 0.88]
O'Reilly 2019	0.207	0.249	32	33	1.4%	0.21 [-0.28, 0.70]
Ochoa 2017	0.084	0.213	48	41	1.8%	0.08 [-0.33, 0.50]
Penades 2006	1.026	0.337	20	20	0.8%	1.03 [0.37, 1.69]
Penades 2013	0.076	0.338	17	18	0.8%	0.08 [-0.59, 0.74]
Penades 2018	1.077	0.256	35	35	1.3%	1.08 [0.58, 1.58]
Pijnenborg 2019	-0.165	0.196	50	55	2.1%	-0.17 [-0.55, 0.22]
Pontes 2013	-0.059	0.486	9	8	0.4%	-0.06 [-1.01, 0.89]
Rakitzi 2016	0.376	0.337	19	17	0.8%	0.38 [-0.28, 1.04]
Rass 2012	-0.023	0.343	17	17	0.8%	-0.02 [-0.70, 0.65]
Rass 2012-2	-0.084	0.399	17	10	0.6%	-0.08 [-0.87, 0.70]
Reeder 2017	0.249	0.208	46	47	1.9%	0.25 [-0.16, 0.66]
Tan 2016	0.33	0.197	52	52	2.0%	0.33 [-0.06, 0.72]
Tan 2019	0.192	0.118	196	115	3.9%	0.19 [-0.04, 0.42]
Van Oosterhout 2014	-0.283	0.191	51	60	2.1%	-0.28 [-0.66, 0.09]
Vidarsdottir 2019	0.317	0.288	25	24	1.1%	0.32 [-0.25, 0.88]
Wykes 1999	0.177	0.349	17	16	0.8%	0.18 [-0.51, 0.86]
Wykes 2007b	0.211	0.227	39	39	1.6%	0.21 [-0.23, 0.66]
Zimmer 2007	0.401	0.282	20	36	1.1%	0.40 [-0.15, 0.95]
**Subtotal (95% CI)**			**1970**	**1871**	**77.2%**	**0.21 [0.13, 0.28]**
Heterogeneity: Tau² = 0.01; Chi² = 67.17, df = 56 (P = 0.15); I² = 17%						
Test for overall effect: Z = 5.60 (P < 0.00001)						
**2.41.3 All core elements (integrated rehabilitation)**						
Bowie 2012-2	0.68	0.244	36	35	1.5%	0.68 [0.20, 1.16]
Choi 2017	0.315	0.331	18	19	0.9%	0.32 [-0.33, 0.96]
Iwata 2017	0.441	0.271	28	28	1.2%	0.44 [-0.09, 0.97]
Kidd 2014	0.196	0.365	18	13	0.7%	0.20 [-0.52, 0.91]
Kukla 2018	0.531	0.304	23	22	1.0%	0.53 [-0.06, 1.13]
Lee 2013	0.681	0.266	30	30	1.3%	0.68 [0.16, 1.20]
McGurk 2016	0.268	0.283	28	23	1.1%	0.27 [-0.29, 0.82]
Ostergaard 2014	0.284	0.186	60	57	2.2%	0.28 [-0.08, 0.65]
Pena 2016	0.347	0.201	52	49	2.0%	0.35 [-0.05, 0.74]
Sanchez 2014	0.643	0.226	36	48	1.6%	0.64 [0.20, 1.09]
Spaulding 1999	0.08	0.211	48	42	1.8%	0.08 [-0.33, 0.49]
Tan 2013	0.925	0.252	36	34	1.4%	0.93 [0.43, 1.42]
Vauth 2005	0.411	0.238	37	35	1.5%	0.41 [-0.06, 0.88]
Vauth 2005-2	0.24	0.251	37	28	1.4%	0.24 [-0.25, 0.73]
Vita 2011a	0.337	0.274	26	28	1.2%	0.34 [-0.20, 0.87]
Vita 2011a-2	0.288	0.264	30	28	1.3%	0.29 [-0.23, 0.81]
Vita 2011b	0.361	0.363	16	15	0.7%	0.36 [-0.35, 1.07]
**Subtotal (95% CI)**			**559**	**534**	**22.8%**	**0.41 [0.29, 0.53]**
Heterogeneity: Tau² = 0.00; Chi² = 12.12, df = 16 (P = 0.74); I² = 0%						
Test for overall effect: Z = 6.68 (P < 0.00001)						
**Total (95% CI)**			**2529**	**2405**	**100.0%**	**0.25 [0.19, 0.32]**
Heterogeneity: Tau² = 0.01; Chi² = 88.43, df = 73 (P = 0.11); I² = 17%						
Test for overall effect: Z = 7.79 (P < 0.00001)						
Test for subgroup differences: Chi² = 8.16, df = 1 (P = 0.004), I² = 87.8%						

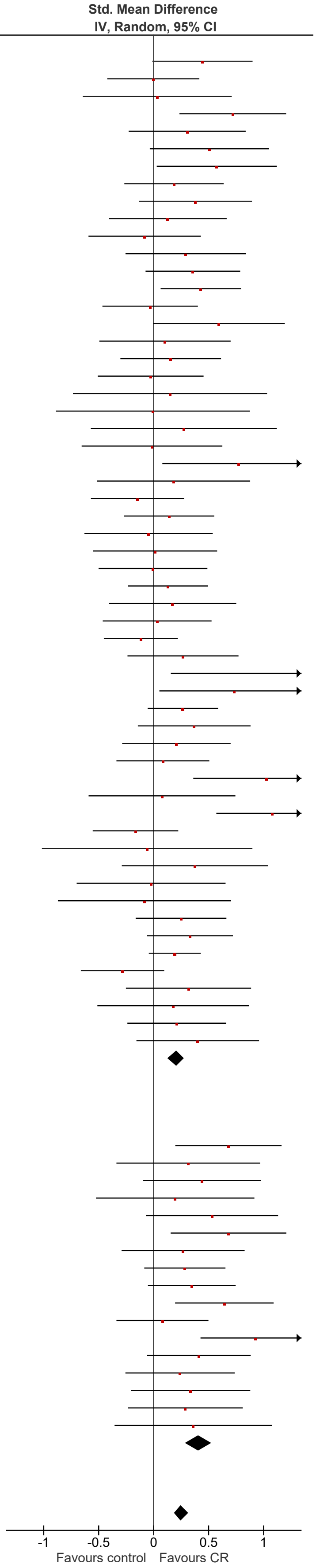

© 2021 Vita et al. *JAMA Psychiatry.*

eFigure 6. Sensitivity analysis: subgroup analysis in eFigure 4 including only methodological adequate studies (global functioning)

Study or Subgroup	Std. Mean Difference	SE	CR Total	Control Total	Weight	Std. Mean Difference IV, Random, 95% CI
**2.40.1 Not enough core elements**						
Ahmed 2015	0.286	0.228	42	36	1.8%	0.29 [-0.16, 0.73]
Au 2015	-0.07	0.211	45	45	2.0%	-0.07 [-0.48, 0.34]
Bowie 2012	-0.15	0.238	36	35	1.7%	-0.15 [-0.62, 0.32]
Briki 2014	0.049	0.283	25	25	1.3%	0.05 [-0.51, 0.60]
Bryce 2018	-0.072	0.268	29	27	1.4%	-0.07 [-0.60, 0.45]
Cassetta 2019	0.415	0.273	28	27	1.4%	0.41 [-0.12, 0.95]
Cassetta 2019-2	0.49	0.274	28	27	1.4%	0.49 [-0.05, 1.03]
D'Amato 2011	0.073	0.228	39	38	1.8%	0.07 [-0.37, 0.52]
Dickinson 2010	-0.183	0.258	34	27	1.5%	-0.18 [-0.69, 0.32]
Donohoe 2018	0.488	0.275	25	30	1.4%	0.49 [-0.05, 1.03]
Drake 2014	-0.323	0.26	30	30	1.5%	-0.32 [-0.83, 0.19]
Farreny 2012	0.53	0.281	29	24	1.3%	0.53 [-0.02, 1.08]
Fisher 2009-2016	-0.166	0.215	0	0	2.0%	-0.17 [-0.59, 0.26]
Fisher 2015	-0.066	0.182	63	58	2.4%	-0.07 [-0.42, 0.29]
Garcia-Fernandez 2019	0.041	0.219	36	50	1.9%	0.04 [-0.39, 0.47]
Garrido 2013	1.077	0.316	31	18	1.1%	1.08 [0.46, 1.70]
Gomar 2015	-0.038	0.231	39	36	1.8%	-0.04 [-0.49, 0.41]
Gomar 2015-2	0.223	0.244	32	36	1.6%	0.22 [-0.26, 0.70]
Hermanutz 1991	-0.088	0.447	10	10	0.6%	-0.09 [-0.96, 0.79]
Hermanutz 1991-2	-0.099	0.448	10	10	0.6%	-0.10 [-0.98, 0.78]
Hooker 2012	-0.295	0.429	11	11	0.7%	-0.29 [-1.14, 0.55]
Horan 2011	0.382	0.328	19	19	1.0%	0.38 [-0.26, 1.02]
Horan 2011-2	0.372	0.342	16	19	1.0%	0.37 [-0.30, 1.04]
Horan 2011-3	0.235	0.353	14	19	0.9%	0.23 [-0.46, 0.93]
Horan 2018	0.143	0.214	41	47	2.0%	0.14 [-0.28, 0.56]
Horan 2018-2	-0.026	0.206	47	47	2.1%	-0.03 [-0.43, 0.38]
Jahshan 2019	0.202	0.296	29	19	1.2%	0.20 [-0.38, 0.78]
Jahshan 2019-2	0.195	0.286	35	19	1.3%	0.20 [-0.37, 0.76]
Kanie 2019	0.11	0.25	32	32	1.6%	0.11 [-0.38, 0.60]
Kantrowitz 2016	0.2	0.183	56	64	2.4%	0.20 [-0.16, 0.56]
Keefe 2012	0.087	0.292	25	22	1.3%	0.09 [-0.49, 0.66]
Klingberg 2011	-0.204	0.143	99	99	3.1%	-0.20 [-0.48, 0.08]
Kurtz 2015	0.134	0.25	32	32	1.6%	0.13 [-0.36, 0.62]
Mahncke 2019	0.02	0.169	68	73	2.6%	0.02 [-0.31, 0.35]
Matsuda 2018	0.095	0.254	31	31	1.6%	0.10 [-0.40, 0.59]
Mueller 2015	0.27	0.161	81	75	2.8%	0.27 [-0.05, 0.59]
Mueller 2017	-0.094	0.257	28	33	1.5%	-0.09 [-0.60, 0.41]
O'Reilly 2019	-0.042	0.248	32	33	1.6%	-0.04 [-0.53, 0.44]
Ochoa 2017	0.013	0.213	48	41	2.0%	0.01 [-0.40, 0.43]
Penades 2006	0.737	0.327	20	20	1.1%	0.74 [0.10, 1.38]
Penades 2018	0.454	0.242	35	35	1.7%	0.45 [-0.02, 0.93]
Pijnenborg 2019	-0.19	0.196	50	55	2.2%	-0.19 [-0.57, 0.19]
Rakitzi 2016	0.196	0.335	19	17	1.0%	0.20 [-0.46, 0.85]
Reeder 2017	0.633	0.213	46	47	2.0%	0.63 [0.22, 1.05]
Tan 2016	0.366	0.198	52	52	2.2%	0.37 [-0.02, 0.75]
Tan 2019	0.019	0.117	196	115	3.6%	0.02 [-0.21, 0.25]
Vidarsdottir 2019	-0.029	0.286	25	24	1.3%	-0.03 [-0.59, 0.53]
Wykes 1999	0.284	0.35	17	16	0.9%	0.28 [-0.40, 0.97]
Wykes 2007b	-0.08	0.227	39	39	1.8%	-0.08 [-0.52, 0.36]
Zimmer 2007	0.766	0.288	20	36	1.3%	0.77 [0.20, 1.33]
**Subtotal (95% CI)**			**1874**	**1780**	**82.0%**	**0.12 [0.05, 0.19]**
Heterogeneity: Tau² = 0.01; Chi² = 60.78, df = 49 (P = 0.12); I² = 19%						
Test for overall effect: Z = 3.16 (P = 0.002)						
**2.40.3 All core elements (integrated rehabilitation)**						
Bowie 2012-2	0.12	0.238	36	35	1.7%	0.12 [-0.35, 0.59]
Iwata 2017	0.548	0.272	28	28	1.4%	0.55 [0.01, 1.08]
Kukla 2018	0.24	0.299	23	22	1.2%	0.24 [-0.35, 0.83]
Lee 2013	0.736	0.236	30	30	1.7%	0.74 [0.27, 1.20]
Ostergaard 2014	-0.007	0.185	60	57	2.4%	-0.01 [-0.37, 0.36]
Pena 2016	0.443	0.202	52	49	2.1%	0.44 [0.05, 0.84]
Sanchez 2014	0.526	0.224	36	48	1.9%	0.53 [0.09, 0.97]
Spaulding 1999	0.454	0.214	48	42	2.0%	0.45 [0.03, 0.87]
Vita 2011a	0.418	0.275	26	28	1.4%	0.42 [-0.12, 0.96]
Vita 2011a-2	0.818	0.274	30	28	1.4%	0.82 [0.28, 1.36]
Vita 2011b	0.511	0.366	16	15	0.9%	0.51 [-0.21, 1.23]
**Subtotal (95% CI)**			**385**	**382**	**18.0%**	**0.41 [0.26, 0.57]**
Heterogeneity: Tau² = 0.01; Chi² = 11.68, df = 10 (P = 0.31); I² = 14%						
Test for overall effect: Z = 5.19 (P < 0.00001)						
**Total (95% CI)**			**2259**	**2162**	**100.0%**	**0.18 [0.10, 0.25]**
Heterogeneity: Tau² = 0.02; Chi² = 86.14, df = 60 (P = 0.02); I² = 30%						
Test for overall effect: Z = 4.76 (P < 0.00001)						
Test for subgroup differences: Chi² = 11.00, df = 1 (P = 0.0009), I² = 90.9%						

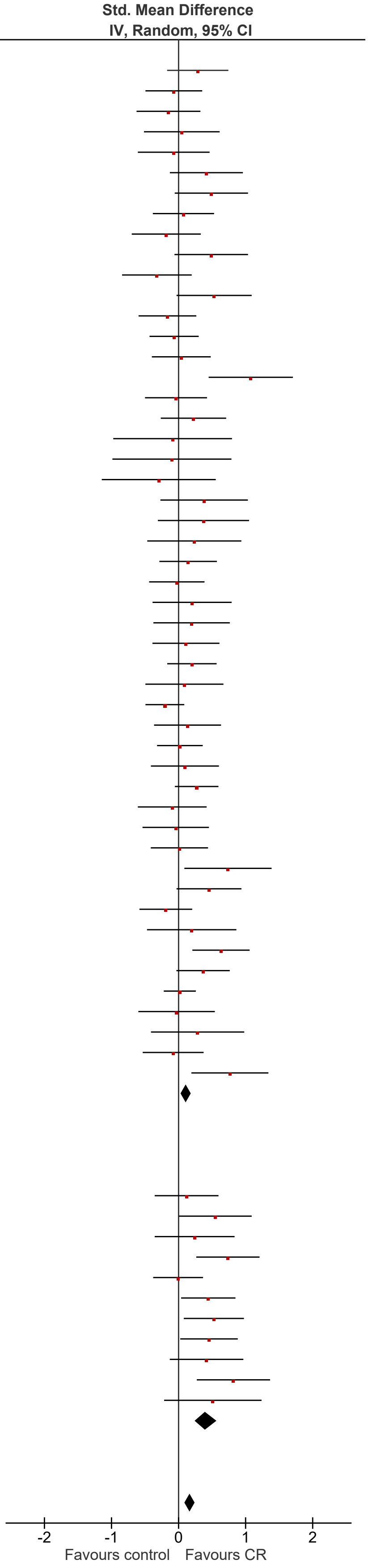

© 2021 Vita et al. *JAMA Psychiatry.*

eFigure 7. Meta-analytic scatter plot for global functioning (expressed as Cohen *d*) and duration of cognitive remediation (CR) programs (in weeks).

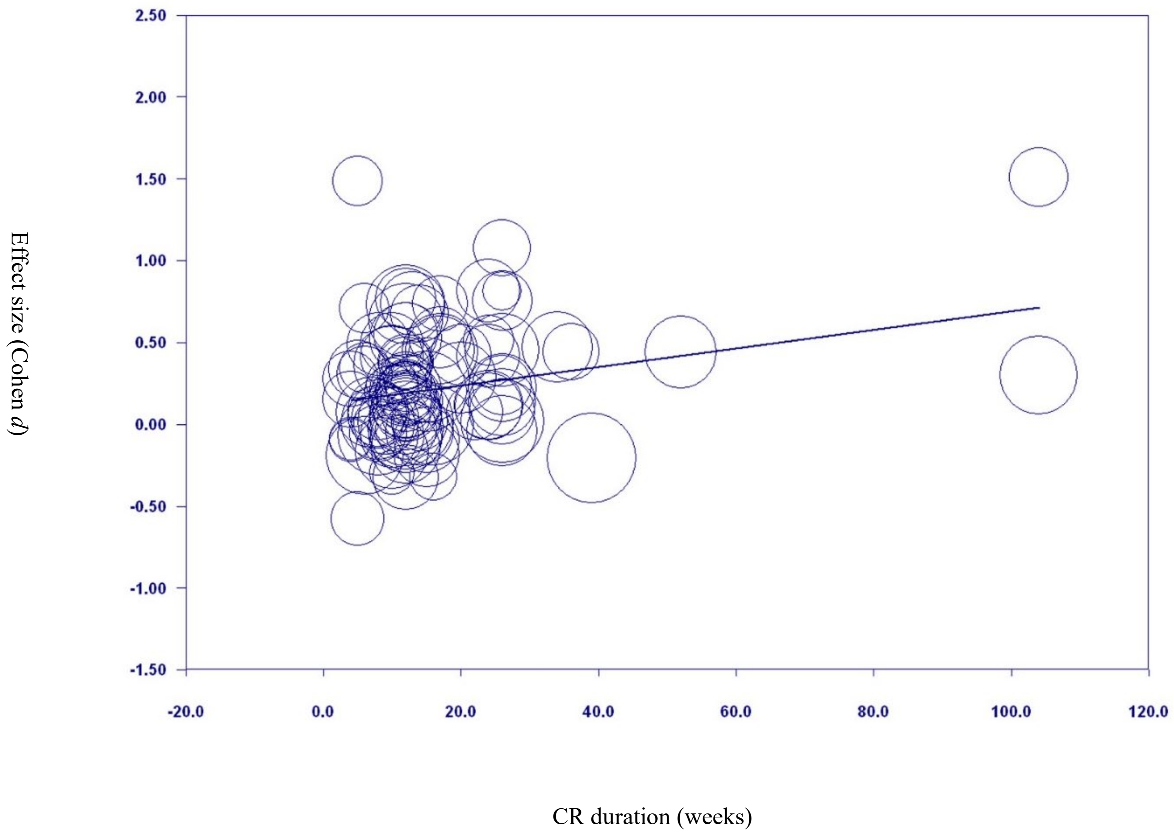

The figure shows the direction of effect size for global functioning as a function of CR duration. Circles are proportionate to study weight in the analysis.

	N	Coefficient	SE	z-test	95% CI (lower limit)	95% CI (upper limit)	p value
Slope	95	0.006	0.002	2.76	0.002	0.010	0.006
Intercept		0.122	0.049	2.50	0.026	0.218	0.013

© 2021 Vita et al. *JAMA Psychiatry.*

eFigure 8. Analysis of publication bias - Funnel plot for global cognition.

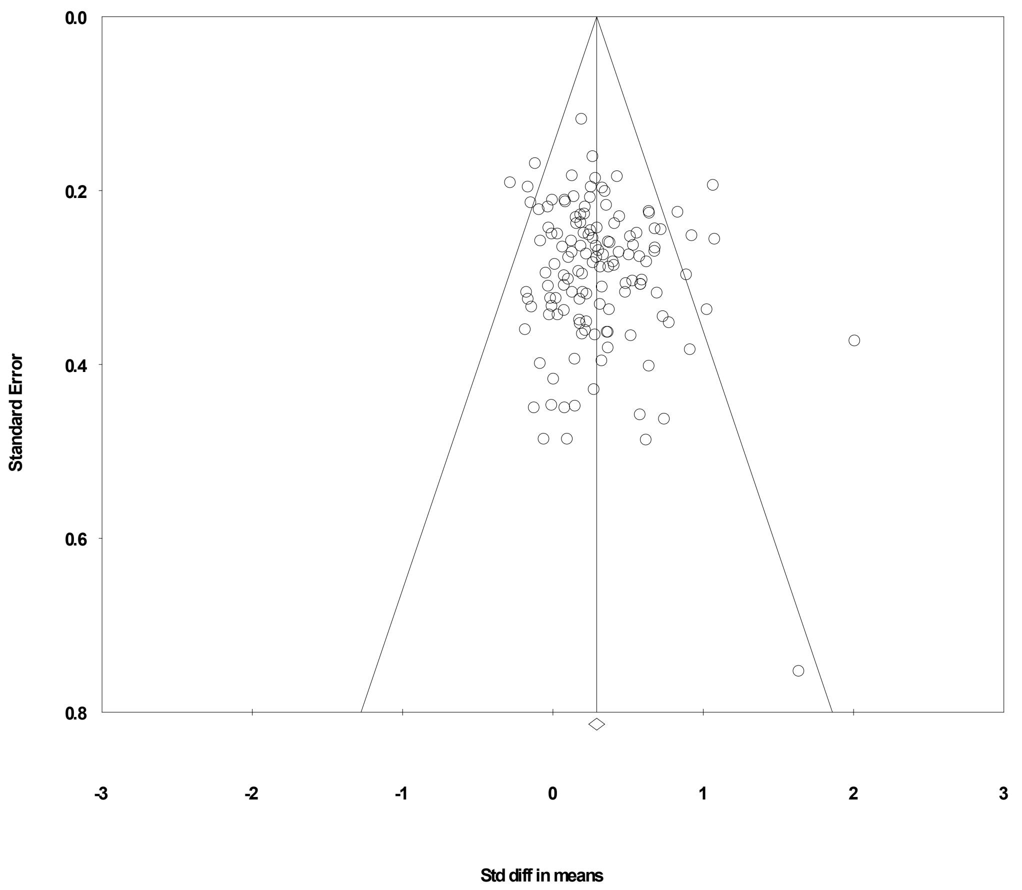

Test for asymmetry of funnel plot (linear regression of effect estimates on their standard errors weighted by their inverse variance – Egger test):

	N	Coefficient	SE	95% CI (lower limit)	95% CI (upper limit)	p value
Intercept	135	0.620	0.375	-0.121	1.362	0.100

© 2021 Vita et al. *JAMA Psychiatry.*

eFigure 9. Analysis of publication bias – Funnel plot for global functioning.

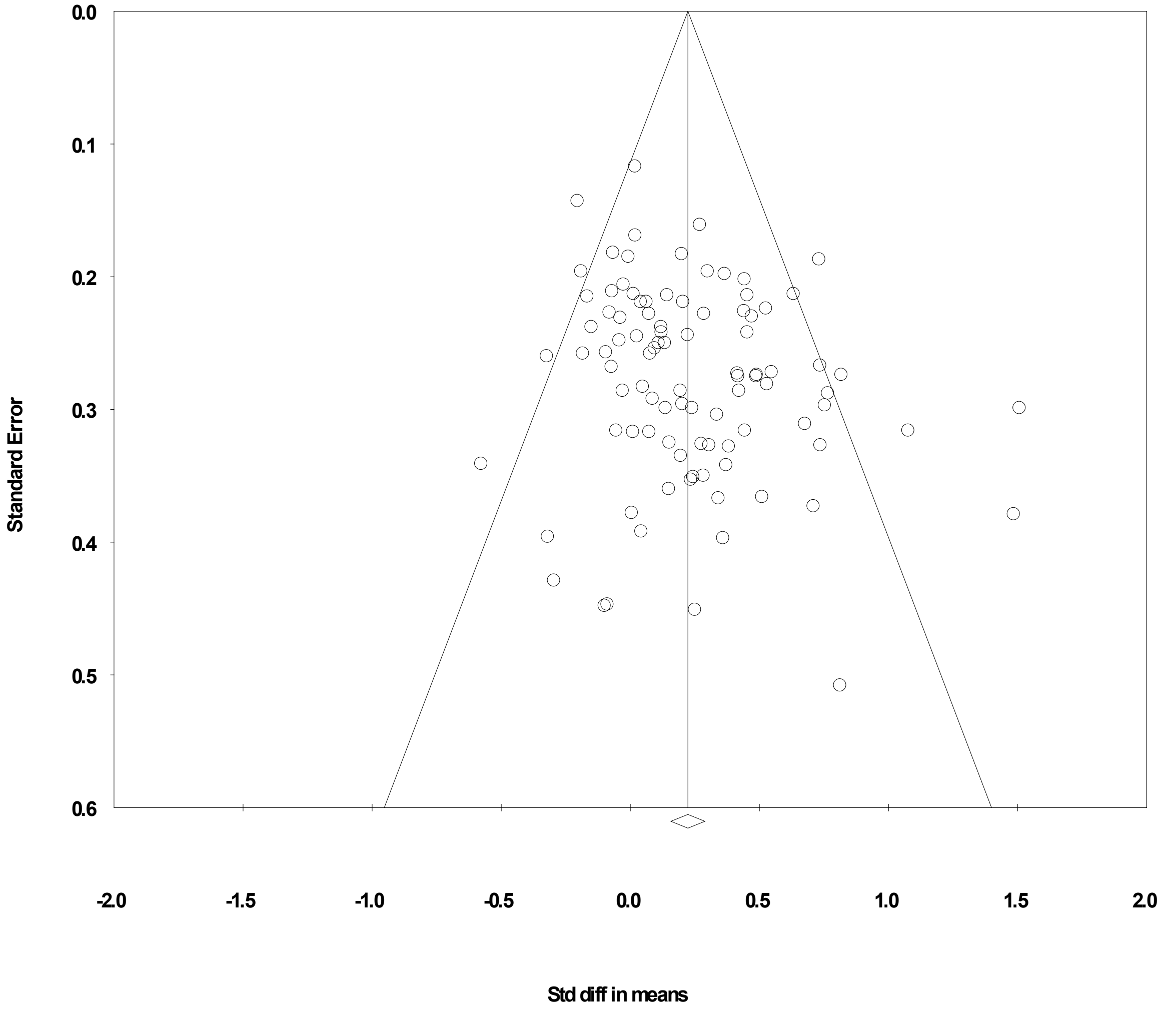

Test for asymmetry of funnel plot (linear regression of effect estimates on their standard errors weighted by their inverse variance – Egger test):

	N	Coefficient	SE	95% CI (lower limit)	95% CI (upper limit)	p value
Intercept	95	1.211	0.468	0.281	2.140	0.011

Adjustment of effect estimates using the Duval and Tweedie trim and fill method (studies missing to the left of the mean; random-random effects model):

	N	Point Estimate	95% CI (lower limit)	95% CI (upper limit)
Observed	95	0.223	0.157	0.289
Adjusted	+0	0.223	0.157	0.289

© 2021 Vita et al. *JAMA Psychiatry.*

Adjustment of effect estimates using the Duval and Tweedie trim and fill method (studies missing to the left of the mean; fixed-random effects model):

	N	Point Estimate	95% CI (lower limit)	95% CI (upper limit)
Observed	95	0.223	0.157	0.289
Adjusted	+20	0.104	0.028	0.180

A significant funnel plot asymmetry was observed at visual inspection and confirmed with the Egger test.
The trim and fill method, when applied with a random-random effect model, produced no adjustment of the effect estimate for studies missing on the left of the mean, while an adjustment comprising 20 studies was produced when using a fixed-random effect model.
The investigation of publication bias in the present work is complicated by the presence of heterogeneity between studies that is related to differences in methodological quality, in clinical settings and samples, in the implementation of interventions and in outcome measurement. Heterogeneity could represent a consistent explanation for the funnel plot asymmetry; this interpretation is also supported by the fact that subgroup analyses and meta-regressions based on methodological and clinical parameters (such as methodological quality, blinding of outcome assessment, intervention duration and implementation of CR with other psychosocial interventions) were found to be significant for functioning in particular. Furthermore, the investigation of functional outcome is intrinsically more prone to bias due to the assessment method (lack of standardized batteries, frequent use of self-report measures, investigation of different domains) and, in the studies included in the present work, it was more often evaluated by non—blind personnel, compared to cognition.
Small studies and feasibility trials represent a substantial proportion of the included studies, and it should be noted that the meta-regression on sample size revealed that this element was inversely related to the dimension of the effect detected on functioning. However, it is also true that in the field of psychosocial interventions large controlled trials are often hard to conduct, and small studies or pilot trials represent a typical situation for research on CR, which is implemented heterogeneously and inconsistently in clinical practice.
In this context, publication bias could represent one of the possible reasons for funnel plot asymmetry, but it is unlikely to be most prominent. Results of the trim-and-fill adjustment should therefore be interpreted cautiously and considered as one among the sensitivity analyses rather than as the “true” unbiased effect estimate.

© 2021 Vita et al. *JAMA Psychiatry.*
